# Supplementary material for: CTP promotes efficient ParB-dependent DNA condensation by facilitating one-dimensional diffusion from parS
Source: eLife. 2021 Jul 12;10:e67554. doi: 10.7554/eLife.67554 (PMC8299390; doi:10.7554/eLife.67554)
Supplement: Supplementary file 1. [file elife-67554-supp1.docx]

**Supplementary File 1. Sequences of DNA fragments used in this work.**

DNA sequences corresponding to the central part (not including the handles) of Magnetic Tweezers and C-trap DNA substrates employed in this work. Scrambled *parS* sequence is highlighted in pink. *ParS* sequences in inverted (TGTTTCACGTGGAACA) or direct (TGTTCCACGTGAAACA) orientation are highlighted in yellow. EcoRI restriction sites present in the central part of the C-trap substrate EcoRI 39x *parS* DNA are highlighted in red.

*MT ‘Scrambled’ parS DNA (5971 bp)*

GGCCGCCTGCATGTGTCAGAGGTTTTCACCGTCATCACCGAAACGCGCGAGGCAGCTGCGGTAAAGCTCATCAGCGTGGTCGTGAAGCGATTCACAGATGTCTGCCTGTTCATCCGCGTCCAGCTCGTTGAGTTTCTCCAGAAGCGTTAATGTCTGGCTTCTGATAAAGCGGGCCATGTTAAGGGCGGTTTTTTCCTGTTTGGTCACTGATGCCTCCGTGTAAGGGGGATTTCTGTTCATGGGGGTAATGATACCGATGAAACGAGAGAGGATGCTCACGATACGGGTTACTGATGATGAACATGCCCGGTTACTGGAACGTTGTGAGGGTAAACAACTGGCGGTATGGATGCGGCGGGACCAGAGAAAAATCACTCAGGGTCAATGCCAGCGCTTCGTTAATACAGATGTAGGTGTTCCACAGGGTAGCCAGCAGCATCCTGCGATGCAGATCCGGAACATAATGGTGCAGGGCGCTGACTTCCGCGTTTCCAGACTTTACGAAACACGGAAACCGAAGACCATTCATGTTGTTGCTCAGGTCGCAGACGTTTTGCAGCAGCAGTCGCTTCACGTTCGCTCGCGTATCGGTGATTCATTCTGCTAACCAGTAAGGCAACCCCGCCAGCCTAGCCGGGTCCTCAACGACAGGAGCACGATCATGCGCACCCGTGGGGCCGCCATGCCGGCGATAATGGCCTGCTTCTCGCCGAAACGTTTGGTGGCGGGACCAGTGACGAAGGCTTGAGCGAGGGCGTGCAAGATTCCGAATACCGCAAGCGACAGGCCGATCATCGTCGCGCTCCAGCGAAAGCGGTCCTCGCCGAAAATGACCCAGAGCGCTGCCGGCACCTGTCCTACGAGTTGCATGATAAAGAAGACAGTCATAAGTGCGGCGACGATAGTCATGCCCCGCGCCCACCGGAAGGAGCTGACTGGGTTGAAGGCTCTCAAGGGCATCGGTCGAGATCCCGGTGCCTAATGAGTGAGCTAACTTACATTAATTGCGTTGCGCTCACTGCCCGCTTTCCAGTCGGGAAACCTGTCGTGCCAGCTGCATTAATGAATCGGCCAACGCGCGGGGAGAGGCGGTTTGCGTATTGGGCGCCAGGGTGGTTTTTCTTTTCACCAGTGAGACGGGCAACAGCTGATTGCCCTTCACCGCCTGGCCCTGAGAGAGTTGCAGCAAGCGGTCCACGCTGGTTTGCCCCAGCAGGCGAAAATCCTGTTTGATGGTGGTTAACGGCGGGATATAACATGAGCTGTCTTCGGTATCGTCGTATCCCACTACCGAGATATCCGCACCAACGCGCAGCCCGGACTCGGTAATGGCGCGCATTGCGCCCAGCGCCATCTGATCGTTGGCAACCAGCATCGCAGTGGGAACGATGCCCTCATTCAGCATTTGCATGGTTTGTTGAAAACCGGACATGGCACTCCAGTCGCCTTCCCGTTCCGCTATCGGCTGAATTTGATTGCGAGTGAGATATTTATGCCAGCCAGCCAGACGCAGACGCGCCGAGACAGAACTTAATGGGCCCGCTAACAGCGCGATTTGCTGGTGACCCAATGCGACCAGATGCTCCACGCCCAGTCGCGTACCGTCTTCATGGGAGAAAATAATACTGTTGATGGGTGTCTGGTCAGAGACATCAAGAAATAACGCCGGAACATTAGTGCAGGCAGCTTCCACAGCAATGGCATCCTGGTCATCCAGCGGATAGTTAATGATCAGCCCACTGACGCGTTGCGCGAGAAGATTGTGCACCGCCGCTTTACAGGCTTCGACGCCGCTTCGTTCTACCATCGACACCACCACGCTGGCACCCAGTTGATCGGCGCGAGATTTAATCGCCGCGACAATTTGCGACGGCGCGTGCAGGGCCAGACTGGAGGTGGCAACGCCAATCAGCAACGACTGTTTGCCCGCCAGTTGTTGTGCCACGCGGTTGGGAATGTAATTCAGCTCCGCCATCGCCGCTTCCACTTTTTCCCGCGTTTTCGCAGAAACGTGGCTGGCCTGGTTCACCACGCGGGAAACGGTCTGATAAGAGACACCGGCATACTCTGCGACATCGTATAACGTTACTGGTTTCACATTCACCACCCTGAATTGACTCTCTTCCGGGCGCTATCATGCCATACCGCGAAAGGTTTTGCGCCATTCGATGGTGTCCGGGATCTCGACGCTCTCCCTTATGCGACTCCTGCATTAGGAAGCAGCCCAGTAGTAGGTTGAGGCCGTTGAGCACCGCCGCCGCAAGGAATGGTGCATGCAAGGAGATGGCGCCCAACAGTCCCCCGGCCACGGGGCCTGCCACCATACCCACGCCGAAACAAGCGCTCATGAGCCCGAAGTGGCGAGCCCGATCTTCCCCATCGGTGATGTCGGCGATATAGGCGCCAGCAACCGCACCTGTGGCGCCGGTGATGCCGGCCACGATGCGTCCGGCGTAGAGGATCGAGATCTCGATCCCGCGAAATTAATACGACTCACTATAGGGGAATTGTGAGCGGATAACAATTCCCCTCTAGAAATAATTTTGTTTAACTTTAAGAAGGAGATATACCATGGCTAAAGGCCTTGGAAAAGGGATTAATGCGTTATTTAATCAGGTAGATTTGTCTGAAGAGACAGTTGAAGAAATTAAAATTGCCGATTTACGCCCTAATCCTTATCAGCCAAGAAAACACTTTGATGACGAGGCATTAGCTGAACTAAAAGAATCTGTGCTGCAGCATGGCATTCTTCAGCCGCTTATCGTCAGAAAATCTTTAAAAGGCTATGATATTGTTGCGGGTGAACGGCGTTTTCGAGCGGCAAAGCTGGCAGGTTTAGATACAGTTCCGGCCATTGTCCGTGAATTATCAGAGGCGTTAATGAGGGAAATTGCTTTATTAGAAAACCTTCAGCGTGAAGATTTATCTCCGCTTGAAGAGGCTCAGGCATATGACTCCCTTTTGAAACACTTAGATCTCACACAAGAGCAGCTTGCCAAACGTCTTGGGAAAAGCAGACCGCATATTGCGAATCATTTAAGACTGCTGACACTGCCAGAAAATATTCAACAGCTTATTGCCGAAGGCACGCTTTCTATGGGACATGGACGCACGCTTCTTGGCTTAAAAAACAAAAATAAGCTTGAACCGCTGGTACAAAAAGTGATTGCGGAGCAGCTCAATGTTCGCCAACTTGAGCAGCTGATTCAGCAGTTGAATCAGAACGTGCCCAGGGAGACAAAGAAAAAAGAACCTGTGAAAGATGCGGTTCTAAAAGAACGGGAATCCTATCTCCAAAATTATTTTGGAACAACAGTTAATATTAAAAGACAGAAGAAAAAAGGCAAAATCGAAATTGAATTTTTCTCTAATGAAGACCTTGACCGGATTTTAGAGCTTTTGTCTGAACGAGAATCATAAGGATCCGAATTCGAGCTCCGTCGACAAGCTTCCGGCCGCAGTCGAGCACCACCACCACCACCACTGAGATCCGGCTGCTAACAAAGCCCGAAAGGAAGCTGAGTTGGCTGCTGCCACCGCTGAGCAATAACTAGCATAACCCCTTGGGGCCTCTAAACGGGTCTTGAGGGGTTTTTTGCTGAAAGGAGGAACTATATCCGGATTGGCGAATGGGACGCGCCCTGTAGCGGCGCATTAAGCGCGGCGGGTGTGGTGGTTACGCGCAGCGTGACCGCTACACTTGCCAGCGCCCTAGCGCCCGCTCCTTTCGCTTTCTTCCCTTCCTTTCTCGCCACGTTCGCCGGCTTTCCCCGTCAAGCTCTAAATCGGGGGCTCCCTTTAGGGTTCCGATTTAGTGCTTTACGGCACCTCGACCCCAAAAAACTTGATTAGGGTGATGGTTCACGTAGTGGGCCATCGCCCTGATAGACGGTTTTTCGCCCTTTGACGTTGGAGTCCACGTTCTTTAATAGTGGACTCTTGTTCCAAACTGGAACAACACTCAACCCTATCTCGGTCTATTCTTTTGATTTATAAGGGATTTTGCCGATTTCGGCCTATTGGTTAAAAAATGAGCTGATTTAACAAAAATTTAACGCGAATTTTAACAAAATATTAACGTTTACAATTTCAGGTGGCACTTTTCGGGGAAATGTGCGCGGAACCCCTATTTGTTTATTTTTCTAAATACATTCAAATATGTATCCGCTCATGAATTAATTCTTAGAAAAACTCATCGAGCATCAAATGAAACTGCAATTTATTCATATCAGGATTATCAATACCATATTTTTGAAAAAGCCGTTTCTGTAATGAAGGAGAAAACTCACCGAGGCAGTTCCATAGGATGGCAAGATCCTGGTATCGGTCTGCGATTCCGACTCGTCCAACATCAATACAACCTATTAATTTCCCCTCGTCAAAAATAAGGTTATCAAGTGAGAAATCACCATGAGTGACGACTGAATCCGGTGAGAATGGCAAAAGTTTATGCATTTCTTTCCAGACTTGTTCAACAGGCCAGCCATTACGCTCGTCATCAAAATCACTCGCATCAACCAAACCGTTATTCATTCGTGATTGCGCCTGAGCGAGACGAAATACGCGATCGCTGTTAAAAGGACAATTACAAACAGGAATCGAATGCAACCGGCGCAGGAACACTGCCAGCGCATCAACAATATTTTCACCTGAATCAGGATATTCTTCTAATACCTGGAATGCTGTTTTCCCGGGGATCGCAGTGGTGAGTAACCATGCATCATCAGGAGTACGGATAAAATGCTTGATGGTCGGAAGAGGCATAAATTCCGTCAGCCAGTTTAGTCTGACCATCTCATCTGTAACATCATTGGCAACGCTACCTTTGCCATGTTTCAGAAACAACTCTGGCGCATCGGGCTTCCCATACAATCGATAGATTGTCGCACCTGATTGCCCGACATTATCGCGAGCCCATTTATACCCATATAAATCAGCATCCATGTTGGAATTTAATCGCGGCCTAGAGCAAGACGTTTCCCGTTGAATATGGCTCATAACACCCCTTGTATTACTGTTTATGTAAGCAGACAGTTTTATTGTTCATGACCAAAATCCCTTAACGTGAGTTTTCGTTCCACTGAGCGTCAGACCCCGTAGAAAAGATCAAAGGATCTTCTTGAGATCCTTTTTTTCTGCGCGTAATCTGCTGCTTGCAAACAAAAAAACCACCGCTACCAGCGGTGGTTTGTTTGCCGGATCAAGAGCTACCAACTCTTTTTCCGAAGGTAACTGGCTTCAGCAGAGCGCAGATACCAAATACTGTCCTTCTAGTGTAGCCGTAGTTAGGCCACCACTTCAAGAACTCTGTAGCACCGCCTACATACCTCGCTCTGCTAATCCTGTTACCAGTGGCTGCTGCCAGTGGCGATAAGTCGTGTCTTACCGGGTTGGACTCAAGACGATAGTTACCGGATAAGGCGCAGCGGTCGGGCTGAACGGGGGGTTCGTGCACACAGCCCAGCTTGGAGCGAACGACCTACACCGAACTGAGATACCTACAGCGTGAGCTATGAGAAAGCGCCACGCTTCCCGAAGGGAGAAAGGCGGACAGGTATCCGGTAAGCGGCAGGGTCGGAACAGGAGAGCGCACGAGGGAGCTTCCAGGGGGAAACGCCTGGTATCTTTATAGTCCTGTCGGGTTTCGCCACCTCTGACTTGAGCGTCGATTTTTGTGATGCTCGTCAGGGGGGCGGAGCCTATGGAAAAACGCCAGCAACGCGGCCTTTTTACGGTTCCTGGCCTTTTGCTGGCCTTTTGCTCACATGTTCTTTCCTGCGTTATCCCCTGATTCTGTGGATAACCGTATTACCGCCTTTGAGTGAGCTGATACCGCTCGCCGCAGCCGAACGACCGAGCGCAGCGAGTCAGTGAGCGAGGAAGCGGAAGAGCGCCTGATGCGGTATTTTCTCCTTACGCATCTGTGCGGTATTTCACACCGCATATATGGTGCACTCTCAGTACAATCTGC

*MT 1x parS DNA (5971 bp)*

GGCCGCCTGCATGTGTCAGAGGTTTTCACCGTCATCACCGAAACGCGCGAGGCAGCTGCGGTAAAGCTCATCAGCGTGGTCGTGAAGCGATTCACAGATGTCTGCCTGTTCATCCGCGTCCAGCTCGTTGAGTTTCTCCAGAAGCGTTAATGTCTGGCTTCTGATAAAGCGGGCCATGTTAAGGGCGGTTTTTTCCTGTTTGGTCACTGATGCCTCCGTGTAAGGGGGATTTCTGTTCATGGGGGTAATGATACCGATGAAACGAGAGAGGATGCTCACGATACGGGTTACTGATGATGAACATGCCCGGTTACTGGAACGTTGTGAGGGTAAACAACTGGCGGTATGGATGCGGCGGGACCAGAGAAAAATCACTCAGGGTCAATGCCAGCGCTTCGTTAATACAGATGTAGGTGTTCCACAGGGTAGCCAGCAGCATCCTGCGATGCAGATCCGGAACATAATGGTGCAGGGCGCTGACTTCCGCGTTTCCAGACTTTACGAAACACGGAAACCGAAGACCATTCATGTTGTTGCTCAGGTCGCAGACGTTTTGCAGCAGCAGTCGCTTCACGTTCGCTCGCGTATCGGTGATTCATTCTGCTAACCAGTAAGGCAACCCCGCCAGCCTAGCCGGGTCCTCAACGACAGGAGCACGATCATGCGCACCCGTGGGGCCGCCATGCCGGCGATAATGGCCTGCTTCTCGCCGAAACGTTTGGTGGCGGGACCAGTGACGAAGGCTTGAGCGAGGGCGTGCAAGATTCCGAATACCGCAAGCGACAGGCCGATCATCGTCGCGCTCCAGCGAAAGCGGTCCTCGCCGAAAATGACCCAGAGCGCTGCCGGCACCTGTCCTACGAGTTGCATGATAAAGAAGACAGTCATAAGTGCGGCGACGATAGTCATGCCCCGCGCCCACCGGAAGGAGCTGACTGGGTTGAAGGCTCTCAAGGGCATCGGTCGAGATCCCGGTGCCTAATGAGTGAGCTAACTTACATTAATTGCGTTGCGCTCACTGCCCGCTTTCCAGTCGGGAAACCTGTCGTGCCAGCTGCATTAATGAATCGGCCAACGCGCGGGGAGAGGCGGTTTGCGTATTGGGCGCCAGGGTGGTTTTTCTTTTCACCAGTGAGACGGGCAACAGCTGATTGCCCTTCACCGCCTGGCCCTGAGAGAGTTGCAGCAAGCGGTCCACGCTGGTTTGCCCCAGCAGGCGAAAATCCTGTTTGATGGTGGTTAACGGCGGGATATAACATGAGCTGTCTTCGGTATCGTCGTATCCCACTACCGAGATATCCGCACCAACGCGCAGCCCGGACTCGGTAATGGCGCGCATTGCGCCCAGCGCCATCTGATCGTTGGCAACCAGCATCGCAGTGGGAACGATGCCCTCATTCAGCATTTGCATGGTTTGTTGAAAACCGGACATGGCACTCCAGTCGCCTTCCCGTTCCGCTATCGGCTGAATTTGATTGCGAGTGAGATATTTATGCCAGCCAGCCAGACGCAGACGCGCCGAGACAGAACTTAATGGGCCCGCTAACAGCGCGATTTGCTGGTGACCCAATGCGACCAGATGCTCCACGCCCAGTCGCGTACCGTCTTCATGGGAGAAAATAATACTGTTGATGGGTGTCTGGTCAGAGACATCAAGAAATAACGCCGGAACATTAGTGCAGGCAGCTTCCACAGCAATGGCATCCTGGTCATCCAGCGGATAGTTAATGATCAGCCCACTGACGCGTTGCGCGAGAAGATTGTGCACCGCCGCTTTACAGGCTTCGACGCCGCTTCGTTCTACCATCGACACCACCACGCTGGCACCCAGTTGATCGGCGCGAGATTTAATCGCCGCGACAATTTGCGACGGCGCGTGCAGGGCCAGACTGGAGGTGGCAACGCCAATCAGCAACGACTGTTTGCCCGCCAGTTGTTGTGCCACGCGGTTGGGAATGTAATTCAGCTCCGCCATCGCCGCTTCCACTTTTTCCCGCGTTTTCGCAGAAACGTGGCTGGCCTGGTTCACCACGCGGGAAACGGTCTGATAAGAGACACCGGCATACTCTGCGACATCGTATAACGTTACTGGTTTCACATTCACCACCCTGAATTGACTCTCTTCCGGGCGCTATCATGCCATACCGCGAAAGGTTTTGCGCCATTCGATGGTGTCCGGGATCTCGACGCTCTCCCTTATGCGACTCCTGCATTAGGAAGCAGCCCAGTAGTAGGTTGAGGCCGTTGAGCACCGCCGCCGCAAGGAATGGTGCATGCAAGGAGATGGCGCCCAACAGTCCCCCGGCCACGGGGCCTGCCACCATACCCACGCCGAAACAAGCGCTCATGAGCCCGAAGTGGCGAGCCCGATCTTCCCCATCGGTGATGTCGGCGATATAGGCGCCAGCAACCGCACCTGTGGCGCCGGTGATGCCGGCCACGATGCGTCCGGCGTAGAGGATCGAGATCTCGATCCCGCGAAATTAATACGACTCACTATAGGGGAATTGTGAGCGGATAACAATTCCCCTCTAGAAATAATTTTGTTTAACTTTAAGAAGGAGATATACCATGGCTAAAGGCCTTGGAAAAGGGATTAATGCGTTATTTAATCAGGTAGATTTGTCTGAAGAGACAGTTGAAGAAATTAAAATTGCCGATTTACGCCCTAATCCTTATCAGCCAAGAAAACACTTTGATGACGAGGCATTAGCTGAACTAAAAGAATCTGTGCTGCAGCATGGCATTCTTCAGCCGCTTATCGTCAGAAAATCTTTAAAAGGCTATGATATTGTTGCGGGTGAACGGCGTTTTCGAGCGGCAAAGCTGGCAGGTTTAGATACAGTTCCGGCCATTGTCCGTGAATTATCAGAGGCGTTAATGAGGGAAATTGCTTTATTAGAAAACCTTCAGCGTGAAGATTTATCTCCGCTTGAAGAGGCTCAGGCATATGACTCCCTTTTGAAACACTTAGATCTCACACAAGAGCAGCTTGCCAAACGTCTTGGGAAAAGCAGACCGCATATTGCGAATCATTTAAGACTGCTGACACTGCCAGAAAATATTCAACAGCTTATTGCCGAAGGCACGCTTTCTATGGGACATGGACGCACGCTTCTTGGCTTAAAAAACAAAAATAAGCTTGAACCGCTGGTACAAAAAGTGATTGCGGAGCAGCTCAATGTTCGCCAACTTGAGCAGCTGATTCAGCAGTTGAATCAGAATGTTCCACGTGAAACAAAGAAAAAAGAACCTGTGAAAGATGCGGTTCTAAAAGAACGGGAATCCTATCTCCAAAATTATTTTGGAACAACAGTTAATATTAAAAGACAGAAGAAAAAAGGCAAAATCGAAATTGAATTTTTCTCTAATGAAGACCTTGACCGGATTTTAGAGCTTTTGTCTGAACGAGAATCATAAGGATCCGAATTCGAGCTCCGTCGACAAGCTTCCGGCCGCAGTCGAGCACCACCACCACCACCACTGAGATCCGGCTGCTAACAAAGCCCGAAAGGAAGCTGAGTTGGCTGCTGCCACCGCTGAGCAATAACTAGCATAACCCCTTGGGGCCTCTAAACGGGTCTTGAGGGGTTTTTTGCTGAAAGGAGGAACTATATCCGGATTGGCGAATGGGACGCGCCCTGTAGCGGCGCATTAAGCGCGGCGGGTGTGGTGGTTACGCGCAGCGTGACCGCTACACTTGCCAGCGCCCTAGCGCCCGCTCCTTTCGCTTTCTTCCCTTCCTTTCTCGCCACGTTCGCCGGCTTTCCCCGTCAAGCTCTAAATCGGGGGCTCCCTTTAGGGTTCCGATTTAGTGCTTTACGGCACCTCGACCCCAAAAAACTTGATTAGGGTGATGGTTCACGTAGTGGGCCATCGCCCTGATAGACGGTTTTTCGCCCTTTGACGTTGGAGTCCACGTTCTTTAATAGTGGACTCTTGTTCCAAACTGGAACAACACTCAACCCTATCTCGGTCTATTCTTTTGATTTATAAGGGATTTTGCCGATTTCGGCCTATTGGTTAAAAAATGAGCTGATTTAACAAAAATTTAACGCGAATTTTAACAAAATATTAACGTTTACAATTTCAGGTGGCACTTTTCGGGGAAATGTGCGCGGAACCCCTATTTGTTTATTTTTCTAAATACATTCAAATATGTATCCGCTCATGAATTAATTCTTAGAAAAACTCATCGAGCATCAAATGAAACTGCAATTTATTCATATCAGGATTATCAATACCATATTTTTGAAAAAGCCGTTTCTGTAATGAAGGAGAAAACTCACCGAGGCAGTTCCATAGGATGGCAAGATCCTGGTATCGGTCTGCGATTCCGACTCGTCCAACATCAATACAACCTATTAATTTCCCCTCGTCAAAAATAAGGTTATCAAGTGAGAAATCACCATGAGTGACGACTGAATCCGGTGAGAATGGCAAAAGTTTATGCATTTCTTTCCAGACTTGTTCAACAGGCCAGCCATTACGCTCGTCATCAAAATCACTCGCATCAACCAAACCGTTATTCATTCGTGATTGCGCCTGAGCGAGACGAAATACGCGATCGCTGTTAAAAGGACAATTACAAACAGGAATCGAATGCAACCGGCGCAGGAACACTGCCAGCGCATCAACAATATTTTCACCTGAATCAGGATATTCTTCTAATACCTGGAATGCTGTTTTCCCGGGGATCGCAGTGGTGAGTAACCATGCATCATCAGGAGTACGGATAAAATGCTTGATGGTCGGAAGAGGCATAAATTCCGTCAGCCAGTTTAGTCTGACCATCTCATCTGTAACATCATTGGCAACGCTACCTTTGCCATGTTTCAGAAACAACTCTGGCGCATCGGGCTTCCCATACAATCGATAGATTGTCGCACCTGATTGCCCGACATTATCGCGAGCCCATTTATACCCATATAAATCAGCATCCATGTTGGAATTTAATCGCGGCCTAGAGCAAGACGTTTCCCGTTGAATATGGCTCATAACACCCCTTGTATTACTGTTTATGTAAGCAGACAGTTTTATTGTTCATGACCAAAATCCCTTAACGTGAGTTTTCGTTCCACTGAGCGTCAGACCCCGTAGAAAAGATCAAAGGATCTTCTTGAGATCCTTTTTTTCTGCGCGTAATCTGCTGCTTGCAAACAAAAAAACCACCGCTACCAGCGGTGGTTTGTTTGCCGGATCAAGAGCTACCAACTCTTTTTCCGAAGGTAACTGGCTTCAGCAGAGCGCAGATACCAAATACTGTCCTTCTAGTGTAGCCGTAGTTAGGCCACCACTTCAAGAACTCTGTAGCACCGCCTACATACCTCGCTCTGCTAATCCTGTTACCAGTGGCTGCTGCCAGTGGCGATAAGTCGTGTCTTACCGGGTTGGACTCAAGACGATAGTTACCGGATAAGGCGCAGCGGTCGGGCTGAACGGGGGGTTCGTGCACACAGCCCAGCTTGGAGCGAACGACCTACACCGAACTGAGATACCTACAGCGTGAGCTATGAGAAAGCGCCACGCTTCCCGAAGGGAGAAAGGCGGACAGGTATCCGGTAAGCGGCAGGGTCGGAACAGGAGAGCGCACGAGGGAGCTTCCAGGGGGAAACGCCTGGTATCTTTATAGTCCTGTCGGGTTTCGCCACCTCTGACTTGAGCGTCGATTTTTGTGATGCTCGTCAGGGGGGCGGAGCCTATGGAAAAACGCCAGCAACGCGGCCTTTTTACGGTTCCTGGCCTTTTGCTGGCCTTTTGCTCACATGTTCTTTCCTGCGTTATCCCCTGATTCTGTGGATAACCGTATTACCGCCTTTGAGTGAGCTGATACCGCTCGCCGCAGCCGAACGACCGAGCGCAGCGAGTCAGTGAGCGAGGAAGCGGAAGAGCGCCTGATGCGGTATTTTCTCCTTACGCATCTGTGCGGTATTTCACACCGCATATATGGTGCACTCTCAGTACAATCTGC

*MT 2x parS DNA (5991 bp)*

GGCCGCCTGCATGTGTCAGAGGTTTTCACCGTCATCACCGAAACGCGCGAGGCAGCTGCGGTAAAGCTCATCAGCGTGGTCGTGAAGCGATTCACAGATGTCTGCCTGTTCATCCGCGTCCAGCTCGTTGAGTTTCTCCAGAAGCGTTAATGTCTGGCTTCTGATAAAGCGGGCCATGTTAAGGGCGGTTTTTTCCTGTTTGGTCACTGATGCCTCCGTGTAAGGGGGATTTCTGTTCATGGGGGTAATGATACCGATGAAACGAGAGAGGATGCTCACGATACGGGTTACTGATGATGAACATGCCCGGTTACTGGAACGTTGTGAGGGTAAACAACTGGCGGTATGGATGCGGCGGGACCAGAGAAAAATCACTCAGGGTCAATGCCAGCGCTTCGTTAATACAGATGTAGGTGTTCCACAGGGTAGCCAGCAGCATCCTGCGATGCAGATCCGGAACATAATGGTGCAGGGCGCTGACTTCCGCGTTTCCAGACTTTACGAAACACGGAAACCGAAGACCATTCATGTTGTTGCTCAGGTCGCAGACGTTTTGCAGCAGCAGTCGCTTCACGTTCGCTCGCGTATCGGTGATTCATTCTGCTAACCAGTAAGGCAACCCCGCCAGCCTAGCCGGGTCCTCAACGACAGGAGCACGATCATGCGCACCCGTGGGGCCGCCATGCCGGCGATAATGGCCTGCTTCTCGCCGAAACGTTTGGTGGCGGGACCAGTGACGAAGGCTTGAGCGAGGGCGTGCAAGATTCCGAATACCGCAAGCGACAGGCCGATCATCGTCGCGCTCCAGCGAAAGCGGTCCTCGCCGAAAATGACCCAGAGCGCTGCCGGCACCTGTCCTACGAGTTGCATGATAAAGAAGACAGTCATAAGTGCGGCGACGATAGTCATGCCCCGCGCCCACCGGAAGGAGCTGACTGGGTTGAAGGCTCTCAAGGGCATCGGTCGAGATCCCGGTGCCTAATGAGTGAGCTAACTTACATTAATTGCGTTGCGCTCACTGCCCGCTTTCCAGTCGGGAAACCTGTCGTGCCAGCTGCATTAATGAATCGGCCAACGCGCGGGGAGAGGCGGTTTGCGTATTGGGCGCCAGGGTGGTTTTTCTTTTCACCAGTGAGACGGGCAACAGCTGATTGCCCTTCACCGCCTGGCCCTGAGAGAGTTGCAGCAAGCGGTCCACGCTGGTTTGCCCCAGCAGGCGAAAATCCTGTTTGATGGTGGTTAACGGCGGGATATAACATGAGCTGTCTTCGGTATCGTCGTATCCCACTACCGAGATATCCGCACCAACGCGCAGCCCGGACTCGGTAATGGCGCGCATTGCGCCCAGCGCCATCTGATCGTTGGCAACCAGCATCGCAGTGGGAACGATGCCCTCATTCAGCATTTGCATGGTTTGTTGAAAACCGGACATGGCACTCCAGTCGCCTTCCCGTTCCGCTATCGGCTGAATTTGATTGCGAGTGAGATATTTATGCCAGCCAGCCAGACGCAGACGCGCCGAGACAGAACTTAATGGGCCCGCTAACAGCGCGATTTGCTGGTGACCCAATGCGACCAGATGCTCCACGCCCAGTCGCGTACCGTCTTCATGGGAGAAAATAATACTGTTGATGGGTGTCTGGTCAGAGACATCAAGAAATAACGCCGGAACATTAGTGCAGGCAGCTTCCACAGCAATGGCATCCTGGTCATCCAGCGGATAGTTAATGATCAGCCCACTGACGCGTTGCGCGAGAAGATTGTGCACCGCCGCTTTACAGGCTTCGACGCCGCTTCGTTCTACCATCGACACCACCACGCTGGCACCCAGTTGATCGGCGCGAGATTTAATCGCCGCGACAATTTGCGACGGCGCGTGCAGGGCCAGACTGGAGGTGGCAACGCCAATCAGCAACGACTGTTTGCCCGCCAGTTGTTGTGCCACGCGGTTGGGAATGTAATTCAGCTCCGCCATCGCCGCTTCCACTTTTTCCCGCGTTTTCGCAGAAACGTGGCTGGCCTGGTTCACCACGCGGGAAACGGTCTGATAAGAGACACCGGCATACTCTGCGACATCGTATAACGTTACTGGTTTCACATTCACCACCCTGAATTGACTCTCTTCCGGGCGCTATCATGCCATACCGCGAAAGGTTTTGCGCCATTCGATGGTGTCCGGGATCTCGACGCTCTCCCTTATGCGACTCCTGCATTAGGAAGCAGCCCAGTAGTAGGTTGAGGCCGTTGAGCACCGCCGCCGCAAGGAATGGTGCATGCAAGGAGATGGCGCCCAACAGTCCCCCGGCCACGGGGCCTGCCACCATACCCACGCCGAAACAAGCGCTCATGAGCCCGAAGTGGCGAGCCCGATCTTCCCCATCGGTGATGTCGGCGATATAGGCGCCAGCAACCGCACCTGTGGCGCCGGTGATGCCGGCCACGATGCGTCCGGCGTAGAGGATCGAGATCTCGATCCCGCGAAATTAATACGACTCACTATAGGGGAATTGTGAGCGGATAACAATTCCCCTCTAGAAATAATTTTGTTTAACTTTAAGAAGGAGATATACCATGTGTTTCACGTGGAACACATGGCTAAAGGCCTTGGAAAAGGGATTAATGCGTTATTTAATCAGGTAGATTTGTCTGAAGAGACAGTTGAAGAAATTAAAATTGCCGATTTACGCCCTAATCCTTATCAGCCAAGAAAACACTTTGATGACGAGGCATTAGCTGAACTAAAAGAATCTGTGCTGCAGCATGGCATTCTTCAGCCGCTTATCGTCAGAAAATCTTTAAAAGGCTATGATATTGTTGCGGGTGAACGGCGTTTTCGAGCGGCAAAGCTGGCAGGTTTAGATACAGTTCCGGCCATTGTCCGTGAATTATCAGAGGCGTTAATGAGGGAAATTGCTTTATTAGAAAACCTTCAGCGTGAAGATTTATCTCCGCTTGAAGAGGCTCAGGCATATGACTCCCTTTTGAAACACTTAGATCTCACACAAGAGCAGCTTGCCAAACGTCTTGGGAAAAGCAGACCGCATATTGCGAATCATTTAAGACTGCTGACACTGCCAGAAAATATTCAACAGCTTATTGCCGAAGGCACGCTTTCTATGGGACATGGACGCACGCTTCTTGGCTTAAAAAACAAAAATAAGCTTGAACCGCTGGTACAAAAAGTGATTGCGGAGCAGCTCAATGTTCGCCAACTTGAGCAGCTGATTCAGCAGTTGAATCAGAATGTTCCACGTGAAACAAAGAAAAAAGAACCTGTGAAAGATGCGGTTCTAAAAGAACGGGAATCCTATCTCCAAAATTATTTTGGAACAACAGTTAATATTAAAAGACAGAAGAAAAAAGGCAAAATCGAAATTGAATTTTTCTCTAATGAAGACCTTGACCGGATTTTAGAGCTTTTGTCTGAACGAGAATCATAAGGATCCGAATTCGAGCTCCGTCGACAAGCTTCCGGCCGCAGTCGAGCACCACCACCACCACCACTGAGATCCGGCTGCTAACAAAGCCCGAAAGGAAGCTGAGTTGGCTGCTGCCACCGCTGAGCAATAACTAGCATAACCCCTTGGGGCCTCTAAACGGGTCTTGAGGGGTTTTTTGCTGAAAGGAGGAACTATATCCGGATTGGCGAATGGGACGCGCCCTGTAGCGGCGCATTAAGCGCGGCGGGTGTGGTGGTTACGCGCAGCGTGACCGCTACACTTGCCAGCGCCCTAGCGCCCGCTCCTTTCGCTTTCTTCCCTTCCTTTCTCGCCACGTTCGCCGGCTTTCCCCGTCAAGCTCTAAATCGGGGGCTCCCTTTAGGGTTCCGATTTAGTGCTTTACGGCACCTCGACCCCAAAAAACTTGATTAGGGTGATGGTTCACGTAGTGGGCCATCGCCCTGATAGACGGTTTTTCGCCCTTTGACGTTGGAGTCCACGTTCTTTAATAGTGGACTCTTGTTCCAAACTGGAACAACACTCAACCCTATCTCGGTCTATTCTTTTGATTTATAAGGGATTTTGCCGATTTCGGCCTATTGGTTAAAAAATGAGCTGATTTAACAAAAATTTAACGCGAATTTTAACAAAATATTAACGTTTACAATTTCAGGTGGCACTTTTCGGGGAAATGTGCGCGGAACCCCTATTTGTTTATTTTTCTAAATACATTCAAATATGTATCCGCTCATGAATTAATTCTTAGAAAAACTCATCGAGCATCAAATGAAACTGCAATTTATTCATATCAGGATTATCAATACCATATTTTTGAAAAAGCCGTTTCTGTAATGAAGGAGAAAACTCACCGAGGCAGTTCCATAGGATGGCAAGATCCTGGTATCGGTCTGCGATTCCGACTCGTCCAACATCAATACAACCTATTAATTTCCCCTCGTCAAAAATAAGGTTATCAAGTGAGAAATCACCATGAGTGACGACTGAATCCGGTGAGAATGGCAAAAGTTTATGCATTTCTTTCCAGACTTGTTCAACAGGCCAGCCATTACGCTCGTCATCAAAATCACTCGCATCAACCAAACCGTTATTCATTCGTGATTGCGCCTGAGCGAGACGAAATACGCGATCGCTGTTAAAAGGACAATTACAAACAGGAATCGAATGCAACCGGCGCAGGAACACTGCCAGCGCATCAACAATATTTTCACCTGAATCAGGATATTCTTCTAATACCTGGAATGCTGTTTTCCCGGGGATCGCAGTGGTGAGTAACCATGCATCATCAGGAGTACGGATAAAATGCTTGATGGTCGGAAGAGGCATAAATTCCGTCAGCCAGTTTAGTCTGACCATCTCATCTGTAACATCATTGGCAACGCTACCTTTGCCATGTTTCAGAAACAACTCTGGCGCATCGGGCTTCCCATACAATCGATAGATTGTCGCACCTGATTGCCCGACATTATCGCGAGCCCATTTATACCCATATAAATCAGCATCCATGTTGGAATTTAATCGCGGCCTAGAGCAAGACGTTTCCCGTTGAATATGGCTCATAACACCCCTTGTATTACTGTTTATGTAAGCAGACAGTTTTATTGTTCATGACCAAAATCCCTTAACGTGAGTTTTCGTTCCACTGAGCGTCAGACCCCGTAGAAAAGATCAAAGGATCTTCTTGAGATCCTTTTTTTCTGCGCGTAATCTGCTGCTTGCAAACAAAAAAACCACCGCTACCAGCGGTGGTTTGTTTGCCGGATCAAGAGCTACCAACTCTTTTTCCGAAGGTAACTGGCTTCAGCAGAGCGCAGATACCAAATACTGTCCTTCTAGTGTAGCCGTAGTTAGGCCACCACTTCAAGAACTCTGTAGCACCGCCTACATACCTCGCTCTGCTAATCCTGTTACCAGTGGCTGCTGCCAGTGGCGATAAGTCGTGTCTTACCGGGTTGGACTCAAGACGATAGTTACCGGATAAGGCGCAGCGGTCGGGCTGAACGGGGGGTTCGTGCACACAGCCCAGCTTGGAGCGAACGACCTACACCGAACTGAGATACCTACAGCGTGAGCTATGAGAAAGCGCCACGCTTCCCGAAGGGAGAAAGGCGGACAGGTATCCGGTAAGCGGCAGGGTCGGAACAGGAGAGCGCACGAGGGAGCTTCCAGGGGGAAACGCCTGGTATCTTTATAGTCCTGTCGGGTTTCGCCACCTCTGACTTGAGCGTCGATTTTTGTGATGCTCGTCAGGGGGGCGGAGCCTATGGAAAAACGCCAGCAACGCGGCCTTTTTACGGTTCCTGGCCTTTTGCTGGCCTTTTGCTCACATGTTCTTTCCTGCGTTATCCCCTGATTCTGTGGATAACCGTATTACCGCCTTTGAGTGAGCTGATACCGCTCGCCGCAGCCGAACGACCGAGCGCAGCGAGTCAGTGAGCGAGGAAGCGGAAGAGCGCCTGATGCGGTATTTTCTCCTTACGCATCTGTGCGGTATTTCACACCGCATATATGGTGCACTCTCAGTACAATCTGC

*MT 4x parS DNA (6101 bp)*

GGCCGCCTGCATGTGTCAGAGGTTTTCACCGTCATCACCGAAACGCGCGAGGCAGCTGCGGTAAAGCTCATCAGCGTGGTCGTGAAGCGATTCACAGATGTCTGCCTGTTCATCCGCGTCCAGCTCGTTGAGTTTCTCCAGAAGCGTTAATGTCTGGCTTCTGATAAAGCGGGCCATGTTAAGGGCGGTTTTTTCCTGTTTGGTCACTGATGCCTCCGTGTAAGGGGGATTTCTGTTCATGGGGGTAATGATACCGATGAAACGAGAGAGGATGCTCACGATACGGGTTACTGATGATGAACATGCCCGGTTACTGGAACGTTGTGAGGGTAAACAACTGGCGGTATGGATGCGGCGGGACCAGAGAAAAATCACTCAGGGTCAATGCCAGCGCTTCGTTAATACAGATGTAGGTGTTCCACAGGGTAGCCAGCAGCATCCTGCGATGCAGATCCGGAACATAATGGTGCAGGGCGCTGACTTCCGCGTTTCCAGACTTTACGAAACACGGAAACCGAAGACCATTCATGTTGTTGCTCAGGTCGCAGACGTTTTGCAGCAGCAGTCGCTTCACGTTCGCTCGCGTATCGGTGATTCATTCTGCTAACCAGTAAGGCAACCCCGCCAGCCTAGCCGGGTCCTCAACGACAGGAGCACGATCATGCGCACCCGTGGGGCCGCCATGCCGGCGATAATGGCCTGCTTCTCGCCGAAACGTTTGGTGGCGGGACCAGTGACGAAGGCTTGAGCGAGGGCGTGCAAGATTCCGAATACCGCAAGCGACAGGCCGATCATCGTCGCGCTCCAGCGAAAGCGGTCCTCGCCGAAAATGACCCAGAGCGCTGCCGGCACCTGTCCTACGAGTTGCATGATAAAGAAGACAGTCATAAGTGCGGCGACGATAGTCATGCCCCGCGCCCACCGGAAGGAGCTGACTGGGTTGAAGGCTCTCAAGGGCATCGGTCGAGATCCCGGTGCCTAATGAGTGAGCTAACTTACATTAATTGCGTTGCGCTCACTGCCCGCTTTCCAGTCGGGAAACCTGTCGTGCCAGCTGCATTAATGAATCGGCCAACGCGCGGGGAGAGGCGGTTTGCGTATTGGGCGCCAGGGTGGTTTTTCTTTTCACCAGTGAGACGGGCAACAGCTGATTGCCCTTCACCGCCTGGCCCTGAGAGAGTTGCAGCAAGCGGTCCACGCTGGTTTGCCCCAGCAGGCGAAAATCCTGTTTGATGGTGGTTAACGGCGGGATATAACATGAGCTGTCTTCGGTATCGTCGTATCCCACTACCGAGATATCCGCACCAACGCGCAGCCCGGACTCGGTAATGGCGCGCATTGCGCCCAGCGCCATCTGATCGTTGGCAACCAGCATCGCAGTGGGAACGATGCCCTCATTCAGCATTTGCATGGTTTGTTGAAAACCGGACATGGCACTCCAGTCGCCTTCCCGTTCCGCTATCGGCTGAATTTGATTGCGAGTGAGATATTTATGCCAGCCAGCCAGACGCAGACGCGCCGAGACAGAACTTAATGGGCCCGCTAACAGCGCGATTTGCTGGTGACCCAATGCGACCAGATGCTCCACGCCCAGTCGCGTACCGTCTTCATGGGAGAAAATAATACTGTTGATGGGTGTCTGGTCAGAGACATCAAGAAATAACGCCGGAACATTAGTGCAGGCAGCTTCCACAGCAATGGCATCCTGGTCATCCAGCGGATAGTTAATGATCAGCCCACTGACGCGTTGCGCGAGAAGATTGTGCACCGCCGCTTTACAGGCTTCGACGCCGCTTCGTTCTACCATCGACACCACCACGCTGGCACCCAGTTGATCGGCGCGAGATTTAATCGCCGCGACAATTTGCGACGGCGCGTGCAGGGCCAGACTGGAGGTGGCAACGCCAATCAGCAACGACTGTTTGCCCGCCAGTTGTTGTGCCACGCGGTTGGGAATGTAATTCAGCTCCGCCATCGCCGCTTCCACTTTTTCCCGCGTTTTCGCAGAAACGTGGCTGGCCTGGTTCACCACGCGGGAAACGGTCTGATAAGAGACACCGGCATACTCTGCGACATCGTATAACGTTACTGGTTTCACATTCACCACCCTGAATTGACTCTCTTCCGGGCGCTATCATGCCATACCGCGAAAGGTTTTGCGCCATTCGATGGTGTCCGGGATCTCGACGCTCTCCCTTATGCGACTCCTGCATTAGGAAGCAGCCCAGTAGTAGGTTGAGGCCGTTGAGCACCGCCGCCGCAAGGAATGGTGCATGCAAGGAGATGGCGCCCAACAGTCCCCCGGCCACGGGGCCTGCCACCATACCCACGCCGAAACAAGCGCTCATGAGCCCGAAGTGGCGAGCCCGATCTTCCCCATCGGTGATGTCGGCGATATAGGCGCCAGCAACCGCACCTGTGGCGCCGGTGATGCCGGCCACGATGCGTCCGGCGTAGAGGATCGAGATCTCGATCCCGCGAAATTAATACGACTCACTATAGGGGAATTGTGAGCGGATAACAATTCCCCTCTAGCCATATTGGACTCGGACCTGTTTCACGTGGAACACTGAGCCTGGACTAGGTCTAGAACAGGTTCTTTTTTCTTTGTTTCACGTGGAACATTCTGATTAATGTACAGCTAGAAATAATTTTGTTTAACTTTAAGAAGGAGATATACCATGTGTTTCACGTGGAACACATGGCTAAAGGCCTTGGAAAAGGGATTAATGCGTTATTTAATCAGGTAGATTTGTCTGAAGAGACAGTTGAAGAAATTAAAATTGCCGATTTACGCCCTAATCCTTATCAGCCAAGAAAACACTTTGATGACGAGGCATTAGCTGAACTAAAAGAATCTGTGCTGCAGCATGGCATTCTTCAGCCGCTTATCGTCAGAAAATCTTTAAAAGGCTATGATATTGTTGCGGGTGAACGGCGTTTTCGAGCGGCAAAGCTGGCAGGTTTAGATACAGTTCCGGCCATTGTCCGTGAATTATCAGAGGCGTTAATGAGGGAAATTGCTTTATTAGAAAACCTTCAGCGTGAAGATTTATCTCCGCTTGAAGAGGCTCAGGCATATGACTCCCTTTTGAAACACTTAGATCTCACACAAGAGCAGCTTGCCAAACGTCTTGGGAAAAGCAGACCGCATATTGCGAATCATTTAAGACTGCTGACACTGCCAGAAAATATTCAACAGCTTATTGCCGAAGGCACGCTTTCTATGGGACATGGACGCACGCTTCTTGGCTTAAAAAACAAAAATAAGCTTGAACCGCTGGTACAAAAAGTGATTGCGGAGCAGCTCAATGTTCGCCAACTTGAGCAGCTGATTCAGCAGTTGAATCAGAATGTTCCACGTGAAACAAAGAAAAAAGAACCTGTGAAAGATGCGGTTCTAAAAGAACGGGAATCCTATCTCCAAAATTATTTTGGAACAACAGTTAATATTAAAAGACAGAAGAAAAAAGGCAAAATCGAAATTGAATTTTTCTCTAATGAAGACCTTGACCGGATTTTAGAGCTTTTGTCTGAACGAGAATCATAAGGATCCGAATTCGAGCTCCGTCGACAAGCTTCCGGCCGCAGTCGAGCACCACCACCACCACCACTGAGATCCGGCTGCTAACAAAGCCCGAAAGGAAGCTGAGTTGGCTGCTGCCACCGCTGAGCAATAACTAGCATAACCCCTTGGGGCCTCTAAACGGGTCTTGAGGGGTTTTTTGCTGAAAGGAGGAACTATATCCGGATTGGCGAATGGGACGCGCCCTGTAGCGGCGCATTAAGCGCGGCGGGTGTGGTGGTTACGCGCAGCGTGACCGCTACACTTGCCAGCGCCCTAGCGCCCGCTCCTTTCGCTTTCTTCCCTTCCTTTCTCGCCACGTTCGCCGGCTTTCCCCGTCAAGCTCTAAATCGGGGGCTCCCTTTAGGGTTCCGATTTAGTGCTTTACGGCACCTCGACCCCAAAAAACTTGATTAGGGTGATGGTTCACGTAGTGGGCCATCGCCCTGATAGACGGTTTTTCGCCCTTTGACGTTGGAGTCCACGTTCTTTAATAGTGGACTCTTGTTCCAAACTGGAACAACACTCAACCCTATCTCGGTCTATTCTTTTGATTTATAAGGGATTTTGCCGATTTCGGCCTATTGGTTAAAAAATGAGCTGATTTAACAAAAATTTAACGCGAATTTTAACAAAATATTAACGTTTACAATTTCAGGTGGCACTTTTCGGGGAAATGTGCGCGGAACCCCTATTTGTTTATTTTTCTAAATACATTCAAATATGTATCCGCTCATGAATTAATTCTTAGAAAAACTCATCGAGCATCAAATGAAACTGCAATTTATTCATATCAGGATTATCAATACCATATTTTTGAAAAAGCCGTTTCTGTAATGAAGGAGAAAACTCACCGAGGCAGTTCCATAGGATGGCAAGATCCTGGTATCGGTCTGCGATTCCGACTCGTCCAACATCAATACAACCTATTAATTTCCCCTCGTCAAAAATAAGGTTATCAAGTGAGAAATCACCATGAGTGACGACTGAATCCGGTGAGAATGGCAAAAGTTTATGCATTTCTTTCCAGACTTGTTCAACAGGCCAGCCATTACGCTCGTCATCAAAATCACTCGCATCAACCAAACCGTTATTCATTCGTGATTGCGCCTGAGCGAGACGAAATACGCGATCGCTGTTAAAAGGACAATTACAAACAGGAATCGAATGCAACCGGCGCAGGAACACTGCCAGCGCATCAACAATATTTTCACCTGAATCAGGATATTCTTCTAATACCTGGAATGCTGTTTTCCCGGGGATCGCAGTGGTGAGTAACCATGCATCATCAGGAGTACGGATAAAATGCTTGATGGTCGGAAGAGGCATAAATTCCGTCAGCCAGTTTAGTCTGACCATCTCATCTGTAACATCATTGGCAACGCTACCTTTGCCATGTTTCAGAAACAACTCTGGCGCATCGGGCTTCCCATACAATCGATAGATTGTCGCACCTGATTGCCCGACATTATCGCGAGCCCATTTATACCCATATAAATCAGCATCCATGTTGGAATTTAATCGCGGCCTAGAGCAAGACGTTTCCCGTTGAATATGGCTCATAACACCCCTTGTATTACTGTTTATGTAAGCAGACAGTTTTATTGTTCATGACCAAAATCCCTTAACGTGAGTTTTCGTTCCACTGAGCGTCAGACCCCGTAGAAAAGATCAAAGGATCTTCTTGAGATCCTTTTTTTCTGCGCGTAATCTGCTGCTTGCAAACAAAAAAACCACCGCTACCAGCGGTGGTTTGTTTGCCGGATCAAGAGCTACCAACTCTTTTTCCGAAGGTAACTGGCTTCAGCAGAGCGCAGATACCAAATACTGTCCTTCTAGTGTAGCCGTAGTTAGGCCACCACTTCAAGAACTCTGTAGCACCGCCTACATACCTCGCTCTGCTAATCCTGTTACCAGTGGCTGCTGCCAGTGGCGATAAGTCGTGTCTTACCGGGTTGGACTCAAGACGATAGTTACCGGATAAGGCGCAGCGGTCGGGCTGAACGGGGGGTTCGTGCACACAGCCCAGCTTGGAGCGAACGACCTACACCGAACTGAGATACCTACAGCGTGAGCTATGAGAAAGCGCCACGCTTCCCGAAGGGAGAAAGGCGGACAGGTATCCGGTAAGCGGCAGGGTCGGAACAGGAGAGCGCACGAGGGAGCTTCCAGGGGGAAACGCCTGGTATCTTTATAGTCCTGTCGGGTTTCGCCACCTCTGACTTGAGCGTCGATTTTTGTGATGCTCGTCAGGGGGGCGGAGCCTATGGAAAAACGCCAGCAACGCGGCCTTTTTACGGTTCCTGGCCTTTTGCTGGCCTTTTGCTCACATGTTCTTTCCTGCGTTATCCCCTGATTCTGTGGATAACCGTATTACCGCCTTTGAGTGAGCTGATACCGCTCGCCGCAGCCGAACGACCGAGCGCAGCGAGTCAGTGAGCGAGGAAGCGGAAGAGCGCCTGATGCGGTATTTTCTCCTTACGCATCTGTGCGGTATTTCACACCGCATATATGGTGCACTCTCAGTACAATCTGC

*MT 7x parS DNA (6316 bp)*

GGCCGCCTGCATGTGTCAGAGGTTTTCACCGTCATCACCGAAACGCGCGAGGCAGCTGCGGTAAAGCTCATCAGCGTGGTCGTGAAGCGATTCACAGATGTCTGCCTGTTCATCCGCGTCCAGCTCGTTGAGTTTCTCCAGAAGCGTTAATGTCTGGCTTCTGATAAAGCGGGCCATGTTAAGGGCGGTTTTTTCCTGTTTGGTCACTGATGCCTCCGTGTAAGGGGGATTTCTGTTCATGGGGGTAATGATACCGATGAAACGAGAGAGGATGCTCACGATACGGGTTACTGATGATGAACATGCCCGGTTACTGGAACGTTGTGAGGGTAAACAACTGGCGGTATGGATGCGGCGGGACCAGAGAAAAATCACTCAGGGTCAATGCCAGCGCTTCGTTAATACAGATGTAGGTGTTCCACAGGGTAGCCAGCAGCATCCTGCGATGCAGATCCGGAACATAATGGTGCAGGGCGCTGACTTCCGCGTTTCCAGACTTTACGAAACACGGAAACCGAAGACCATTCATGTTGTTGCTCAGGTCGCAGACGTTTTGCAGCAGCAGTCGCTTCACGTTCGCTCGCGTATCGGTGATTCATTCTGCTAACCAGTAAGGCAACCCCGCCAGCCTAGCCGGGTCCTCAACGACAGGAGCACGATCATGCGCACCCGTGGGGCCGCCATGCCGGCGATAATGGCCTGCTTCTCGCCGAAACGTTTGGTGGCGGGACCAGTGACGAAGGCTTGAGCGAGGGCGTGCAAGATTCCGAATACCGCAAGCGACAGGCCGATCATCGTCGCGCTCCAGCGAAAGCGGTCCTCGCCGAAAATGACCCAGAGCGCTGCCGGCACCTGTCCTACGAGTTGCATGATAAAGAAGACAGTCATAAGTGCGGCGACGATAGTCATGCCCCGCGCCCACCGGAAGGAGCTGACTGGGTTGAAGGCTCTCAAGGGCATCGGTCGAGATCCCGGTGCCTAATGAGTGAGCTAACTTACATTAATTGCGTTGCGCTCACTGCCCGCTTTCCAGTCGGGAAACCTGTCGTGCCAGCTGCATTAATGAATCGGCCAACGCGCGGGGAGAGGCGGTTTGCGTATTGGGCGCCAGGGTGGTTTTTCTTTTCACCAGTGAGACGGGCAACAGCTGATTGCCCTTCACCGCCTGGCCCTGAGAGAGTTGCAGCAAGCGGTCCACGCTGGTTTGCCCCAGCAGGCGAAAATCCTGTTTGATGGTGGTTAACGGCGGGATATAACATGAGCTGTCTTCGGTATCGTCGTATCCCACTACCGAGATATCCGCACCAACGCGCAGCCCGGACTCGGTAATGGCGCGCATTGCGCCCAGCGCCATCTGATCGTTGGCAACCAGCATCGCAGTGGGAACGATGCCCTCATTCAGCATTTGCATGGTTTGTTGAAAACCGGACATGGCACTCCAGTCGCCTTCCCGTTCCGCTATCGGCTGAATTTGATTGCGAGTGAGATATTTATGCCAGCCAGCCAGACGCAGACGCGCCGAGACAGAACTTAATGGGCCCGCTAACAGCGCGATTTGCTGGTGACCCAATGCGACCAGATGCTCCACGCCCAGTCGCGTACCGTCTTCATGGGAGAAAATAATACTGTTGATGGGTGTCTGGTCAGAGACATCAAGAAATAACGCCGGAACATTAGTGCAGGCAGCTTCCACAGCAATGGCATCCTGGTCATCCAGCGGATAGTTAATGATCAGCCCACTGACGCGTTGCGCGAGAAGATTGTGCACCGCCGCTTTACAGGCTTCGACGCCGCTTCGTTCTACCATCGACACCACCACGCTGGCACCCAGTTGATCGGCGCGAGATTTAATCGCCGCGACAATTTGCGACGGCGCGTGCAGGGCCAGACTGGAGGTGGCAACGCCAATCAGCAACGACTGTTTGCCCGCCAGTTGTTGTGCCACGCGGTTGGGAATGTAATTCAGCTCCGCCATCGCCGCTTCCACTTTTTCCCGCGTTTTCGCAGAAACGTGGCTGGCCTGGTTCACCACGCGGGAAACGGTCTGATAAGAGACACCGGCATACTCTGCGACATCGTATAACGTTACTGGTTTCACATTCACCACCCTGAATTGACTCTCTTCCGGGCGCTATCATGCCATACCGCGAAAGGTTTTGCGCCATTCGATGGTGTCCGGGATCTCGACGCTCTCCCTTATGCGACTCCTGCATTAGGAAGCAGCCCAGTAGTAGGTTGAGGCCGTTGAGCACCGCCGCCGCAAGGAATGGTGCATGCAAGGAGATGGCGCCCAACAGTCCCCCGGCCACGGGGCCTGCCACCATACCCACGCCGAAACAAGCGCTCATGAGCCCGAAGTGGCGAGCCCGATCTTCCCCATCGGTGATGTCGGCGATATAGGCGCCAGCAACCGCACCTGTGGCGCCGGTGATGCCGGCCACGATGCGTCCGGCGTAGAGGATCGAGATCTCGATCCCGCGAAATTAATACGACTCACTATAGGGGAATTGTGAGCGGATAACAATTCCCTTCTAGCCATATTGGACTCGGACCTGTTTCACGTGGAACACTGAGCCTGGACTAGGTCTAGCATATTGGACTCGACTTTCACGTGGAACACTGAGCCTGGACTAGGTCTAGAACAGGTTCTTTTTTCTTTGTTTCACGTGGAACATTCTGATTAATGTACAGCTAGCCATATTGGACTCGGACCTGTTTCACGTGGAACACTGAGCCTGGACTAGGTCTAGAACAGGTTCTTTTTTCTTTGTTTCACGTGGAACATTCTGATTAATGTACAGCTAGAACAGGTTCTTTTTTCTTTGTTTCACGTGGAACATTCTGATTAATGTACAGCTAGAAATAATTTTGTTTAACTTTAAGAAGGAGATATACCATGTGTTTCACGTGGAACACATGGCTAAAGGCCTTGGAAAAGGGATTAATGCGTTATTTAATCAGGTAGATTTGTCTGAAGAGACAGTTGAAGAAATTAAAATTGCCGATTTACGCCCTAATCCTTATCAGCCAAGAAAACACTTTGATGACGAGGCATTAGCTGAACTAAAAGAATCTGTGCTGCAGCATGGCATTCTTCAGCCGCTTATCGTCAGAAAATCTTTAAAAGGCTATGATATTGTTGCGGGTGAACGGCGTTTTCGAGCGGCAAAGCTGGCAGGTTTAGATACAGTTCCGGCCATTGTCCGTGAATTATCAGAGGCGTTAATGAGGGAAATTGCTTTATTAGAAAACCTTCAGCGTGAAGATTTATCTCCGCTTGAAGAGGCTCAGGCATATGACTCCCTTTTGAAACACTTAGATCTCACACAAGAGCAGCTTGCCAAACGTCTTGGGAAAAGCAGACCGCATATTGCGAATCATTTAAGACTGCTGACACTGCCAGAAAATATTCAACAGCTTATTGCCGAAGGCACGCTTTCTATGGGACATGGACGCACGCTTCTTGGCTTAAAAAACAAAAATAAGCTTGAACCGCTGGTACAAAAAGTGATTGCGGAGCAGCTCAATGTTCGCCAACTTGAGCAGCTGATTCAGCAGTTGAATCAGAATGTTCCACGTGAAACAAAGAAAAAAGAACCTGTGAAAGATGCGGTTCTAAAAGAACGGGAATCCTATCTCCAAAATTATTTTGGAACAACAGTTAATATTAAAAGACAGAAGAAAAAAGGCAAAATCGAAATTGAATTTTTCTCTAATGAAGACCTTGACCGGATTTTAGAGCTTTTGTCTGAACGAGAATCATAAGGATCCGAATTCGAGCTCCGTCGACAAGCTTCCGGCCGCAGTCGAGCACCACCACCACCACCACTGAGATCCGGCTGCTAACAAAGCCCGAAAGGAAGCTGAGTTGGCTGCTGCCACCGCTGAGCAATAACTAGCATAACCCCTTGGGGCCTCTAAACGGGTCTTGAGGGGTTTTTTGCTGAAAGGAGGAACTATATCCGGATTGGCGAATGGGACGCGCCCTGTAGCGGCGCATTAAGCGCGGCGGGTGTGGTGGTTACGCGCAGCGTGACCGCTACACTTGCCAGCGCCCTAGCGCCCGCTCCTTTCGCTTTCTTCCCTTCCTTTCTCGCCACGTTCGCCGGCTTTCCCCGTCAAGCTCTAAATCGGGGGCTCCCTTTAGGGTTCCGATTTAGTGCTTTACGGCACCTCGACCCCAAAAAACTTGATTAGGGTGATGGTTCACGTAGTGGGCCATCGCCCTGATAGACGGTTTTTCGCCCTTTGACGTTGGAGTCCACGTTCTTTAATAGTGGACTCTTGTTCCAAACTGGAACAACACTCAACCCTATCTCGGTCTATTCTTTTGATTTATAAGGGATTTTGCCGATTTCGGCCTATTGGTTAAAAAATGAGCTGATTTAACAAAAATTTAACGCGAATTTTAACAAAATATTAACGTTTACAATTTCAGGTGGCACTTTTCGGGGAAATGTGCGCGGAACCCCTATTTGTTTATTTTTCTAAATACATTCAAATATGTATCCGCTCATGAATTAATTCTTAGAAAAACTCATCGAGCATCAAATGAAACTGCAATTTATTCATATCAGGATTATCAATACCATATTTTTGAAAAAGCCGTTTCTGTAATGAAGGAGAAAACTCACCGAGGCAGTTCCATAGGATGGCAAGATCCTGGTATCGGTCTGCGATTCCGACTCGTCCAACATCAATACAACCTATTAATTTCCCCTCGTCAAAAATAAGGTTATCAAGTGAGAAATCACCATGAGTGACGACTGAATCCGGTGAGAATGGCAAAAGTTTATGCATTTCTTTCCAGACTTGTTCAACAGGCCAGCCATTACGCTCGTCATCAAAATCACTCGCATCAACCAAACCGTTATTCATTCGTGATTGCGCCTGAGCGAGACGAAATACGCGATCGCTGTTAAAAGGACAATTACAAACAGGAATCGAATGCAACCGGCGCAGGAACACTGCCAGCGCATCAACAATATTTTCACCTGAATCAGGATATTCTTCTAATACCTGGAATGCTGTTTTCCCGGGGATCGCAGTGGTGAGTAACCATGCATCATCAGGAGTACGGATAAAATGCTTGATGGTCGGAAGAGGCATAAATTCCGTCAGCCAGTTTAGTCTGACCATCTCATCTGTAACATCATTGGCAACGCTACCTTTGCCATGTTTCAGAAACAACTCTGGCGCATCGGGCTTCCCATACAATCGATAGATTGTCGCACCTGATTGCCCGACATTATCGCGAGCCCATTTATACCCATATAAATCAGCATCCATGTTGGAATTTAATCGCGGCCTAGAGCAAGACGTTTCCCGTTGAATATGGCTCATAACACCCCTTGTATTACTGTTTATGTAAGCAGACAGTTTTATTGTTCATGACCAAAATCCCTTAACGTGAGTTTTCGTTCCACTGAGCGTCAGACCCCGTAGAAAAGATCAAAGGATCTTCTTGAGATCCTTTTTTTCTGCGCGTAATCTGCTGCTTGCAAACAAAAAAACCACCGCTACCAGCGGTGGTTTGTTTGCCGGATCAAGAGCTACCAACTCTTTTTCCGAAGGTAACTGGCTTCAGCAGAGCGCAGATACCAAATACTGTCCTTCTAGTGTAGCCGTAGTTAGGCCACCACTTCAAGAACTCTGTAGCACCGCCTACATACCTCGCTCTGCTAATCCTGTTACCAGTGGCTGCTGCCAGTGGCGATAAGTCGTGTCTTACCGGGTTGGACTCAAGACGATAGTTACCGGATAAGGCGCAGCGGTCGGGCTGAACGGGGGGTTCGTGCACACAGCCCAGCTTGGAGCGAACGACCTACACCGAACTGAGATACCTACAGCGTGAGCTATGAGAAAGCGCCACGCTTCCCGAAGGGAGAAAGGCGGACAGGTATCCGGTAAGCGGCAGGGTCGGAACAGGAGAGCGCACGAGGGAGCTTCCAGGGGGAAACGCCTGGTATCTTTATAGTCCTGTCGGGTTTCGCCACCTCTGACTTGAGCGTCGATTTTTGTGATGCTCGTCAGGGGGGCGGAGCCTATGGAAAAACGCCAGCAACGCGGCCTTTTTACGGTTCCTGGCCTTTTGCTGGCCTTTTGCTCACATGTTCTTTCCTGCGTTATCCCCTGATTCTGTGGATAACCGTATTACCGCCTTTGAGTGAGCTGATACCGCTCGCCGCAGCCGAACGACCGAGCGCAGCGAGTCAGTGAGCGAGGAAGCGGAAGAGCGCCTGATGCGGTATTTTCTCCTTACGCATCTGTGCGGTATTTCACACCGCATATATGGTGCACTCTCAGTACAATCTGC

*MT EcoRI 7x parS DNA (6316 bp)*

GGCCGCTACCGATACTGCTGACCCAGCCACGGAACACATCGACCGTGCCGTTCGGGAAGCGGATTTTATAGGCACGGGTATCGCCTTCATTAAACCACGCCAGCAGCGCCTGCTGCCCCTGCTCTCCGGGCATCCACGCCAGCGTGAAGCTGGTATCTCCGGCAGATTTCTGCCCCTGCCCGGTCGCAGTCCAGTCTGCATCTTCATCATCGAGATAGCTGTCGTCATAGGACTCAGCGGTCAGTTCGCCGGGCGTCAGGTCTTTAACTTTTGCCAGACGCGACCAGTCAACGTCTGAAAGCGGATTCGCGTAAGGGTCACCGCTCCCCTTATAAACCCACAGGGTGGTCCCGGCACCTTTCACCGGCATTGTAGGATTTGGTACAGGCATAGCGTCCTCACATTTCATAGGTAATGACATAAGTCAGATCGGCTGAACTCCACAAGCCCGCATCATCGTCGCGCCGGTAGTCATAGCCGCTGGCCACCATACTGGTGATCAAATCTGACAGTGCCGGGATATCGCTCATCACCGGATAAATCCGGGACTCCATCCACGCATCCAGCTCTGAATCCGGCACCTGAGCAGGCAGGAAAACTTCGATATGCAGCTCCGCCTGCCAGGTATCGCTGTCCAGCTCTTCGCCCGTGTATTCAGCGCCGGTGAGATAAACGGCAACTGCCGGAAAATCCGCCTCATCAAAAACAGCGGGGCGACCATCAAAAAACGTCGCCCCGGTGTCATGCTTCTCCAGTGCATCCAGTACGGCTGCACGGAGTTCAGTATGTTTCATCGCTTTATTACCATCCTCAGTTGATGCTGCAGCGCATAGCCCAGCTCTTTCGGAAGACGTTCACGCCGTATCCGCTCAATATTTTGTTTAAACGCCGTGGTCAGCGGCACCGCCATCGGGATTTTCACCACATCAATGGGGTAACGGTTTTTCCCAGCCACACGCTGCATGACATGCCACCGGCCATTTTTCAGTTGCTGAATAAACGCGCCGGGAATACGACGGTTACCCACCACAAGCACGCTGCCGCCACCTTTCAGGGATGAACGCTGCCCCTTTTTACGACGCCTGCGGCGCGAAAGGACAACCCGCGCATTACCCAGCTTGATTACGGGCAAATCCCCCCGGTTAACTTTGATTCTGGCCTGCGGATTTTTGACCGTGGCCCTTTTCAGCCTGGCCCTTTCCTTTACCAGTTTCCGGCGTACCTTTGTCTCACGGGCAACCTGTGACGCCGACTGCGATATCGCGGATGAAGCAACGCGGTTAATGGCCATTGCGGCGGCACCAGGCACCGCCGTTTTGCTGATACGGCTGAGGTTTTCAACGGCCTGCTCAAGACCTTTTATGGCCATACATCCCCCTTTCAGCGGCGACGGTTAACGGCAGGCGGTACGCCCCGTCCAAGCCAGAGATGACAACTTCCGCCATCATCCGGCGAAACCCGATCTACCCAGAAATTTTCCTCACCGATGGTCAGCGTGTCTCCACGCCGCAGCTGCCGCACCTCATCAGTCCGGACAAACAGGGACGGGCTGGAGCCTTCAACGCGCACGCCCTGTCCGGCATAGCTGATATTTTCAGGGTCATCAAAAACACCACGTATCACCGCACCTGACTGCTCACCGGATGTAATGGTGGCTGACGTTCCCATGTACCCGCGTATCGTTTCATCGGCGCGGGCAATGGCAGCATCGAACAGGTTATCGAAATCAGCCACAGCGCCTCCCGTTATTGCATTCTGGCCAGGCCGCGCTCTGTCATTTCGGCTGCCACACCGGCAGAGACACGAAACGCCGTTCCCGGCAGCACAAATGCCACAGGTTCATCCCGCGTGGCGTGAAGTGCATCAGTATGCAGCTTCACCAGTGCCACGACCGTGACCAGTTCAGACGTATCCAGAATCACGGTATCCGGCTGCGCTGATCCCACCTCATTTTCATGTCCGGTCAGCACATTTTCCCGGCTGAGAGGGGTGTCCTGACCGGCAGTTTCATCCGTGTCATCAAGCTCCTCTTTCAGCTCTGCCACACGGAGCGCCAGTTCTTCTTTCGTCCCCGTCAGGCTGACATCACGGTTCAGTTGTTCACCCAGCGAGCGGAGACGGGCAATCAGTTCATCTTTCGTCATGGACTCCTCCACAGAGAAACAATGGCCCCGAAGGGCCATGATTACGCCAGTTGTACGGACACGAACTCATCAGGGTCAGCCAGCAGCATCAGCGGTGCTGACTGAATCATGGTGAACTCACGCGCCGGATCGCCGGTGGTCACCCAGTTTTTCGGGTAACGGGCAGAGGCGTTAATGCCTTCGCGCTGTGCGTCCGCATCCTGAATGCAGCCATAGGTGCGCAGACCGCGTGCCTGAGTGTTCCCCAGCACCATCGTGTTGTCCGGCAGGAAGTTCTTTTTGACGCCGTTTTCCACGTACTGTCCGGAATACACGACGATGGCCACATCGCCATACATCCCCTTATAGGACACCGCTTTGCCCAGGTCTTTCACCGCTGTCTCCAGCTCGGAATTAGAGCCACGACGGGTATCCAGCTTCTCCTTGACGGCTTTGAAGGAACGGAACAGCGCCCAGCCTTTCGGATCGAACACGATGATATTCACCACACCGCTGGCGTTCAGCGCGTAGGCTTCGATATCGTCGGTCGGGTCATACGTGGACTTGTCACGCTTGCTCCACTCCGTGCCGCCGGACTGCGTGATGTTATTCTCCTCACTGCGGCCCATATCCACCTCAACCGGATCGAAGGCTTCACCGGTCATGGTGTATTTGCCCTTAAGCACGGCAGAAACTGCCTGCATCTCTTCGACCTGAGCAATGGCCAGCTCTTCGTCACGCATGTTCTGCATGATGATGCGACGGCGGCGGTAAGCCGGGTCCGCCAGATTCTGCGGATCTTCATCCGGCAGGCGACGCAGGGTCATCTGCGGATTCACTTCATGCTTCGGCTTGACATATCCCGGCGTAAATTCAGAGGTGGAGCCGCCACGGGAACGGATAACCTCACCGGAAACAATCGGCGAAACGTACAGCGCCATGTTTACCAGTCCCGGAATTTGTGAGAGATAGACTTTCTCCGTGGTGAAGGGATAGCTCTCACGGAAAAAGAGACGCAGAAACAGCGGATCAAACTTAAATTTCTGCTCATTTGCCGCCAGCAGTTGGGCGGTTGTGTACATCGACATAAAAAAATCCCGTAAAAAAAGCCGCACAGGCGGCCTTTAGTGATGAAGGGTAAAGTTAAACGATGCTGATTGCCGTTCCGGCAAACGCGGTCCGTTTTTTCGTCTCGTCGCTGGCAGCCTCCGGCCAGAGCACATCCTCATAACGGAACGTGCCGGACTTGTAGAACGTCAGCGTGGTGCTGGTCTGGTCAGCAGCAACCGCAAGAATGCCAACGGCAGCACCGTCGGTGGTGCCATCCCACGCAACCAGCTTACGGCTGGAGGTGTCCAGCATCAGCGGGGTCATTGCAGGCGCTTTCGCACTCAATCCGCCGGGCGCGGTTGCGGTATGAGCCGGGTCACTGTTGCCCTGCGGCTGGTAATGGGTAAAGGTTTCTTTGCTCGTCATAAACATCCCTTACACTGGTGTGTTCAGCAAATCGTTAACGGCATCAGATGCCGGGTTACCTGCAGCCAGCGGTGCCGGTGCCCCCTGCATCAGACGATCCAGCGCAGTGTCACTGCGCGCCTGTGCACTCTGTGGTGCTGCGGCCAGAATGCGGCGGGCCGTTTTCACGGTCATACCGGGGGTTTCTGCCAGCACGCGTGCCTGTTCTTCGCGTCCGTGAGCCTCCTCACAGTTGAGAATTCGTCGTAGAATTCAACGTGGAATTCCTATCGGAATTCTCGGATGAATTCGGATCCTTATGATTCTCGTTCAGACAAAAGCTCTAAAATCCGGTCAAGGTCTTCATTAGAGAAAAATTCAATTTCGATTTTGCCTTTTTTCTTCTGTCTTTTAATATTAACTGTTGTTCCAAAATAATTTTGGAGATAGGATTCCCGTTCTTTTAGAACCGCATCTTTCACAGGTTCTTTTTTCTTTGTTTCACGTGGAACATTCTGATTCAACTGCTGAATCAGCTGCTCAAGTTGGCGAACATTGAGCTGCTCCGCAATCACTTTTTGTACCAGCGGTTCAAGCTTATTTTTGTTTTTTAAGCCAAGAAGCGTGCGTCCATGTCCCATAGAAAGCGTGCCTTCGGCAATAAGCTGTTGAATATTTTCTGGCAGTGTCAGCAGTCTTAAATGATTCGCAATATGCGGTCTGCTTTTCCCAAGACGTTTGGCAAGCTGCTCTTGTGTGAGATCTAAGTGTTTCAAAAGGGAGTCATATGCCTGAGCCTCTTCAAGCGGAGATAAATCTTCACGCTGAAGGTTTTCTAATAAAGCAATTTCCCTCATTAACGCCTCTGATAATTCACGGACAATGGCCGGAACTGTATCTAAACCTGCCAGCTTTGCCGCTCGAAAACGCCGTTCACCCGCAACAATATCATAGCCTTTTAAAGATTTTCTGACGATAAGCGGCTGAAGAATGCCATGCTGCAGCACAGATTCTTTTAGTTCAGCTAATGCCTCGTCATCAAAGTGTTTTCTTGGCTGATAAGGATTAGGGCGTAAATCGGCAATTTTAATTTCTTCAACTGTCTCTTCAGACAAATCTACCTGATTAAATAACGCATTAATCCCTTTTCCAAGGCCTTTAGCCATGTGTTCCACGTGAAACACATGGTATATCTCCTTCTTAAAGTTAAACAAAATTATTTCTAGCTGTACATTAATCAGAATGTTCCACGTGAAACAAAGAAAAAAGAACCTGTTCTAGCTGTACATTAATCAGAATGTTCCACGTGAAACAAAGAAAAAAGAACCTGTTCTAGACCTAGTCCAGGCTCAGTGTTCCACGTGAAACAGGTCCGAGTCCAATATGGCTAGCTGTACATTAATCAGAATGTTCCACGTGAAACAAAGAAAAAAGAACCTGTTCTAGACCTAGTCCAGGCTCAGTGTTCCACGTGAAAGTCGAGTCCAATATGCTAGACCTAGTCCAGGCTCAGTGTTCCACGTGAAACAGGTCCGAGTCCAATATGGCTAGAAGGGAATTGTTATCCGCTCACAATTCCCCTATAGTGAGTCGTATTAATTTCGCGGGATCGAGATCTCGATCCTCTACGCCGGACGCATCGTGGCCGGCATCACCGGCGCCACAGGTGCGGTTGCTGGCGCCTATATCGCCGACATCACCGATGGGGAAGATCGGGCTCGCCACTTCGGGCTCATGAGCGCTTGTTTCGGCGTGGGTATGGTGGCAGGCCCCGTGGCCGGGGGACTGTTGGGCGCCATCTCCTTGCATGCACCATTCCTTGCGGCGGCGGTGCTCAACGGCCTCAACCTACTACTGGGCTGCTTCCTAATGCAGGAGTCGCATAAGGGAGAGCGTCGAGATCCCGGACACCATCGAATGGCGCAAAACCTTTCGCGGTATGGCATGATAGCGCCCGGAAGAGAGTCAATTCAGGGTGGTGAATGTGAAACCAGTAACGTTATACGATGTCGCAGAGTATGCCGGTGTCTCTTATCAGACCGTTTCCCGCGTGGTGAACCAGGCCAGCCACGTTTCTGCGAAAACGCGGGAAAAAGTGGAAGCGGCGATGGCGGAGCTGAATTACATTCCCAACCGCGTGGCACAACAACTGGCGGGCAAACAGTCGTTGCTGATTGGCGTTGCCACCTCCAGTCTGGCCCTGCACGCGCCGTCGCAAATTGTCGCGGCGATTAAATCTCGCGCCGATCAACTGGGTGCCAGCGTGGTGGTGTCGATGGTAGAACGAAGCGGCGTCGAAGCCTGTAAAGCGGCGGTGCACAATCTTCTCGCGCAACGCGTCAGTGGGCTGATCATTAACTATCCGCTGGATGACCAGGATGCCATTGCTGTGGAAGCTGCCTGCACTAATGTTCCGGCGTTATTTCTTGATGTCTCTGACCAGACACCCATCAACAGTATTATTTTCTCCCATGAAGACGGTACGCGACTGGGCGTGGAGCATCTGGTCGCATTGGGTCACCAGCAAATCGCGCTGTTAGCGGGCCCATTAAGTTCTGTCTCGGCGCGTCTGCGTCTGGCTGGCTGGCATAAATATCTCACTCGCAATCAAATTCAGCCGATAGCGGAACGGGAAGGCGACTGGAGTGCCATGTCCGGTTTTCAACAAACCATGCAAATGCTGAATGAGGGCATCGTTCCCACTGCGATGCTGGTTGCCAACGATCAGATGGCGCTGC

*MT 13x parS DNA (6940 bp)*

GGCCGCCTGCATGTGTCAGAGGTTTTCACCGTCATCACCGAAACGCGCGAGGCAGCTGCGGTAAAGCTCATCAGCGTGGTCGTGAAGCGATTCACAGATGTCTGCCTGTTCATCCGCGTCCAGCTCGTTGAGTTTCTCCAGAAGCGTTAATGTCTGGCTTCTGATAAAGCGGGCCATGTTAAGGGCGGTTTTTTCCTGTTTGGTCACTGATGCCTCCGTGTAAGGGGGATTTCTGTTCATGGGGGTAATGATACCGATGAAACGAGAGAGGATGCTCACGATACGGGTTACTGATGATGAACATGCCCGGTTACTGGAACGTTGTGAGGGTAAACAACTGGCGGTATGGATGCGGCGGGACCAGAGAAAAATCACTCAGGGTCAATGCCAGCGCTTCGTTAATACAGATGTAGGTGTTCCACAGGGTAGCCAGCAGCATCCTGCGATGCAGATCCGGAACATAATGGTGCAGGGCGCTGACTTCCGCGTTTCCAGACTTTACGAAACACGGAAACCGAAGACCATTCATGTTGTTGCTCAGGTCGCAGACGTTTTGCAGCAGCAGTCGCTTCACGTTCGCTCGCGTATCGGTGATTCATTCTGCTAACCAGTAAGGCAACCCCGCCAGCCTAGCCGGGTCCTCAACGACAGGAGCACGATCATGCGCACCCGTGGGGCCGCCATGCCGGCGATAATGGCCTGCTTCTCGCCGAAACGTTTGGTGGCGGGACCAGTGACGAAGGCTTGAGCGAGGGCGTGCAAGATTCCGAATACCGCAAGCGACAGGCCGATCATCGTCGCGCTCCAGCGAAAGCGGTCCTCGCCGAAAATGACCCAGAGCGCTGCCGGCACCTGTCCTACGAGTTGCATGATAAAGAAGACAGTCATAAGTGCGGCGACGATAGTCATGCCCCGCGCCCACCGGAAGGAGCTGACTGGGTTGAAGGCTCTCAAGGGCATCGGTCGAGATCCCGGTGCCTAATGAGTGAGCTAACTTACATTAATTGCGTTGCGCTCACTGCCCGCTTTCCAGTCGGGAAACCTGTCGTGCCAGCTGCATTAATGAATCGGCCAACGCGCGGGGAGAGGCGGTTTGCGTATTGGGCGCCAGGGTGGTTTTTCTTTTCACCAGTGAGACGGGCAACAGCTGATTGCCCTTCACCGCCTGGCCCTGAGAGAGTTGCAGCAAGCGGTCCACGCTGGTTTGCCCCAGCAGGCGAAAATCCTGTTTGATGGTGGTTAACGGCGGGATATAACATGAGCTGTCTTCGGTATCGTCGTATCCCACTACCGAGATATCCGCACCAACGCGCAGCCCGGACTCGGTAATGGCGCGCATTGCGCCCAGCGCCATCTGATCGTTGGCAACCAGCATCGCAGTGGGAACGATGCCCTCATTCAGCATTTGCATGGTTTGTTGAAAACCGGACATGGCACTCCAGTCGCCTTCCCGTTCCGCTATCGGCTGAATTTGATTGCGAGTGAGATATTTATGCCAGCCAGCCAGACGCAGACGCGCCGAGACAGAACTTAATGGGCCCGCTAACAGCGCGATTTGCTGGTGACCCAATGCGACCAGATGCTCCACGCCCAGTCGCGTACCGTCTTCATGGGAGAAAATAATACTGTTGATGGGTGTCTGGTCAGAGACATCAAGAAATAACGCCGGAACATTAGTGCAGGCAGCTTCCACAGCAATGGCATCCTGGTCATCCAGCGGATAGTTAATGATCAGCCCACTGACGCGTTGCGCGAGAAGATTGTGCACCGCCGCTTTACAGGCTTCGACGCCGCTTCGTTCTACCATCGACACCACCACGCTGGCACCCAGTTGATCGGCGCGAGATTTAATCGCCGCGACAATTTGCGACGGCGCGTGCAGGGCCAGACTGGAGGTGGCAACGCCAATCAGCAACGACTGTTTGCCCGCCAGTTGTTGTGCCACGCGGTTGGGAATGTAATTCAGCTCCGCCATCGCCGCTTCCACTTTTTCCCGCGTTTTCGCAGAAACGTGGCTGGCCTGGTTCACCACGCGGGAAACGGTCTGATAAGAGACACCGGCATACTCTGCGACATCGTATAACGTTACTGGTTTCACATTCACCACCCTGAATTGACTCTCTTCCGGGCGCTATCATGCCATACCGCGAAAGGTTTTGCGCCATTCGATGGTGTCCGGGATCTCGACGCTCTCCCTTATGCGACTCCTGCATTAGGAAGCAGCCCAGTAGTAGGTTGAGGCCGTTGAGCACCGCCGCCGCAAGGAATGGTGCATGCAAGGAGATGGCGCCCAACAGTCCCCCGGCCACGGGGCCTGCCACCATACCCACGCCGAAACAAGCGCTCATGAGCCCGAAGTGGCGAGCCCGATCTTCCCCATCGGTGATGTCGGCGATATAGGCGCCAGCAACCGCACCTGTGGCGCCGGTGATGCCGGCCACGATGCGTCCGGCGTAGAGGATCGAGATCTCGATCCCGCGAAATTAATACGACTCACTATAGGGGAATTGTGAGCGGATAACAATTCCCTTCTAGCCATATTGGACTCGGACCTGTTTCACGTGGAACACTGAGCCTGGACTAGGTCTAGCATATTGGACTCGACTTTCACGTGGAACACTGAGCCTGGACTAGGTCTAGAACAGGTTCTTTTTTCTTTGTTTCACGTGGAACATTCTGATTAATGTACAGCTAGCCATATTGGACTCGGACCTGTTTCACGTGGAACACTGAGCCTGGACTAGGTCTAGAACAGGTTCTTTTTTCTTTGTTTCACGTGGAACATTCTGATTAATGTACAGCTAGAACAGGTTCTTTTTTCTTTGTTTCACGTGGAACATTCTGATTAATGTACAGCTAGAAATAATTTTGTTTAACTTTAAGAAGGAGATATACCATGTGTTTCACGTGGAACACATGGCTAAAGGCCTTGGAAAAGGGATTAATGCGTTATTTAATCAGGTAGATTTGTCTGAAGAGACAGTTGAAGAAATTAAAATTGCCGATTTACGCCCTAATCCTTATCAGCCAAGAAAACACTTTGATGACGAGGCATTAGCTGAACTAAAAGAATCTGTGCTGCAGCATGGCATTCTTCAGCCGCTTATCGTCAGAAAATCTTTAAAAGGCTATGATATTGTTGCGGGTGAACGGCGTTTTCGAGCGGCAAAGCTGGCAGGTTTAGATACAGTTCCGGCCATTGTCCGTGAATTATCAGAGGCGTTAATGAGGGAAATTGCTTTATTAGAAAACCTTCAGCGTGAAGATTTATCTCCGCTTGAAGAGGCTCAGGCATATGCCGAAACAAGCGCTCATGAGCCCGAAGTGGCGAGCCCGATCTTCCCCATCGGTGATGTCGGCGATATAGGCGCCAGCAACCGCACCTGTGGCGCCGGTGATGCCGGCCACGATGCGTCCGGCGTAGAGGATCGAGATCTCGATCCCGCGAAATTAATACGACTCACTATAGGGGAATTGTGAGCGGATAACAATTCCCTTCTAGCCATATTGGACTCGGACCTGTTTCACGTGGAACACTGAGCCTGGACTAGGTCTAGCATATTGGACTCGACTTTCACGTGGAACACTGAGCCTGGACTAGGTCTAGAACAGGTTCTTTTTTCTTTGTTTCACGTGGAACATTCTGATTAATGTACAGCTAGCCATATTGGACTCGGACCTGTTTCACGTGGAACACTGAGCCTGGACTAGGTCTAGAACAGGTTCTTTTTTCTTTGTTTCACGTGGAACATTCTGATTAATGTACAGCTAGAACAGGTTCTTTTTTCTTTGTTTCACGTGGAACATTCTGATTAATGTACAGCTAGAAATAATTTTGTTTAACTTTAAGAAGGAGATATACCATGTGTTTCACGTGGAACACATGGCTAAAGGCCTTGGAAAAGGGATTAATGCGCATATGACTCCCTTTTGAAACACTTAGATCTCACACAAGAGCAGCTTGCCAAACGTCTTGGGAAAAGCAGACCGCATATTGCGAATCATTTAAGACTGCTGACACTGCCAGAAAATATTCAACAGCTTATTGCCGAAGGCACGCTTTCTATGGGACATGGACGCACGCTTCTTGGCTTAAAAAACAAAAATAAGCTTGAACCGCTGGTACAAAAAGTGATTGCGGAGCAGCTCAATGTTCGCCAACTTGAGCAGCTGATTCAGCAGTTGAATCAGAATGTTCCACGTGAAACAAAGAAAAAAGAACCTGTGAAAGATGCGGTTCTAAAAGAACGGGAATCCTATCTCCAAAATTATTTTGGAACAACAGTTAATATTAAAAGACAGAAGAAAAAAGGCAAAATCGAAATTGAATTTTTCTCTAATGAAGACCTTGACCGGATTTTAGAGCTTTTGTCTGAACGAGAATCATAAGGATCCGAATTCGAGCTCCGTCGACAAGCTTCCGGCCGCAGTCGAGCACCACCACCACCACCACTGAGATCCGGCTGCTAACAAAGCCCGAAAGGAAGCTGAGTTGGCTGCTGCCACCGCTGAGCAATAACTAGCATAACCCCTTGGGGCCTCTAAACGGGTCTTGAGGGGTTTTTTGCTGAAAGGAGGAACTATATCCGGATTGGCGAATGGGACGCGCCCTGTAGCGGCGCATTAAGCGCGGCGGGTGTGGTGGTTACGCGCAGCGTGACCGCTACACTTGCCAGCGCCCTAGCGCCCGCTCCTTTCGCTTTCTTCCCTTCCTTTCTCGCCACGTTCGCCGGCTTTCCCCGTCAAGCTCTAAATCGGGGGCTCCCTTTAGGGTTCCGATTTAGTGCTTTACGGCACCTCGACCCCAAAAAACTTGATTAGGGTGATGGTTCACGTAGTGGGCCATCGCCCTGATAGACGGTTTTTCGCCCTTTGACGTTGGAGTCCACGTTCTTTAATAGTGGACTCTTGTTCCAAACTGGAACAACACTCAACCCTATCTCGGTCTATTCTTTTGATTTATAAGGGATTTTGCCGATTTCGGCCTATTGGTTAAAAAATGAGCTGATTTAACAAAAATTTAACGCGAATTTTAACAAAATATTAACGTTTACAATTTCAGGTGGCACTTTTCGGGGAAATGTGCGCGGAACCCCTATTTGTTTATTTTTCTAAATACATTCAAATATGTATCCGCTCATGAATTAATTCTTAGAAAAACTCATCGAGCATCAAATGAAACTGCAATTTATTCATATCAGGATTATCAATACCATATTTTTGAAAAAGCCGTTTCTGTAATGAAGGAGAAAACTCACCGAGGCAGTTCCATAGGATGGCAAGATCCTGGTATCGGTCTGCGATTCCGACTCGTCCAACATCAATACAACCTATTAATTTCCCCTCGTCAAAAATAAGGTTATCAAGTGAGAAATCACCATGAGTGACGACTGAATCCGGTGAGAATGGCAAAAGTTTATGCATTTCTTTCCAGACTTGTTCAACAGGCCAGCCATTACGCTCGTCATCAAAATCACTCGCATCAACCAAACCGTTATTCATTCGTGATTGCGCCTGAGCGAGACGAAATACGCGATCGCTGTTAAAAGGACAATTACAAACAGGAATCGAATGCAACCGGCGCAGGAACACTGCCAGCGCATCAACAATATTTTCACCTGAATCAGGATATTCTTCTAATACCTGGAATGCTGTTTTCCCGGGGATCGCAGTGGTGAGTAACCATGCATCATCAGGAGTACGGATAAAATGCTTGATGGTCGGAAGAGGCATAAATTCCGTCAGCCAGTTTAGTCTGACCATCTCATCTGTAACATCATTGGCAACGCTACCTTTGCCATGTTTCAGAAACAACTCTGGCGCATCGGGCTTCCCATACAATCGATAGATTGTCGCACCTGATTGCCCGACATTATCGCGAGCCCATTTATACCCATATAAATCAGCATCCATGTTGGAATTTAATCGCGGCCTAGAGCAAGACGTTTCCCGTTGAATATGGCTCATAACACCCCTTGTATTACTGTTTATGTAAGCAGACAGTTTTATTGTTCATGACCAAAATCCCTTAACGTGAGTTTTCGTTCCACTGAGCGTCAGACCCCGTAGAAAAGATCAAAGGATCTTCTTGAGATCCTTTTTTTCTGCGCGTAATCTGCTGCTTGCAAACAAAAAAACCACCGCTACCAGCGGTGGTTTGTTTGCCGGATCAAGAGCTACCAACTCTTTTTCCGAAGGTAACTGGCTTCAGCAGAGCGCAGATACCAAATACTGTCCTTCTAGTGTAGCCGTAGTTAGGCCACCACTTCAAGAACTCTGTAGCACCGCCTACATACCTCGCTCTGCTAATCCTGTTACCAGTGGCTGCTGCCAGTGGCGATAAGTCGTGTCTTACCGGGTTGGACTCAAGACGATAGTTACCGGATAAGGCGCAGCGGTCGGGCTGAACGGGGGGTTCGTGCACACAGCCCAGCTTGGAGCGAACGACCTACACCGAACTGAGATACCTACAGCGTGAGCTATGAGAAAGCGCCACGCTTCCCGAAGGGAGAAAGGCGGACAGGTATCCGGTAAGCGGCAGGGTCGGAACAGGAGAGCGCACGAGGGAGCTTCCAGGGGGAAACGCCTGGTATCTTTATAGTCCTGTCGGGTTTCGCCACCTCTGACTTGAGCGTCGATTTTTGTGATGCTCGTCAGGGGGGCGGAGCCTATGGAAAAACGCCAGCAACGCGGCCTTTTTACGGTTCCTGGCCTTTTGCTGGCCTTTTGCTCACATGTTCTTTCCTGCGTTATCCCCTGATTCTGTGGATAACCGTATTACCGCCTTTGAGTGAGCTGATACCGCTCGCCGCAGCCGAACGACCGAGCGCAGCGAGTCAGTGAGCGAGGAAGCGGAAGAGCGCCTGATGCGGTATTTTCTCCTTACGCATCTGTGCGGTATTTCACACCGCATATATGGTGCACTCTCAGTACAATCTGC

*MT 26x parS DNA (6348 bp)*

GGCCGCCTGCATGTGTCAGAGGTTTTCACCGTCATCACCGAAACGCGCGAGGCAGCTGCGGTAAAGCTCATCAGCGTGGTCGTGAAGCGATTCACAGATGTCTGCCTGTTCATCCGCGTCCAGCTCGTTGAGTTTCTCCAGAAGCGTTAATGTCTGGCTTCTGATAAAGCGGGCCATGTTAAGGGCGGTTTTTTCCTGTTTGGTCACTGATGCCTCCGTGTAAGGGGGATTTCTGTTCATGGGGGTAATGATACCGATGAAACGAGAGAGGATGCTCACGATACGGGTTACTGATGATGAACATGCCCGGTTACTGGAACGTTGTGAGGGTAAACAACTGGCGGTATGGATGCGGCGGGACCAGAGAAAAATCACTCAGGGTCAATGCCAGCGCTTCGTTAATACAGATGTAGGTGTTCCACAGGGTAGCCAGCAGCATCCTGCGATGCAGATCCGGAACATAATGGTGCAGGGCGCTGACTTCCGCGTTTCCAGACTTTACGAAACACGGAAACCGAAGACCATTCATGTTGTTGCTCAGGTCGCAGACGTTTTGCAGCAGCAGTCGCTTCACGTTCGCTCGCGTATCGGTGATTCATTCTGCTAACCAGTAAGGCAACCCCGCCAGCCTAGCCGGGTCCTCAACGACAGGAGCACGATCATGCGCACCCGTGGGGCCGCCATGCCGGCGCCTGCCACCATACCCACGCCGAAACAAGCGCTCATGAGCCCGAAGTGGCGAGCCCGATCTTCCCCATCGGTGATGTCGGCGATATAGGCGCCAGCAACCGCACCTGTGGCGCCGGTGATGCCGGCCACGATGCGTCCGGCGTAGAGGATCGAGATCTCGATCCCGCGAAATTAATACGACTCACTATAGGGGAATTGTGAGCGGATAACAATTCCCTTCTAGCCATATTGGACTCGGACCTGTTTCACGTGGAACACTGAGCCTGGACTAGGTCTAGCATATTGGACTCGACTTTCACGTGGAACACTGAGCCTGGACTAGGTCTAGAACAGGTTCTTTTTTCTTTGTTTCACGTGGAACATTCTGATTAATGTACAGCTAGCCATATTGGACTCGGACCTGTTTCACGTGGAACACTGAGCCTGGACTAGGTCTAGAACAGGTTCTTTTTTCTTTGTTTCACGTGGAACATTCTGATTAATGTACAGCTAGAACAGGTTCTTTTTTCTTTGTTTCACGTGGAACATTCTGATTAATGTACAGCTAGAAATAATTTTGTTTAACTTTAAGAAGGAGATATACCATGTGTTTCACGTGGAACACATGGCTAAAGGCCTTGGAAAAGGGATTAATGCGTTATTTAATCAGGTAGATTTGTCTGAAGAGACAGTTGAAGAAATTAAAATTGCCGATTTACGCCCTAATCCTTATCAGCCAAGAAAACACTTTGATGACGAGGCATTAGCTGAACTAAAAGAATCTGTGCTGCAGCATGGCATTCTTCAGCCGCTTATCGTCAGAAAATCTTTAAAAGGCTATGATATTGTTGCGGGTGAACGGCGTTTTCGAGCGGCAAAGCTGGCAGGTTTAGATACAGTTCCGGCCATTGTCCGTGAATTATCAGAGGCGTTAATGAGGGAAATTGCTTTATTAGAAAACCTTCAGCGTGAAGATTTATCTCCGCTTGAAGAGGCTCAGGCATATGCCGAAACAAGCGCTCATGAGCCCGAAGTGGCGAGCCCGATCTTCCCCATCGGTGATGTCGGCGATATAGGCGCCAGCAACCGCACCTGTGGCGCCGGTGATGCCGGCCACGATGCGTCCGGCGTAGAGGATCGAGATCTCGATCCCGCGAAATTAATACGACTCACTATAGGGGAATTGTGAGCGGATAACAATTCCCTTCTAGCCATATTGGACTCGGACCTGTTTCACGTGGAACACTGAGCCTGGACTAGGTCTAGCATATTGGACTCGACTTTCACGTGGAACACTGAGCCTGGACTAGGTCTAGAACAGGTTCTTTTTTCTTTGTTTCACGTGGAACATTCTGATTAATGTACAGCTAGCCATATTGGACTCGGACCTGTTTCACGTGGAACACTGAGCCTGGACTAGGTCTAGAACAGGTTCTTTTTTCTTTGTTTCACGTGGAACATTCTGATTAATGTACAGCTAGAACAGGTTCTTTTTTCTTTGTTTCACGTGGAACATTCTGATTAATGTACAGCTAGAAATAATTTTGTTTAACTTTAAGAAGGAGATATACCATGTGTTTCACGTGGAACACATGGCTAAAGGCCTTGGAAAAGGGATTAATGCGCATATGACTCCCTTTTGAAACACTTAGATCTCACACAAGAGCAGCTTGCCAAACGTCTTGGGAAAAGCAGACCGCATATTGCGAATCATTTAAGACTGCTGACACTGCCAGAAAATATTCAACAGCTTATTGCCGAAGGCACGCTTTCTATGGGACATGGACGCACGCTTCTTGGCTTAAAAAACAAAAATAAGCTTGAACCGCTGGTACAAAAAGTGATTGCGGAGCAGCTCAATGTTCGCCAACTTGAGCAGCTGATTCAGCAGTTGAATCAGAATGTTCCACGTGAAACAAAGAAAAAAGAACCTGTGAAAGATGCGGTTCGCCATGCCGGCGATAATGGCCTGCTTCTCGCCGAAACGTTTGGTGGCGGGACCAGTGACGAAGGCTTGAGCGAGGGCGTGCAAGATTCCGAATACCGCAAGCGACAGGCCGATCATCGTCGCGCTCCAGCGAAAGCGGTCCTCGCCGAAAATGACCCAGAGCGCTGCCGGCACCTGTCCTACGAGTTGCATGATAAAGAAGACAGTCATAAGTGCGGCGACGATAGTCATGCCCCGCGCCCACCGGAAGGAGCTGACTGGGTTGAAGGCTCTCAAGGGCATCGGTCGAGATCCCGGTGCCTAATGAGTGAGCTAACTTACATTAATTGCGTTGCGCTCACTGCCCGCTTTCCAGTCGGGAAACCTGTCGTGCCAGCTGCATTAATGAATCGGCCAACGCGCGGGGAGAGGCGGTTTGCGTATTGGGCGCCAGGGTGGTTTTTCTTTTCACCAGTGAGACGGGCAACAGCTGATTGCCCTTCACCGCCTGGCCCTGAGAGAGTTGCAGCAAGCGGTCCACGCTGGTTTGCCCCAGCAGGCGAAAATCCTGTTTGATGGTGGTTAACGGCGGGATATAACATGAGCTGTCTTCGGTATCGTCGTATCCCACTACCGAGATATCCGCACCAACGCGCAGCCCGGACTCGGTAATGGCGCGCATTGCGCCCAGCGCCATCTGATCGTTGGCAACCAGCATCGCAGTGGGAACGATGCCCTCATTCAGCATTTGCATGGTTTGTTGAAAACCGGACATGGCACTCCAGTCGCCTTCCCGTTCCGCTATCGGCTGAATTTGATTGCGAGTGAGATATTTATGCCAGCCAGCCAGACGCAGACGCGCCGAGACAGAACTTAATGGGCCCGCTAACAGCGCGATTTGCTGGTGACCCAATGCGACCAGATGCTCCACGCCCAGTCGCGTACCGTCTTCATGGGAGAAAATAATACTGTTGATGGGTGTCTGGTCAGAGACATCAAGAAATAACGCCGGAACATTAGTGCAGGCAGCTTCCACAGCAATGGCATCCTGGTCATCCAGCGGATAGTTAATGATCAGCCCACTGACGCGTTGCGCGAGAAGATTGTGCACCGCCGCTTTACAGGCTTCGACGCCGCTTCGTTCTACCATCGACACCACCACGCTGGCACCCAGTTGATCGGCGCGAGATTTAATCGCCGCGACAATTTGCGACGGCGCGTGCAGGGCCAGACTGGAGGTGGCAACGCCAATCAGCAACGACTGTTTGCCCGCCAGTTGTTGTGCCACGCGGTTGGGAATGTAATTCAGCTCCGCCATCGCCGCTTCCACTTTTTCCCGCGTTTTCGCAGAAACGTGGCTGGCCTGGTTCACCACGCGGGAAACGGTCTGATAAGAGACACCGGCATACTCTGCGACATCGTATAACGTTACTGGTTTCACATTCACCACCCTGAATTGACTCTCTTCCGGGCGCTATCATGCCATACCGCGAAAGGTTTTGCGCCATTCGATGGTGTCCGGGATCTCGACGCTCTCCCTTATGCGACTCCTGCATTAGGAAGCAGCCCAGTAGTAGGTTGAGGCCGTTGAGCACCGCCGCCGCAAGGAATGGTGCATGCAAGGAGATGGCGCCCAACAGTCCCCCGGCCACGGGGCCTGCCACCATACCCACGCCGAAACAAGCGCTCATGAGCCCGAAGTGGCGAGCCCGATCTTCCCCATCGGTGATGTCGGCGATATAGGCGCCAGCAACCGCACCTGTGGCGCCGGTGATGCCGGCCACGATGCGTCCGGCGTAGAGGATCGAGATCTCGATCCCGCGAAATTAATACGACTCACTATAGGGGAATTGTGAGCGGATAACAATTCCCTTCTAGCCATATTGGACTCGGACCTGTTTCACGTGGAACACTGAGCCTGGACTAGGTCTAGCATATTGGACTCGACTTTCACGTGGAACACTGAGCCTGGACTAGGTCTAGAACAGGTTCTTTTTTCTTTGTTTCACGTGGAACATTCTGATTAATGTACAGCTAGCCATATTGGACTCGGACCTGTTTCACGTGGAACACTGAGCCTGGACTAGGTCTAGAACAGGTTCTTTTTTCTTTGTTTCACGTGGAACATTCTGATTAATGTACAGCTAGAACAGGTTCTTTTTTCTTTGTTTCACGTGGAACATTCTGATTAATGTACAGCTAGAAATAATTTTGTTTAACTTTAAGAAGGAGATATACCATGTGTTTCACGTGGAACACATGGCTAAAGGCCTTGGAAAAGGGATTAATGCGTTATTTAATCAGGTAGATTTGTCTGAAGAGACAGTTGAAGAAATTAAAATTGCCGATTTACGCCCTAATCCTTATCAGCCAAGAAAACACTTTGATGACGAGGCATTAGCTGAACTAAAAGAATCTGTGCTGCAGCATGGCATTCTTCAGCCGCTTATCGTCAGAAAATCTTTAAAAGGCTATGATATTGTTGCGGGTGAACGGCGTTTTCGAGCGGCAAAGCTGGCAGGTTTAGATACAGTTCCGGCCATTGTCCGTGAATTATCAGAGGCGTTAATGAGGGAAATTGCTTTATTAGAAAACCTTCAGCGTGAAGATTTATCTCCGCTTGAAGAGGCTCAGGCATATGCCGAAACAAGCGCTCATGAGCCCGAAGTGGCGAGCCCGATCTTCCCCATCGGTGATGTCGGCGATATAGGCGCCAGCAACCGCACCTGTGGCGCCGGTGATGCCGGCCACGATGCGTCCGGCGTAGAGGATCGAGATCTCGATCCCGCGAAATTAATACGACTCACTATAGGGGAATTGTGAGCGGATAACAATTCCCTTCTAGCCATATTGGACTCGGACCTGTTTCACGTGGAACACTGAGCCTGGACTAGGTCTAGCATATTGGACTCGACTTTCACGTGGAACACTGAGCCTGGACTAGGTCTAGAACAGGTTCTTTTTTCTTTGTTTCACGTGGAACATTCTGATTAATGTACAGCTAGCCATATTGGACTCGGACCTGTTTCACGTGGAACACTGAGCCTGGACTAGGTCTAGAACAGGTTCTTTTTTCTTTGTTTCACGTGGAACATTCTGATTAATGTACAGCTAGAACAGGTTCTTTTTTCTTTGTTTCACGTGGAACATTCTGATTAATGTACAGCTAGAAATAATTTTGTTTAACTTTAAGAAGGAGATATACCATGTGTTTCACGTGGAACACATGGCTAAAGGCCTTGGAAAAGGGATTAATGCGCATATGACTCCCTTTTGAAACACTTAGATCTCACACAAGAGCAGCTTGCCAAACGTCTTGGGAAAAGCAGACCGCATATTGCGAATCATTTAAGACTGCTGACACTGCCAGAAAATATTCAACAGCTTATTGCCGAAGGCACGCTTTCTATGGGACATGGACGCACGCTTCTTGGCTTAAAAAACAAAAATAAGCTTGAACCGCTGGTACAAAAAGTGATTGCGGAGCAGCTCAATGTTCGCCAACTTGAGCAGCTGATTCAGCAGTTGAATCAGAATGTTCCACGTGAAACAAAGAAAAAAGAACCTGTGAAAGATGCGGTTCTAAAAGAACGGGAATCCTATCTCCAAAATTATTTTGGAACAACAGTTAATATTAAAAGACAGAAGAAAAAAGGCAAAATCGAAATTGAATTTTTCTCTAATGAAGACCTTGACCGGATTTTAGAGCTTTTGTCTGAACGAGAATCATAAGGATCCGAATTCGAGCTCCG

*C-trap 39x parS DNA (24974 bp)*

GGCCGCGGTGTGCTCCTTATTTATACATAACGAAAAACGCCTCGAGTGAAGCGTTATTGGTATGCGGTAAAACCGCACTCAGGCGGCCTTGATAGTCATATCATCTGAATCAAATATTCCTGATGTATCGATATCGGTAATTCTTATTCCTTCGCTACCATCCATTGGAGGCCATCCTTCCTGACCATTTCCATCATTCCAGTCGAACTCACACACAACACCATATGCATTTAAGTCGCTTGAAATTGCTATAAGCAGAGCATGTTGCGCCAGCATGATTAATACAGCATTTAATACAGAGCCGTGTTTATTGAGTCGGTATTCAGAGTCTGACCAGAAATTATTAATCTGGTGAAGTTTTTCCTCTGTCATTACGTCATGGTCGATTTCAATTTCTATTGATGCTTTCCAGTCGTAATCAATGATGTATTTTTTGATGTTTGACATCTGTTCATATCCTCACAGATAAAAAATCGCCCTCACACTGGAGGGCAAAGAAGATTTCCAATAATCAGAACAAGTCGGCTCCTGTTTAGTTACGAGCGACATTGCTCCGTGTATTCACTCGTTGGAATGAATACACAGTGCAGTGTTTATTCTGTTATTTATGCCAAAAATAAAGGCCACTATCAGGCAGCTTTGTTGTTCTGTTTACCAAGTTCTCTGGCAATCATTGCCGTCGTTCGTATTGCCCATTTATCGACATATTTCCCATCTTCCATTACAGGAAACATTTCTTCAGGCTTAACCATGCATTCCGATTGCAGCTTGCATCCATTGCATCGCTTGAATTGTCCACACCATTGATTTTTATCAATAGTCGTAGTCATACGGATAGTCCTGGTATTGTTCCATCACATCCTGAGGATGCTCTTCGAACTCTTCAAATTCTTCTTCCATATATCACCTTAAATAGTGGATTGCGGTAGTAAAGATTGTGCCTGTCTTTTAACCACATCAGGCTCGGTGGTTCTCGTGTACCCCTACAGCGAGAAATCGGATAAACTATTACAACCCCTACAGTTTGATGAGTATAGAAATGGATCCACTCGTTATTCTCGGACGAGTGTTCAGTAATGAACCTCTGGAGAGAACCATGTATATGATCGTTATCTGGGTTGGACTTCTGCTTTTAAGCCCAGATAACTGGCCTGAATATGTTAATGAGAGAATCGGTATTCCTCATGTGTGGCATGTTTTCGTCTTTGCTCTTGCATTTTCGCTAGCAATTAATGTGCATCGATTATCAGCTATTGCCAGCGCCAGATATAAGCGATTTAAGCTAAGAAAACGCATTAAGATGCAAAACGATAAAGTGCGATCAGTAATTCAAAACCTTACAGAAGAGCAATCTATGGTTTTGTGCGCAGCCCTTAATGAAGGCAGGAAGTATGTGGTTACATCAAAACAATTCCCATACATTAGTGAGTTGATTGAGCTTGGTGTGTTGAACAAAACTTTTTCCCGATGGAATGGAAAGCATATATTATTCCCTATTGAGGATATTTACTGGACTGAATTAGTTGCCAGCTATGATCCATATAATATTGAGATAAAGCCAAGGCCAATATCTAAGTAACTAGATAAGAGGAATCGATTTTCCCTTAATTTTCTGGCGTCCACTGCATGTTATGCCGCGTTCGCCAGGCTTGCTGTACCATGTGCGCTGATTCTTGCGCTCAATACGTTGCAGGTTGCTTTCAATCTGTTTGTGGTATTCAGCCAGCACTGTAAGGTCTATCGGATTTAGTGCGCTTTCTACTCGTGATTTCGGTTTGCGATTCAGCGAGAGAATAGGGCGGTTAACTGGTTTTGCGCTTACCCCAACCAACAGGGGATTTGCTGCTTTCCATTGAGCCTGTTTCTCTGCGCGACGTTCGCGGCGGCGTGTTTGTGCATCCATCTGGATTCTCCTGTCAGTTAGCTTTGGTGGTGTGTGGCAGTTGTAGTCCTGAACGAAAACCCCCCGCGATTGGCACATTGGCAGCTAATCCGGAATCGCACTTACGGCCAATGCTTCGTTTCGTATCACACACCCCAAAGCCTTCTGCTTTGAATGCTGCCCTTCTTCAGGGCTTAATTTTTAAGAGCGTCACCTTCATGGTGGTCAGTGCGTCCTGCTGATGTGCTCAGTATCACCGCCAGTGGTATTTATGTCAACACCGCCAGAGATAATTTATCACCGCAGATGGTTATCTGTATGTTTTTTATATGAATTTATTTTTTGCAGGGGGGCATTGTTTGGTAGGTGAGAGATCTGAATTGCTATGTTTAGTGAGTTGTATCTATTTATTTTTCAATAAATACAATTGGTTATGTGTTTTGGGGGCGATCGTGAGGCAAAGAAAACCCGGCGCTGAGGCCGGGTTATTCTTGTTCTCTGGTCAAATTATATAGTTGGAAAACAAGGATGCATATATGAATGAACGATGCAGAGGCAATGCCGATGGCGATAGTGGGTATCATGTAGCCGCTTATGCTGGAAAGAAGCAATAACCCGCAGAAAAACAAAGCTCCAAGCTCAACAAAACTAAGGGCATAGACAATAACTACCGATGTCATATACCCATACTCTCTAATCTTGGCCAGTCGGCGCGTTCTGCTTCCGATTAGAAACGTCAAGGCAGCAATCAGGATTGCAATCATGGTTCCTGCATATGATGACAATGTCGCCCCAAGACCATCTCTATGAGCTGAAAAAGAAACACCAGGAATGTAGTGGCGGAAAAGGAGATAGCAAATGCTTACGATAACGTAAGGAATTATTACTATGTAAACACCAGGCATGATTCTGTTCCGCATAATTACTCCTGATAATTAATCCTTAACTTTGCCCACCTGCCTTTTAAAACATTCCAGTATATCACTTTTCATTCTTGCGTAGCAATATGCCATCTCTTCAGCTATCTCAGCATTGGTGACCTTGTTCAGAGGCGCTGAGAGATGGCCTTTTTCTGATAGATAATGTTCTGTTAAAATATCTCCGGCCTCATCTTTTGCCCGCAGGCTAATGTCTGAAAATTGAGGTGACGGGTTAAAAATAATATCCTTGGCAACCTTTTTTATATCCCTTTTAAATTTTGGCTTAATGACTATATCCAATGAGTCAAAAAGCTCCCCTTCAATATCTGTTGCCCCTAAGACCTTTAATATATCGCCAAATACAGGTAGCTTGGCTTCTACCTTCACCGTTGTTCGGCCGATGAAATGCATATGCATAACATCGTCTTTGGTGGTTCCCCTCATCAGTGGCTCTATCTGAACGCGCTCTCCACTGCTTAATGACATTCCTTTCCCGATTAAAAAATCTGTCAGATCGGATGTGGTCGGCCCGAAAACAGTTCTGGCAAAACCAATGGTGTCGCCTTCAACAAACAAAAAAGATGGGAATCCCAATGATTCGTCATCTGCGAGGCTGTTCTTAATATCTTCAACTGAAGCTTTAGAGCGATTTATCTTCTGAACCAGACTCTTGTCATTTGTTTTGGTAAAGAGAAAAGTTTTTCCATCGATTTTATGAATATACAAATAATTGGAGCCAACCTGCAGGTGATGATTATCAGCCAGCAGAGAATTAAGGAAAACAGACAGGTTTATTGAGCGCTTATCTTTCCCTTTATTTTTGCTGCGGTAAGTCGCATAAAAACCATTCTTCATAATTCAATCCATTTACTATGTTATGTTCTGAGGGGAGTGAAAATTCCCCTAATTCGATGAAGATTCTTGCTCAATTGTTATCAGCTATGCGCCGACCAGAACACCTTGCCGATCAGCCAAACGTCTCTTCAGGCCACTGACTAGCGATAACTTTCCCCACAACGGAACAACTCTCATTGCATGGGATCATTGGGTACTGTGGGTTTAGTGGTTGTAAAAACACCTGACCGCTATCCCTGATCAGTTTCTTGAAGGTAAACTCATCACCCCCAAGTCTGGCTATGCAGAAATCACCTGGCTCAACAGCCTGCTCAGGGTCAACGAGAATTAACATTCCGTCAGGAAAGCTTGGCTTGGAGCCTGTTGGTGCGGTCATGGAATTACCTTCAACCTCAAGCCAGAATGCAGAATCACTGGCTTTTTTGGTTGTGCTTACCCATCTCTCCGCATCACCTTTGGTAAAGGTTCTAAGCTTAGGTGAGAACATCCCTGCCTGAACATGAGAAAAAACAGGGTACTCATACTCACTTCTAAGTGACGGCTGCATACTAACCGCTTCATACATCTCGTAGATTTCTCTGGCGATTGAAGGGCTAAATTCTTCAACGCTAACTTTGAGAATTTTTGTAAGCAATGCGGCGTTATAAGCATTTAATGCATTGATGCCATTAAATAAAGCACCAACGCCTGACTGCCCCATCCCCATCTTGTCTGCGACAGATTCCTGGGATAAGCCAAGTTCATTTTTCTTTTTTTCATAAATTGCTTTAAGGCGACGTGCGTCCTCAAGCTGCTCTTGTGTTAATGGTTTCTTTTTTGTGCTCATACGTTAAATCTATCACCGCAAGGGATAAATATCTAACACCGTGCGTGTTGACTATTTTACCTCTGGCGGTGATAATGGTTGCATGTACTAAGGAGGTTGTATGGAACAACGCATAACCCTGAAAGATTATGCAATGCGCTTTGGGCAAACCAAGACAGCTAAAGATCTCGGCGTATATCAAAGCGCGATCAACAAGGCCATTCATGCAGGCCGAAAGATTTTTTTAACTATAAACGCTGATGGAAGCGTTTATGCGGAAGAGGTAAAGCCCTTCCCGAGTAACAAAAAAACAACAGCATAAATAACCCCGCTCTTACACATTCCAGCCCTGAAAAAGGGCATCAAATTAAACCACACCTATGGTGTATGCATTTATTTGCATACATTCAATCAATTGTTATCTAAGGAAATACTTACATATGGTTCGTGCAAACAAACGCAACGAGGCTCTACGAATCGAGAGTGCGTTGCTTAACAAAATCGCAATGCTTGGAACTGAGAAGACAGCGGAAGCTGTGGGCGTTGATAAGTCGCGTCGACAAGCTTCCGGCCGCAGTCGAGCACCACCACCACCACCACTGAGATCCGGCTGCTAACAAAGCCCGAAAGGAAGCTGAGTTGGCTGCTGCCACCGCTGAGCAATAACTAGCATAACCCCTTGGGGCCTCTAAACGGGTCTTGAGGGGTTTTTTGCTGAAAGGAGGAACTATATCCGGATTGGCGAATGGGACGCGCCCTGTAGCGGCGCATTAAGCGCGGCGGGTGTGGTGGTTACGCGCAGCGTGACCGCTACACTTGCCAGCGCCCTAGCGCCCGCTCCTTTCGCTTTCTTCCCTTCCTTTCTCGCCACGTTCGCCGGCTTTCCCCGTCAAGCTCTAAATCGGGGGCTCCCTTTAGGGTTCCGATTTAGTGCTTTACGGCACCTCGACCCCAAAAAACTTGATTAGGGTGATGGTTCACGTAGTGGGCCATCGCCCTGATAGACGGTTTTTCGCCCTTTGACGTTGGAGTCCACGTTCTTTAATAGTGGACTCTTGTTCCAAACTGGAACAACACTCAACCCTATCTCGGTCTATTCTTTTGATTTATAAGGGATTTTGCCGATTTCGGCCTATTGGTTAAAAAATGAGCTGATTTAACAAAAATTTAACGCGAATTTTAACAAAATATTAACGTTTACAATTTCAGGTGGCACTTTTCGGGGAAATGTGCGCGGAACCCCTATTTGTTTATTTTTCTAAATACATTCAAATATGTATCCGCTCATGAATTAATTCTTAGAAAAACTCATCGAGCATCAAATGAAACTGCAATTTATTCATATCAGGATTATCAATACCATATTTTTGAAAAAGCCGTTTCTGTAATGAAGGAGAAAACTCACCGAGGCAGTTCCATAGGATGGCAAGATCCTGGTATCGGTCTGCGATTCCGACTCGTCCAACATCAATACAACCTATTAATTTCCCCTCGTCAAAAATAAGGTTATCAAGTGAGAAATCACCATGAGTGACGACTGAATCCGGTGAGAATGGCAAAAGTTTATGCATTTCTTTCCAGACTTGTTCAACAGGCCAGCCATTACGCTCGTCATCAAAATCACTCGCATCAACCAAACCGTTATTCATTCGTGATTGCGCCTGAGCGAGACGAAATACGCGATCGCTGTTAAAAGGACAATTACAAACAGGAATCGAATGCAACCGGCGCAGGAACACTGCCAGCGCATCAACAATATTTTCACCTGAATCAGGATATTCTTCTAATACCTGGAATGCTGTTTTCCCGGGGATCGCAGTGGTGAGTAACCATGCATCATCAGGAGTACGGATAAAATGCTTGATGGTCGGAAGAGGCATAAATTCCGTCAGCCAGTTTAGTCTGACCATCTCATCTGTAACATCATTGGCAACGCTACCTTTGCCATGTTTCAGAAACAACTCTGGCGCATCGGGCTTCCCATACAATCGATAGATTGTCGCACCTGATTGCCCGACATTATCGCGAGCCCATTTATACCCATATAAATCAGCATCCATGTTGGAATTTAATCGCGGCCTAGAGCAAGACGTTTCCCGTTGAATATGGCTCATAACACCCCTTGTATTACTGTTTATGTAAGCAGACAGTTTTATTGTTCATGACCAAAATCCCTTAACGTGAGTTTTCGTTCCACTGAGCGTCAGACCCCGTAGAAAAGATCAAAGGATCTTCTTGAGATCCTTTTTTTCTGCGCGTAATCTGCTGCTTGCAAACAAAAAAACCACCGCTACCAGCGGTGGTTTGTTTGCCGGATCAAGAGCTACCAACTCTTTTTCCGAAGGTAACTGGCTTCAGCAGAGCGCAGATACCAAATACTGTCCTTCTAGTGTAGCCGTAGTTAGGCCACCACTTCAAGAACTCTGTAGCACCGCCTACATACCTCGCTCTGCTAATCCTGTTACCAGTGGCTGCTGCCAGTGGCGATAAGTCGTGTCTTACCGGGTTGGACTCAAGACGATAGTTACCGGATAAGGCGCAGCGGTCGGGCTGAACGGGGGGTTCGTGCACACAGCCCAGCTTGGAGCGAACGACCTACACCGAACTGAGATACCTACAGCGTGAGCTATGAGAAAGCGCCACGCTTCCCGAAGGGAGAAAGGCGGACAGGTATCCGGTAAGCGGCAGGGTCGGAACAGGAGAGCGCACGAGGGAGCTTCCAGGGGGAAACGCCTGGTATCTTTATAGTCCTGTCGGGTTTCGCCACCTCTGACTTGAGCGTCGATTTTTGTGATGCTCGTCAGGGGGGCGGAGCCTATGGAAAAACGCCAGCAACGCGGCCTTTTTACGGTTCCTGGCCTTTTGCTGGCCTTTTGCTCACATGTTCTTTCCTGCGTTATCCCCTGATTCTGTGGATAACCGTATTACCGCCTTTGAGTGAGCTGATACCGCTCGCCGCAGCCGAACGACCGAGCGCAGCGAGTCAGTGAGCGAGGAAGCGGAAGAGCGCCTGATGCGGTATTTTCTCCTTACGCATCTGTGCGGTATTTCACACCGCATATATGGTGCACTCTCAGTACAATCTGCTCTGATGCCGCATAGTTAAGCCAGTATACACTCCGCTATCGCTACGTGACTGGGTCATGGCTGCGCCCCGACACCCGCCAACACCCGCTGACGCGCCCTGACGGGCTTGTCTGCTCCCGGCATCCGCTTACAGACAAGCTGTGACCGTCTCCGGGAGCTGCATGTGTCAGAGGTTTTCACCGTCATCACCGAAACGCGCGAGGCAGCTGCGGTAAAGCTCATCAGCGTGGTCGTGAAGCGATTCACAGATGTCTGCCTGTTCATCCGCGTCCAGCTCGTTGAGTTTCTCCAGAAGCGTTAATGTCTGGCTTCTGATAAAGCGGGCCATGTTAAGGGCGGTTTTTTCCTGTTTGGTCACTGATGCCTCCGTGTAAGGGGGATTTCTGTTCATGGGGGTAATGATACCGATGAAACGAGAGAGGATGCTCACGATACGGGTTACTGATGATGAACATGCCCGGTTACTGGAACGTTGTGAGGGTAAACAACTGGCGGTATGGATGCGGCGGGACCAGAGAAAAATCACTCAGGGTCAATGCCAGCGCTTCGTTAATACAGATGTAGGTGTTCCACAGGGTAGCCAGCAGCATCCTGCGATGCAGATCCGGAACATAATGGTGCAGGGCGCTGACTTCCGCGTTTCCAGACTTTACGAAACACGGAAACCGAAGACCATTCATGTTGTTGCTCAGGTCGCAGACGTTTTGCAGCAGCAGTCGCTTCACGTTCGCTCGCGTATCGGTGATTCATTCTGCTAACCAGTAAGGCAACCCCGCCAGCCTAGCCGGGTCCTCAACGACAGGAGCACGATCATGCGCACCCGTGGGGCCGCCATGCCGGCGCCTGCCACCATACCCACGCCGAAACAAGCGCTCATGAGCCCGAAGTGGCGAGCCCGATCTTCCCCATCGGTGATGTCGGCGATATAGGCGCCAGCAACCGCACCTGTGGCGCCGGTGATGCCGGCCACGATGCGTCCGGCGTAGAGGATCGAGATCTCGATCCCGCGAAATTAATACGACTCACTATAGGGGAATTGTGAGCGGATAACAATTCCCTTCTAGCCATATTGGACTCGGACCTGTTTCACGTGGAACACTGAGCCTGGACTAGGTCTAGCATATTGGACTCGACTTTCACGTGGAACACTGAGCCTGGACTAGGTCTAGAACAGGTTCTTTTTTCTTTGTTTCACGTGGAACATTCTGATTAATGTACAGCTAGCCATATTGGACTCGGACCTGTTTCACGTGGAACACTGAGCCTGGACTAGGTCTAGAACAGGTTCTTTTTTCTTTGTTTCACGTGGAACATTCTGATTAATGTACAGCTAGAACAGGTTCTTTTTTCTTTGTTTCACGTGGAACATTCTGATTAATGTACAGCTAGAAATAATTTTGTTTAACTTTAAGAAGGAGATATACCATGTGTTTCACGTGGAACACATGGCTAAAGGCCTTGGAAAAGGGATTAATGCGTTATTTAATCAGGTAGATTTGTCTGAAGAGACAGTTGAAGAAATTAAAATTGCCGATTTACGCCCTAATCCTTATCAGCCAAGAAAACACTTTGATGACGAGGCATTAGCTGAACTAAAAGAATCTGTGCTGCAGCATGGCATTCTTCAGCCGCTTATCGTCAGAAAATCTTTAAAAGGCTATGATATTGTTGCGGGTGAACGGCGTTTTCGAGCGGCAAAGCTGGCAGGTTTAGATACAGTTCCGGCCATTGTCCGTGAATTATCAGAGGCGTTAATGAGGGAAATTGCTTTATTAGAAAACCTTCAGCGTGAAGATTTATCTCCGCTTGAAGAGGCTCAGGCATATGCCGAAACAAGCGCTCATGAGCCCGAAGTGGCGAGCCCGATCTTCCCCATCGGTGATGTCGGCGATATAGGCGCCAGCAACCGCACCTGTGGCGCCGGTGATGCCGGCCACGATGCGTCCGGCGTAGAGGATCGAGATCTCGATCCCGCGAAATTAATACGACTCACTATAGGGGAATTGTGAGCGGATAACAATTCCCTTCTAGCCATATTGGACTCGGACCTGTTTCACGTGGAACACTGAGCCTGGACTAGGTCTAGCATATTGGACTCGACTTTCACGTGGAACACTGAGCCTGGACTAGGTCTAGAACAGGTTCTTTTTTCTTTGTTTCACGTGGAACATTCTGATTAATGTACAGCTAGCCATATTGGACTCGGACCTGTTTCACGTGGAACACTGAGCCTGGACTAGGTCTAGAACAGGTTCTTTTTTCTTTGTTTCACGTGGAACATTCTGATTAATGTACAGCTAGAACAGGTTCTTTTTTCTTTGTTTCACGTGGAACATTCTGATTAATGTACAGCTAGAAATAATTTTGTTTAACTTTAAGAAGGAGATATACCATGTGTTTCACGTGGAACACATGGCTAAAGGCCTTGGAAAAGGGATTAATGCGCATATGACTCCCTTTTGAAACACTTAGATCTCACACAAGAGCAGCTTGCCAAACGTCTTGGGAAAAGCAGACCGCATATTGCGAATCATTTAAGACTGCTGACACTGCCAGAAAATATTCAACAGCTTATTGCCGAAGGCACGCTTTCTATGGGACATGGACGCACGCTTCTTGGCTTAAAAAACAAAAATAAGCTTGAACCGCTGGTACAAAAAGTGATTGCGGAGCAGCTCAATGTTCGCCAACTTGAGCAGCTGATTCAGCAGTTGAATCAGAATGTTCCACGTGAAACAAAGAAAAAAGAACCTGTGAAAGATGCGGTTCGCCATGCCGGCGCCTGCCACCATACCCACGCCGAAACAAGCGCTCATGAGCCCGAAGTGGCGAGCCCGATCTTCCCCATCGGTGATGTCGGCGATATAGGCGCCAGCAACCGCACCTGTGGCGCCGGTGATGCCGGCCACGATGCGTCCGGCGTAGAGGATCGAGATCTCGATCCCGCGAAATTAATACGACTCACTATAGGGGAATTGTGAGCGGATAACAATTCCCTTCTAGCCATATTGGACTCGGACCTGTTTCACGTGGAACACTGAGCCTGGACTAGGTCTAGCATATTGGACTCGACTTTCACGTGGAACACTGAGCCTGGACTAGGTCTAGAACAGGTTCTTTTTTCTTTGTTTCACGTGGAACATTCTGATTAATGTACAGCTAGCCATATTGGACTCGGACCTGTTTCACGTGGAACACTGAGCCTGGACTAGGTCTAGAACAGGTTCTTTTTTCTTTGTTTCACGTGGAACATTCTGATTAATGTACAGCTAGAACAGGTTCTTTTTTCTTTGTTTCACGTGGAACATTCTGATTAATGTACAGCTAGAAATAATTTTGTTTAACTTTAAGAAGGAGATATACCATGTGTTTCACGTGGAACACATGGCTAAAGGCCTTGGAAAAGGGATTAATGCGTTATTTAATCAGGTAGATTTGTCTGAAGAGACAGTTGAAGAAATTAAAATTGCCGATTTACGCCCTAATCCTTATCAGCCAAGAAAACACTTTGATGACGAGGCATTAGCTGAACTAAAAGAATCTGTGCTGCAGCATGGCATTCTTCAGCCGCTTATCGTCAGAAAATCTTTAAAAGGCTATGATATTGTTGCGGGTGAACGGCGTTTTCGAGCGGCAAAGCTGGCAGGTTTAGATACAGTTCCGGCCATTGTCCGTGAATTATCAGAGGCGTTAATGAGGGAAATTGCTTTATTAGAAAACCTTCAGCGTGAAGATTTATCTCCGCTTGAAGAGGCTCAGGCATATGCCGAAACAAGCGCTCATGAGCCCGAAGTGGCGAGCCCGATCTTCCCCATCGGTGATGTCGGCGATATAGGCGCCAGCAACCGCACCTGTGGCGCCGGTGATGCCGGCCACGATGCGTCCGGCGTAGAGGATCGAGATCTCGATCCCGCGAAATTAATACGACTCACTATAGGGGAATTGTGAGCGGATAACAATTCCCTTCTAGCCATATTGGACTCGGACCTGTTTCACGTGGAACACTGAGCCTGGACTAGGTCTAGCATATTGGACTCGACTTTCACGTGGAACACTGAGCCTGGACTAGGTCTAGAACAGGTTCTTTTTTCTTTGTTTCACGTGGAACATTCTGATTAATGTACAGCTAGCCATATTGGACTCGGACCTGTTTCACGTGGAACACTGAGCCTGGACTAGGTCTAGAACAGGTTCTTTTTTCTTTGTTTCACGTGGAACATTCTGATTAATGTACAGCTAGAACAGGTTCTTTTTTCTTTGTTTCACGTGGAACATTCTGATTAATGTACAGCTAGAAATAATTTTGTTTAACTTTAAGAAGGAGATATACCATGTGTTTCACGTGGAACACATGGCTAAAGGCCTTGGAAAAGGGATTAATGCGCATATGACTCCCTTTTGAAACACTTAGATCTCACACAAGAGCAGCTTGCCAAACGTCTTGGGAAAAGCAGACCGCATATTGCGAATCATTTAAGACTGCTGACACTGCCAGAAAATATTCAACAGCTTATTGCCGAAGGCACGCTTTCTATGGGACATGGACGCACGCTTCTTGGCTTAAAAAACAAAAATAAGCTTGAACCGCTGGTACAAAAAGTGATTGCGGAGCAGCTCAATGTTCGCCAACTTGAGCAGCTGATTCAGCAGTTGAATCAGAATGTTCCACGTGAAACAAAGAAAAAAGAACCTGTGAAAGATGCGGTTCGCCATGCCGGCGATAATGGCCTGCTTCTCGCCGAAACGTTTGGTGGCGGGACCAGTGACGAAGGCTTGAGCGAGGGCGTGCAAGATTCCGAATACCGCAAGCGACAGGCCGATCATCGTCGCGCTCCAGCGAAAGCGGTCCTCGCCGAAAATGACCCAGAGCGCTGCCGGCACCTGTCCTACGAGTTGCATGATAAAGAAGACAGTCATAAGTGCGGCGACGATAGTCATGCCCCGCGCCCACCGGAAGGAGCTGACTGGGTTGAAGGCTCTCAAGGGCATCGGTCGAGATCCCGGTGCCTAATGAGTGAGCTAACTTACATTAATTGCGTTGCGCTCACTGCCCGCTTTCCAGTCGGGAAACCTGTCGTGCCAGCTGCATTAATGAATCGGCCAACGCGCGGGGAGAGGCGGTTTGCGTATTGGGCGCCAGGGTGGTTTTTCTTTTCACCAGTGAGACGGGCAACAGCTGATTGCCCTTCACCGCCTGGCCCTGAGAGAGTTGCAGCAAGCGGTCCACGCTGGTTTGCCCCAGCAGGCGAAAATCCTGTTTGATGGTGGTTAACGGCGGGATATAACATGAGCTGTCTTCGGTATCGTCGTATCCCACTACCGAGATATCCGCACCAACGCGCAGCCCGGACTCGGTAATGGCGCGCATTGCGCCCAGCGCCATCTGATCGTTGGCAACCAGCATCGCAGTGGGAACGATGCCCTCATTCAGCATTTGCATGGTTTGTTGAAAACCGGACATGGCACTCCAGTCGCCTTCCCGTTCCGCTATCGGCTGAATTTGATTGCGAGTGAGATATTTATGCCAGCCAGCCAGACGCAGACGCGCCGAGACAGAACTTAATGGGCCCGCTAACAGCGCGATTTGCTGGTGACCCAATGCGACCAGATGCTCCACGCCCAGTCGCGTACCGTCTTCATGGGAGAAAATAATACTGTTGATGGGTGTCTGGTCAGAGACATCAAGAAATAACGCCGGAACATTAGTGCAGGCAGCTTCCACAGCAATGGCATCCTGGTCATCCAGCGGATAGTTAATGATCAGCCCACTGACGCGTTGCGCGAGAAGATTGTGCACCGCCGCTTTACAGGCTTCGACGCCGCTTCGTTCTACCATCGACACCACCACGCTGGCACCCAGTTGATCGGCGCGAGATTTAATCGCCGCGACAATTTGCGACGGCGCGTGCAGGGCCAGACTGGAGGTGGCAACGCCAATCAGCAACGACTGTTTGCCCGCCAGTTGTTGTGCCACGCGGTTGGGAATGTAATTCAGCTCCGCCATCGCCGCTTCCACTTTTTCCCGCGTTTTCGCAGAAACGTGGCTGGCCTGGTTCACCACGCGGGAAACGGTCTGATAAGAGACACCGGCATACTCTGCGACATCGTATAACGTTACTGGTTTCACATTCACCACCCTGAATTGACTCTCTTCCGGGCGCTATCATGCCATACCGCGAAAGGTTTTGCGCCATTCGATGGTGTCCGGGATCTCGACGCTCTCCCTTATGCGACTCCTGCATTAGGAAGCAGCCCAGTAGTAGGTTGAGGCCGTTGAGCACCGCCGCCGCAAGGAATGGTGCATGCAAGGAGATGGCGCCCAACAGTCCCCCGGCCACGGGGCCTGCCACCATACCCACGCCGAAACAAGCGCTCATGAGCCCGAAGTGGCGAGCCCGATCTTCCCCATCGGTGATGTCGGCGATATAGGCGCCAGCAACCGCACCTGTGGCGCCGGTGATGCCGGCCACGATGCGTCCGGCGTAGAGGATCGAGATCTCGATCCCGCGAAATTAATACGACTCACTATAGGGGAATTGTGAGCGGATAACAATTCCCTTCTAGCCATATTGGACTCGGACCTGTTTCACGTGGAACACTGAGCCTGGACTAGGTCTAGCATATTGGACTCGACTTTCACGTGGAACACTGAGCCTGGACTAGGTCTAGAACAGGTTCTTTTTTCTTTGTTTCACGTGGAACATTCTGATTAATGTACAGCTAGCCATATTGGACTCGGACCTGTTTCACGTGGAACACTGAGCCTGGACTAGGTCTAGAACAGGTTCTTTTTTCTTTGTTTCACGTGGAACATTCTGATTAATGTACAGCTAGAACAGGTTCTTTTTTCTTTGTTTCACGTGGAACATTCTGATTAATGTACAGCTAGAAATAATTTTGTTTAACTTTAAGAAGGAGATATACCATGTGTTTCACGTGGAACACATGGCTAAAGGCCTTGGAAAAGGGATTAATGCGTTATTTAATCAGGTAGATTTGTCTGAAGAGACAGTTGAAGAAATTAAAATTGCCGATTTACGCCCTAATCCTTATCAGCCAAGAAAACACTTTGATGACGAGGCATTAGCTGAACTAAAAGAATCTGTGCTGCAGCATGGCATTCTTCAGCCGCTTATCGTCAGAAAATCTTTAAAAGGCTATGATATTGTTGCGGGTGAACGGCGTTTTCGAGCGGCAAAGCTGGCAGGTTTAGATACAGTTCCGGCCATTGTCCGTGAATTATCAGAGGCGTTAATGAGGGAAATTGCTTTATTAGAAAACCTTCAGCGTGAAGATTTATCTCCGCTTGAAGAGGCTCAGGCATATGCCGAAACAAGCGCTCATGAGCCCGAAGTGGCGAGCCCGATCTTCCCCATCGGTGATGTCGGCGATATAGGCGCCAGCAACCGCACCTGTGGCGCCGGTGATGCCGGCCACGATGCGTCCGGCGTAGAGGATCGAGATCTCGATCCCGCGAAATTAATACGACTCACTATAGGGGAATTGTGAGCGGATAACAATTCCCTTCTAGCCATATTGGACTCGGACCTGTTTCACGTGGAACACTGAGCCTGGACTAGGTCTAGCATATTGGACTCGACTTTCACGTGGAACACTGAGCCTGGACTAGGTCTAGAACAGGTTCTTTTTTCTTTGTTTCACGTGGAACATTCTGATTAATGTACAGCTAGCCATATTGGACTCGGACCTGTTTCACGTGGAACACTGAGCCTGGACTAGGTCTAGAACAGGTTCTTTTTTCTTTGTTTCACGTGGAACATTCTGATTAATGTACAGCTAGAACAGGTTCTTTTTTCTTTGTTTCACGTGGAACATTCTGATTAATGTACAGCTAGAAATAATTTTGTTTAACTTTAAGAAGGAGATATACCATGTGTTTCACGTGGAACACATGGCTAAAGGCCTTGGAAAAGGGATTAATGCGCATATGACTCCCTTTTGAAACACTTAGATCTCACACAAGAGCAGCTTGCCAAACGTCTTGGGAAAAGCAGACCGCATATTGCGAATCATTTAAGACTGCTGACACTGCCAGAAAATATTCAACAGCTTATTGCCGAAGGCACGCTTTCTATGGGACATGGACGCACGCTTCTTGGCTTAAAAAACAAAAATAAGCTTGAACCGCTGGTACAAAAAGTGATTGCGGAGCAGCTCAATGTTCGCCAACTTGAGCAGCTGATTCAGCAGTTGAATCAGAATGTTCCACGTGAAACAAAGAAAAAAGAACCTGTGAAAGATGCGGTTCTAAAAGAACGGGAATCCTATCTCCAAAATTATTTTGGAACAACAGTTAATATTAAAAGACAGAAGAAAAAAGGCAAAATCGAAATTGAATTTTTCTCTAATGAAGACCTTGACCGGATTTTAGAGCTTTTGTCTGAACGAGAATCATAAGGATCCTCAACTGTGAGGAGGCTCACGGACGCGAAGAACAGGCACGCGTGCTGGCAGAAACCCCCGGTATGACCGTGAAAACGGCCCGCCGCATTCTGGCCGCAGCACCACAGAGTGCACAGGCGCGCAGTGACACTGCGCTGGATCGTCTGATGCAGGGGGCACCGGCACCGCTGGCTGCAGGTAACCCGGCATCTGATGCCGTTAACGATTTGCTGAACACACCAGTGTAAGGGATGTTTATGACGAGCAAAGAAACCTTTACCCATTACCAGCCGCAGGGCAACAGTGACCCGGCTCATACCGCAACCGCGCCCGGCGGATTGAGTGCGAAAGCGCCTGCAATGACCCCGCTGATGCTGGACACCTCCAGCCGTAAGCTGGTTGCGTGGGATGGCACCACCGACGGTGCTGCCGTTGGCATTCTTGCGGTTGCTGCTGACCAGACCAGCACCACGCTGACGTTCTACAAGTCCGGCACGTTCCGTTATGAGGATGTGCTCTGGCCGGAGGCTGCCAGCGACGAGACGAAAAAACGGACCGCGTTTGCCGGAACGGCAATCAGCATCGTTTAACTTTACCCTTCATCACTAAAGGCCGCCTGTGCGGCTTTTTTTACGGGATTTTTTTATGTCGATGTACACAACCGCCCAACTGCTGGCGGCAAATGAGCAGAAATTTAAGTTTGATCCGCTGTTTCTGCGTCTCTTTTTCCGTGAGAGCTATCCCTTCACCACGGAGAAAGTCTATCTCTCACAAATTCCGGGACTGGTAAACATGGCGCTGTACGTTTCGCCGATTGTTTCCGGTGAGGTTATCCGTTCCCGTGGCGGCTCCACCTCTGAATTTACGCCGGGATATGTCAAGCCGAAGCATGAAGTGAATCCGCAGATGACCCTGCGTCGCCTGCCGGATGAAGATCCGCAGAATCTGGCGGACCCGGCTTACCGCCGCCGTCGCATCATCATGCAGAACATGCGTGACGAAGAGCTGGCCATTGCTCAGGTCGAAGAGATGCAGGCAGTTTCTGCCGTGCTTAAGGGCAAATACACCATGACCGGTGAAGCCTTCGATCCGGTTGAGGTGGATATGGGCCGCAGTGAGGAGAATAACATCACGCAGTCCGGCGGCACGGAGTGGAGCAAGCGTGACAAGTCCACGTATGACCCGACCGACGATATCGAAGCCTACGCGCTGAACGCCAGCGGTGTGGTGAATATCATCGTGTTCGATCCGAAAGGCTGGGCGCTGTTCCGTTCCTTCAAAGCCGTCAAGGAGAAGCTGGATACCCGTCGTGGCTCTAATTCCGAGCTGGAGACAGCGGTGAAAGACCTGGGCAAAGCGGTGTCCTATAAGGGGATGTATGGCGATGTGGCCATCGTCGTGTATTCCGGACAGTACGTGGAAAACGGCGTCAAAAAGAACTTCCTGCCGGACAACACGATGGTGCTGGGGAACACTCAGGCACGCGGTCTGCGCACCTATGGCTGCATTCAGGATGCGGACGCACAGCGCGAAGGCATTAACGCCTCTGCCCGTTACCCGAAAAACTGGGTGACCACCGGCGATCCGGCGCGTGAGTTCACCATGATTCAGTCAGCACCGCTGATGCTGCTGGCTGACCCTGATGAGTTCGTGTCCGTACAACTGGCGTAATCATGGCCCTTCGGGGCCATTGTTTCTCTGTGGAGGAGTCCATGACGAAAGATGAACTGATTGCCCGTCTCCGCTCGCTGGGTGAACAACTGAACCGTGATGTCAGCCTGACGGGGACGAAAGAAGAACTGGCGCTCCGTGTGGCAGAGCTGAAAGAGGAGCTTGATGACACGGATGAAACTGCCGGTCAGGACACCCCTCTCAGCCGGGAAAATGTGCTGACCGGACATGAAAATGAGGTGGGATCAGCGCAGCCGGATACCGTGATTCTGGATACGTCTGAACTGGTCACGGTCGTGGCACTGGTGAAGCTGCATACTGATGCACTTCACGCCACGCGGGATGAACCTGTGGCATTTGTGCTGCCGGGAACGGCGTTTCGTGTCTCTGCCGGTGTGGCAGCCGAAATGACAGAGCGCGGCCTGGCCAGAATGCAATAACGGGAGGCGCTGTGGCTGATTTCGATAACCTGTTCGATGCTGCCATTGCCCGCGCCGATGAAACGATACGCGGGTACATGGGAACGTCAGCCACCATTACATCCGGTGAGCAGTCAGGTGCGGTGATACGTGGTGTTTTTGATGACCCTGAAAATATCAGCTATGCCGGACAGGGCGTGCGCGTTGAAGGCTCCAGCCCGTCCCTGTTTGTCCGGACTGATGAGGTGCGGCAGCTGCGGCGTGGAGACACGCTGACCATCGGTGAGGAAAATTTCTGGGTAGATCGGGTTTCGCCGGATGATGGCGGAAGTTGTCATCTCTGGCTTGGACGGGGCGTACCGCCTGCCGTTAACCGTCGCCGCTGAAAGGGGGATGTATGGCCATAAAAGGTCTTGAGCAGGCCGTTGAAAACCTCAGCCGTATCAGCAAAACGGCGGTGCCTGGTGCCGCCGCAATGGCCATTAACCGCGTTGCTTCATCCGCGATATCGCAGTCGGCGTCACAGGTTGCCCGTGAGACAAAGGTACGCCGGAAACTGGTAAAGGAAAGGGCCAGGCTGAAAAGGGCCACGGTCAAAAATCCGCAGGCCAGAATCAAAGTTAACCGGGGGGATTTGCCCGTAATCAAGCTGGGTAATGCGCGGGTTGTCCTTTCGCGCCGCAGGCGTCGTAAAAAGGGGCAGCGTTCATCCCTGAAAGGTGGCGGCAGCGTGCTTGTGGTGGGTAACCGTCGTATTCCCGGCGCGTTTATTCAGCAACTGAAAAATGGCCGGTGGCATGTCATGCAGCGTGTGGCTGGGAAAAACCGTTACCCCATTGATGTGGTGAAAATCCCGATGGCGGTGCCGCTGACCACGGCGTTTAAACAAAATATTGAGCGGATACGGCGTGAACGTCTTCCGAAAGAGCTGGGCTATGCGCTGCAGCATCAACTGAGGATGGTAATAAAGCGATGAAACATACTGAACTCCGTGCAGCCGTACTGGATGCACTGGAGAAGCATGACACCGGGGCGACGTTTTTTGATGGTCGCCCCGCTGTTTTTGATGAGGCGGATTTTCCGGCAGTTGCCGTTTATCTCACCGGCGCTGAATACACGGGCGAAGAGCTGGACAGCGATACCTGGCAGGCGGAGCTGCATATCGAAGTTTTCCTGCCTGCTCAGGTGCCGGATTCAGAGCTGGATGCGTGGATGGAGTCCCGGATTTATCCGGTGATGAGCGATATCCCGGCACTGTCAGATTTGATCACCAGTATGGTGGCCAGCGGCTATGACTACCGGCGCGACGATGATGCGGGCTTGTGGAGTTCAGCCGATCTGACTTATGTCATTACCTATGAAATGTGAGGACGCTATGCCTGTACCAAATCCTACAATGCCGGTGAAAGGTGCCGGGACCACCCTGTGGGTTTATAAGGGGAGCGGTGACCCTTACGCGAATCCGCTTTCAGACGTTGACTGGTCGCGTCTGGCAAAAGTTAAAGACCTGACGCCCGGCGAACTGACCGCTGAGTCCTATGACGACAGCTATCTCGATGATGAAGATGCAGACTGGACTGCGACCGGGCAGGGGCAGAAATCTGCCGGAGATACCAGCTTCACGCTGGCGTGGATGCCCGGAGAGCAGGGGCAGCAGGCGCTGCTGGCGTGGTTTAATGAAGGCGATACCCGTGCCTATAAAATCCGCTTCCCGAACGGCACGGTCGATGTGTTCCGTGGCTGGGTCAGCAGTATCGGTAAGGCGGTGACGGCGAAGGAAGTGATCACCCGCACGGTGAAAGTCACCAATGTGGGACGTCCGTCGATGGCAGAAGATCGCAGCACGGTAACAGCGGCAACCGGCATGACCGTGACGCCTGCCAGCACCTCGGTGGTGAAAGGGCAGAGCACCACGCTGACCGTGGCCTTCCAGCCGGAGGGCGTAACCGACAAGAGCTTTCGTGCGGTGTCTGCGGATAAAACAAAAGCCACCGTGTCGGTCAGTGGTATGACCATCACCGTGAACGGCGTTGCTGCAGGCAAGGTCAACATTCCGGTTGTATCCGGTAATGGTGAGTTTGCTGCGGTTGCAGAAATTACCGTCACCGCCAGTTAATCCGGAGAGTCAGCGATGTTCCTGAAAACCGAATCATTTGAACATAACGGTGTGACCGTCACGCTTTCTGAACTGTCAGCCCTGCAGCGCATTGAGCATCTCGCCCTGATGAAACGGCAGGCAGAACAGGCGGAGTCAGACAGCAACCGGAAGTTTACTGTGGAAGACGCCATCAGAACCGGCGCGTTTCTGGTGGCGATGTCCCTGTGGCATAACCATCCGCAGAAGACGCAGATGCCGTCCATGAATGAAGCCGTTAAACAGATTGAGCAGGAAGTGCTTACCACCTGGCCCACGGAGGCAATTTCTCATGCTGAAAACGTGGTGTACCGGCTGTCTGGTATGTATGAGTTTGTGGTGAATAATGCCCCTGAACAGACAGAGGACGCCGGGCCCGCAGAGCCTGTTTCTGCGGGAAAGTGTTCGACGGTGAGCTGAGTTTTGCCCTGAAACTGGCGCGTGAGATGGGGCGACCCGACTGGCGTGCCATGCTTGCCGGGATGTCATCCACGGAGTATGCCGACTGGCACCGCTTTTACAGTACCCATTATTTTCATGATGTTCTGCTGGATATGCACTTTTCCGGGCTGACGTACACCGTGCTCAGCCTGTTTTTCAGCGATCCGGATATGCATCCGCTGGATTTCAGTCTGCTGAACCGGCGCGAGGCTGACGAAGAGCCTGAAGATGATGTGCTGATGCAGAAAGCGGCAGGGCTTGCCGGAGGTGTCCGCTTTGGCCCGGACGGGAATGAAGTTATCCCCGCTTCCCCGGATGTGGCGGACATGACGGAGGATGACGTAATGCTGATGACAGTATCAGAAGGGATCGCAGGAGGAGTCCGGTATGGCTGAACCGGTAGGCGATCTGGTCGTTGATTTGAGTCTGGATGCGGCCAGATTTGACGAGCAGATGGCCAGAGTCAGGCGTCATTTTTCTGGTACGGAAAGTGATGCGAAAAAAACAGCGGCAGTCGTTGAACAGTCGCTGAGCCGACAGGCGCTGGCTGCACAGAAAGCGGGGATTTCCGTCGGGCAGTATAAAGCCGCCATGCGTATGCTGCCTGCACAGTTCACCGACGTGGCCACGCAGCTTGCAGGCGGGCAAAGTCCGTGGCTGATCCTGCTGCAACAGGGGGGGCAGGTGAAGGACTCCTTCGGCGGGATGATCCCCATGTTCAGGGGGCTTGCCGGTGCGATCACCCTGCCGATGGTGGGGGCCACCTCGCTGGCGGTGGCGACCGGTGCGCTGGCGTATGCCTGGTATCAGGGCAACTCAACCCTGTCCGATTTCAACAAAACGCTGGTCCTTTCCGGCAATCAGGCGGGACTGACGGCAGATCGTATGCTGGTCCTGTCCAGAGCCGGGCAGGCGGCAGGGCTGACGTTTAACCAGACCAGCGAGTCACTCAGCGCACTGGTTAAGGCGGGGGTAAGCGGTGAGGCTCAGATTGCGTCCATCAGCCAGAGTGTGGCGCGTTTCTCCTCTGCATCCGGCGTGGAGGTGGACAAGGTCGCTGAAGCCTTCGGGAAGCTGACCACAGACCCGACGTCGGGGCTGACGGCGATGGCTCGCCAGTTCCATAACGTGTCGGCGGAGCAGATTGCGTATGTTGCTCAGTTGCAGCGTTCCGGCGATGAAGCCGGGGCATTGCAGGCGGCGAACGAGGCCGCAACGAAAGGGTTTGATGACCAGACCCGCCGCCTGAAAGAGAACATGGGCACGCTGGAGACCTGGGCAGACAGGACTGCGCGGGCATTCAAATCCATGTGGGATGCGGTGCTGGATATTGGTCGTCCTGATACCGCGCAGGAGATGCTGATTAAGGCAGAGGCTGCGTATAAGAAAGCAGACGACATCTGGAATCTGCGCAAGGATGATTATTTTGTTAACGATGAAGCGCGGGCGCGTTACTGGGATGATCGTGAAAAGGCCCGTCTTGCGCTTGAAGCCGCCCGAAAGAAGGCTGAGCAGCAGACTCAACAGGACAAAAATGCGCAGCAGCAGAGCGATACCGAAGCGTCACGGCTGAAATATACCGAAGAGGCGCAGAAGGCTTACGAACGGCTGCAGACGCCGCTGGAGAAATATACCGCCCGTCAGGAAGAACTGAACAAGGCACTGAAAGACGGGAAAATCCTGCAGGCGGATTACAACACGCTGATGGCGGCGGCGAAAAAGGATTATGAAGCGACGCTGAAAAAGCCGAAACAGTCCAGCGTGAAGGTGTCTGCGGGCGATCGTCAGGAAGACAGTGCTCATGCTGCCCTGCTGACGCTTCAGGCAGAACTCCGGACGCTGGAGAAGCATGCCGGAGCAAATGAGAAAATCAGCCAGCAGCGCCGGGATTTGTGGAAGGCGGAGAGTCAGTTCGCGGTACTGGAGGAGGCGGCGCAACGTCGCCAGCTGTCTGCACAGGAGAAATCCCTGCTGGCGCATAAAGATGAGACGCTGGAGTACAAACGCCAGCTGGCTGCACTTGGCGACAAGGTTACGTATCAGGAGCGCCTGAACGCGCTGGCGCAGCAGGCGGATAAATTCGCACAGCAGCAACGGGCAAAACGGGCCGCCATTGATGCGAAAAGCCGGGGGCTGACTGACCGGCAGGCAGAACGGGAAGCCACGGAACAGCGCCTGAAGGAACAGTATGGCGATAATCCGCTGGCGCTGAATAACGTCATGTCAGAGCAGAAAAAGACCTGGGCGGCTGAAGACCAGCTTCGCGGGAACTGGATGGCAGGCCTGAAGTCCGGCTGGAGTGAGTGGGAAGAGAGCGCCACGGACAGTATGTCGCAGGTAAAAAGTGCAGCCACGCAGACCTTTGATGGTATTGCACAGAATATGGCGGCGATGCTGACCGGCAGTGAGCAGAACTGGCGCAGCTTCACCCGTTCCGTGCTGTCCATGATGACAGAAATTCTGCTTAAGCAGGCAATGGTGGGGATTGTCGGGAGTATCGGCAGCGCCATTGGCGGGGCTGTTGGTGGCGGCGCATCCGCGTCAGGCGGTACAGCCATTCAGGCCGCTGCGGCGAAATTCCATTTTGCAACCGGAGGATTTACGGGAACCGGCGGCAAATATGAGCCAGCGGGGATTGTTCACCGTGGTGAGTTTGTCTTCACGAAGGAGGCAACCAGCCGGATTGGCGTGGGGAATCTTTACCGGCTGATGCGCGGCTATGCCACCGGCGGTTATGTCGGTACACCGGGCAGCATGGCAGACAGCCGGTCGCAGGCGTCCGGGACGTTTGAGCAGAATAACCATGTGGTGATTAACAACGACGGCACGAACGGGCAGATAGGTCCGGCTGCTCTGAAGGCGGTGTATGACATGGCCCGCAAGGGTGCCCGTGATGAAATTCAGACACAGATGCGTGATGGTGGCCTGTTCTCCGGAGGTGGACGATGAAGACCTTCCGCTGGAAAGTGAAACCCGGTATGGATGTGGCTTCGGTCCCTTCTGTAAGAAAGGTGCGCTTTGGTGATGGCTATTCTCAGCGAGCGCCTGCCGGGCTGAATGCCAACCTGAAAACGTACAGCGTGACGCTTTCTGTCCCCCGTGAGGAGGCCACGGTACTGGAGTCGTTTCTGGAAGAGCACGGGGGCTGGAAATCCTTTCTGTGGACGCCGCCTTATGAGTGGCGGCAGATAAAGGTGACCTGCGCAAAATGGTCGTCGCGGGTCAGTATGCTGCGTGTTGAGTTCAGCGCAGAGTTTGAACAGGTGGTGAACTGATGCAGGATATCCGGCAGGAAACACTGAATGAATGCACCCGTGCGGAGCAGTCGGCCAGCGTGGTGCTCTGGGAAATCGACCTGACAGAGGTCGGTGGAGAACGTTATTTTTTCTGTAATGAGCAGAACGAAAAAGGTGAGCCGGTCACCTGGCAGGGGCGACAGTATCAGCCGTATCCCATTCAGGGGAGCGGTTTTGAACTGAATGGCAAAGGCACCAGTACGCGCCCCACGCTGACGGTTTCTAACCTGTACGGTATGGTCACCGGGATGGCGGAAGATATGCAGAGTCTGGTCGGCGGAACGGTGGTCCGGCGTAAGGTTTACGCCCGTTTTCTGGATGCGGTGAACTTCGTCAACGGAAACAGTTACGCCGATCCGGAGCAGGAGGTGATCAGCCGCTGGCGCATTGAGCAGTGCAGCGAACTGAGCGCGGTGAGTGCCTCCTTTGTACTGTCCACGCCGACGGAAACGGATGGCGCTGTTTTTCCGGGACGTATCATGCTGGCCAACACCTGCACCTGGACCTATCGCGGTGACGAGTGCGGTTATAGCGGTCCGGCTGTCGCGGATGAATATGACCAGCCAACGTCCGATATCACGAAGGATAAATGCAGCAAATGCCTGAGCGGTTGTAAGTTCCGCAATAACGTCGGCAACTTTGGCGGCTTCCTTTCCATTAACAAACTTTCGCAGTAAATCCCATGACACAGACAGAATCAGCGATTCTGGCGCACGCCCGGCGATGTGCGCCAGCGGAGTCGTGCGGCTTCGTGGTAAGCACGCCGGAGGGGGAAAGATATTTCCCCTGCGTGAATATCTCCGGTGAGCCGGAGGCTATTTCCGTATGTCGCCGGAAGACTGGCTGCAGGCAGAAATGCAGGGTGAGATTGTGGCGCTGGTCCACAGCCACCCCGGTGGTCTGCCCTGGCTGAGTGAGGCCGACCGGCGGCTGCAGGTGCAGAGTGATTTGCCGTGGTGGCTGGTCTGCCGGGGGACGATTCATAAGTTCCGCTGTGTGCCGCATCTCACCGGGCGGCGCTTTGAGCACGGTGTGACGGACTGTTACACACTGTTCCGGGC

*C-trap EcoRI 39x parS DNA (17344 bp)*

GGCCGCGGTGTGCTCCTTATTTATACATAACGAAAAACGCCTCGAGTGAAGCGTTATTGGTATGCGGTAAAACCGCACTCAGGCGGCCTTGATAGTCATATCATCTGAATCAAATATTCCTGATGTATCGATATCGGTAATTCTTATTCCTTCGCTACCATCCATTGGAGGCCATCCTTCCTGACCATTTCCATCATTCCAGTCGAACTCACACACAACACCATATGCATTTAAGTCGCTTGAAATTGCTATAAGCAGAGCATGTTGCGCCAGCATGATTAATACAGCATTTAATACAGAGCCGTGTTTATTGAGTCGGTATTCAGAGTCTGACCAGAAATTATTAATCTGGTGAAGTTTTTCCTCTGTCATTACGTCATGGTCGATTTCAATTTCTATTGATGCTTTCCAGTCGTAATCAATGATGTATTTTTTGATGTTTGACATCTGTTCATATCCTCACAGATAAAAAATCGCCCTCACACTGGAGGGCAAAGAAGATTTCCAATAATCAGAACAAGTCGGCTCCTGTTTAGTTACGAGCGACATTGCTCCGTGTATTCACTCGTTGGAATGAATACACAGTGCAGTGTTTATTCTGTTATTTATGCCAAAAATAAAGGCCACTATCAGGCAGCTTTGTTGTTCTGTTTACCAAGTTCTCTGGCAATCATTGCCGTCGTTCGTATTGCCCATTTATCGACATATTTCCCATCTTCCATTACAGGAAACATTTCTTCAGGCTTAACCATGCATTCCGATTGCAGCTTGCATCCATTGCATCGCTTGAATTGTCCACACCATTGATTTTTATCAATAGTCGTAGTCATACGGATAGTCCTGGTATTGTTCCATCACATCCTGAGGATGCTCTTCGAACTCTTCAAATTCTTCTTCCATATATCACCTTAAATAGTGGATTGCGGTAGTAAAGATTGTGCCTGTCTTTTAACCACATCAGGCTCGGTGGTTCTCGTGTACCCCTACAGCGAGAAATCGGATAAACTATTACAACCCCTACAGTTTGATGAGTATAGAAATGGATCCACTCGTTATTCTCGGACGAGTGTTCAGTAATGAACCTCTGGAGAGAACCATGTATATGATCGTTATCTGGGTTGGACTTCTGCTTTTAAGCCCAGATAACTGGCCTGAATATGTTAATGAGAGAATCGGTATTCCTCATGTGTGGCATGTTTTCGTCTTTGCTCTTGCATTTTCGCTAGCAATTAATGTGCATCGATTATCAGCTATTGCCAGCGCCAGATATAAGCGATTTAAGCTAAGAAAACGCATTAAGATGCAAAACGATAAAGTGCGATCAGTAATTCAAAACCTTACAGAAGAGCAATCTATGGTTTTGTGCGCAGCCCTTAATGAAGGCAGGAAGTATGTGGTTACATCAAAACAATTCCCATACATTAGTGAGTTGATTGAGCTTGGTGTGTTGAACAAAACTTTTTCCCGATGGAATGGAAAGCATATATTATTCCCTATTGAGGATATTTACTGGACTGAATTAGTTGCCAGCTATGATCCATATAATATTGAGATAAAGCCAAGGCCAATATCTAAGTAACTAGATAAGAGGAATCGATTTTCCCTTAATTTTCTGGCGTCCACTGCATGTTATGCCGCGTTCGCCAGGCTTGCTGTACCATGTGCGCTGATTCTTGCGCTCAATACGTTGCAGGTTGCTTTCAATCTGTTTGTGGTATTCAGCCAGCACTGTAAGGTCTATCGGATTTAGTGCGCTTTCTACTCGTGATTTCGGTTTGCGATTCAGCGAGAGAATAGGGCGGTTAACTGGTTTTGCGCTTACCCCAACCAACAGGGGATTTGCTGCTTTCCATTGAGCCTGTTTCTCTGCGCGACGTTCGCGGCGGCGTGTTTGTGCATCCATCTGGATTCTCCTGTCAGTTAGCTTTGGTGGTGTGTGGCAGTTGTAGTCCTGAACGAAAACCCCCCGCGATTGGCACATTGGCAGCTAATCCGGAATCGCACTTACGGCCAATGCTTCGTTTCGTATCACACACCCCAAAGCCTTCTGCTTTGAATGCTGCCCTTCTTCAGGGCTTAATTTTTAAGAGCGTCACCTTCATGGTGGTCAGTGCGTCCTGCTGATGTGCTCAGTATCACCGCCAGTGGTATTTATGTCAACACCGCCAGAGATAATTTATCACCGCAGATGGTTATCTGTATGTTTTTTATATGAATTTATTTTTTGCAGGGGGGCATTGTTTGGTAGGTGAGAGATCTGAATTGCTATGTTTAGTGAGTTGTATCTATTTATTTTTCAATAAATACAATTGGTTATGTGTTTTGGGGGCGATCGTGAGGCAAAGAAAACCCGGCGCTGAGGCCGGGTTATTCTTGTTCTCTGGTCAAATTATATAGTTGGAAAACAAGGATGCATATATGAATGAACGATGCAGAGGCAATGCCGATGGCGATAGTGGGTATCATGTAGCCGCTTATGCTGGAAAGAAGCAATAACCCGCAGAAAAACAAAGCTCCAAGCTCAACAAAACTAAGGGCATAGACAATAACTACCGATGTCATATACCCATACTCTCTAATCTTGGCCAGTCGGCGCGTTCTGCTTCCGATTAGAAACGTCAAGGCAGCAATCAGGATTGCAATCATGGTTCCTGCATATGATGACAATGTCGCCCCAAGACCATCTCTATGAGCTGAAAAAGAAACACCAGGAATGTAGTGGCGGAAAAGGAGATAGCAAATGCTTACGATAACGTAAGGAATTATTACTATGTAAACACCAGGCATGATTCTGTTCCGCATAATTACTCCTGATAATTAATCCTTAACTTTGCCCACCTGCCTTTTAAAACATTCCAGTATATCACTTTTCATTCTTGCGTAGCAATATGCCATCTCTTCAGCTATCTCAGCATTGGTGACCTTGTTCAGAGGCGCTGAGAGATGGCCTTTTTCTGATAGATAATGTTCTGTTAAAATATCTCCGGCCTCATCTTTTGCCCGCAGGCTAATGTCTGAAAATTGAGGTGACGGGTTAAAAATAATATCCTTGGCAACCTTTTTTATATCCCTTTTAAATTTTGGCTTAATGACTATATCCAATGAGTCAAAAAGCTCCCCTTCAATATCTGTTGCCCCTAAGACCTTTAATATATCGCCAAATACAGGTAGCTTGGCTTCTACCTTCACCGTTGTTCGGCCGATGAAATGCATATGCATAACATCGTCTTTGGTGGTTCCCCTCATCAGTGGCTCTATCTGAACGCGCTCTCCACTGCTTAATGACATTCCTTTCCCGATTAAAAAATCTGTCAGATCGGATGTGGTCGGCCCGAAAACAGTTCTGGCAAAACCAATGGTGTCGCCTTCAACAAACAAAAAAGATGGGAATCCCAATGATTCGTCATCTGCGAGGCTGTTCTTAATATCTTCAACTGAAGCTTTAGAGCGATTTATCTTCTGAACCAGACTCTTGTCATTTGTTTTGGTAAAGAGAAAAGTTTTTCCATCGATTTTATGAATATACAAATAATTGGAGCCAACCTGCAGGTGATGATTATCAGCCAGCAGAGAATTAAGGAAAACAGACAGGTTTATTGAGCGCTTATCTTTCCCTTTATTTTTGCTGCGGTAAGTCGCATAAAAACCATTCTTCATAATTCAATCCATTTACTATGTTATGTTCTGAGGGGAGTGAAAATTCCCCTAATTCGATGAAGATTCTTGCTCAATTGTTATCAGCTATGCGCCGACCAGAACACCTTGCCGATCAGCCAAACGTCTCTTCAGGCCACTGACTAGCGATAACTTTCCCCACAACGGAACAACTCTCATTGCATGGGATCATTGGGTACTGTGGGTTTAGTGGTTGTAAAAACACCTGACCGCTATCCCTGATCAGTTTCTTGAAGGTAAACTCATCACCCCCAAGTCTGGCTATGCAGAAATCACCTGGCTCAACAGCCTGCTCAGGGTCAACGAGAATTAACATTCCGTCAGGAAAGCTTGGCTTGGAGCCTGTTGGTGCGGTCATGGAATTACCTTCAACCTCAAGCCAGAATGCAGAATCACTGGCTTTTTTGGTTGTGCTTACCCATCTCTCCGCATCACCTTTGGTAAAGGTTCTAAGCTTAGGTGAGAACATCCCTGCCTGAACATGAGAAAAAACAGGGTACTCATACTCACTTCTAAGTGACGGCTGCATACTAACCGCTTCATACATCTCGTAGATTTCTCTGGCGATTGAAGGGCTAAATTCTTCAACGCTAACTTTGAGAATTTTTGTAAGCAATGCGGCGTTATAAGCATTTAATGCATTGATGCCATTAAATAAAGCACCAACGCCTGACTGCCCCATCCCCATCTTGTCTGCGACAGATTCCTGGGATAAGCCAAGTTCATTTTTCTTTTTTTCATAAATTGCTTTAAGGCGACGTGCGTCCTCAAGCTGCTCTTGTGTTAATGGTTTCTTTTTTGTGCTCATACGTTAAATCTATCACCGCAAGGGATAAATATCTAACACCGTGCGTGTTGACTATTTTACCTCTGGCGGTGATAATGGTTGCATGTACTAAGGAGGTTGTATGGAACAACGCATAACCCTGAAAGATTATGCAATGCGCTTTGGGCAAACCAAGACAGCTAAAGATCTCGGCGTATATCAAAGCGCGATCAACAAGGCCATTCATGCAGGCCGAAAGATTTTTTTAACTATAAACGCTGATGGAAGCGTTTATGCGGAAGAGGTAAAGCCCTTCCCGAGTAACAAAAAAACAACAGCATAAATAACCCCGCTCTTACACATTCCAGCCCTGAAAAAGGGCATCAAATTAAACCACACCTATGGTGTATGCATTTATTTGCATACATTCAATCAATTGTTATCTAAGGAAATACTTACATATGGTTCGTGCAAACAAACGCAACGAGGCTCTACGAATCGAGAGTGCGTTGCTTAACAAAATCGCAATGCTTGGAACTGAGAAGACAGCGGAAGCTGTGGGCGTTGATAAGTCGCGAATTCGTCGTAGAATTCAACGTGGAATTCCTATCGGAATTCTCGGATGAATTCGTCGACAAGCTTCCGGCCGCAGTCGAGCACCACCACCACCACCACTGAGATCCGGCTGCTAACAAAGCCCGAAAGGAAGCTGAGTTGGCTGCTGCCACCGCTGAGCAATAACTAGCATAACCCCTTGGGGCCTCTAAACGGGTCTTGAGGGGTTTTTTGCTGAAAGGAGGAACTATATCCGGATTGGCGAATGGGACGCGCCCTGTAGCGGCGCATTAAGCGCGGCGGGTGTGGTGGTTACGCGCAGCGTGACCGCTACACTTGCCAGCGCCCTAGCGCCCGCTCCTTTCGCTTTCTTCCCTTCCTTTCTCGCCACGTTCGCCGGCTTTCCCCGTCAAGCTCTAAATCGGGGGCTCCCTTTAGGGTTCCGATTTAGTGCTTTACGGCACCTCGACCCCAAAAAACTTGATTAGGGTGATGGTTCACGTAGTGGGCCATCGCCCTGATAGACGGTTTTTCGCCCTTTGACGTTGGAGTCCACGTTCTTTAATAGTGGACTCTTGTTCCAAACTGGAACAACACTCAACCCTATCTCGGTCTATTCTTTTGATTTATAAGGGATTTTGCCGATTTCGGCCTATTGGTTAAAAAATGAGCTGATTTAACAAAAATTTAACGCGAATTTTAACAAAATATTAACGCTTACAATTTAGGTGGCACTTTTCGGGGAAATGTGCGCGGAACCCCTATTTGTTTATTTTTCTAAATACATTCAAATATGTATCCGCTCATGAATTAATTCTTAGAAAAACTCATCGAGCATCAAATGAAACTGCAATTTATTCATATCAGGATTATCAATACCATATTTTTGAAAAAGCCGTTTCTGTAATGAAGGAGAAAACTCACCGAGGCAGTTCCATAGGATGGCAAGATCCTGGTATCGGTCTGCGATTCCGACTCGTCCAACATCAATACAACCTATTAATTTCCCCTCGTCAAAAATAAGGTTATCAAGTGAGAAATCACCATGAGTGACGACTGAATCCGGTGAGAATGGCAAAAGTTTATGCATTTCTTTCCAGACTTGTTCAACAGGCCAGCCATTACGCTCGTCATCAAAATCACTCGCATCAACCAAACCGTTATTCATTCGTGATTGCGCCTGAGCGAGACGAAATACGCGATCGCTGTTAAAAGGACAATTACAAACAGGAATCGAATGCAACCGGCGCAGGAACACTGCCAGCGCATCAACAATATTTTCACCTGAATCAGGATATTCTTCTAATACCTGGAATGCTGTTTTCCCGGGGATCGCAGTGGTGAGTAACCATGCATCATCAGGAGTACGGATAAAATGCTTGATGGTCGGAAGAGGCATAAATTCCGTCAGCCAGTTTAGTCTGACCATCTCATCTGTAACATCATTGGCAACGCTACCTTTGCCATGTTTCAGAAACAACTCTGGCGCATCGGGCTTCCCATACAATCGATAGATTGTCGCACCTGATTGCCCGACATTATCGCGAGCCCATTTATACCCATATAAATCAGCATCCATGTTGGAATTTAATCGCGGCCTAGAGCAAGACGTTTCCCGTTGAATATGGCTCATAACACCCCTTGTATTACTGTTTATGTAAGCAGACAGTTTTATTGTTCATGACCAAAATCCCTTAACGTGAGTTTTCGTTCCACTGAGCGTCAGACCCCGTAGAAAAGATCAAAGGATCTTCTTGAGATCCTTTTTTTCTGCGCGTAATCTGCTGCTTGCAAACAAAAAAACCACCGCTACCAGCGGTGGTTTGTTTGCCGGATCAAGAGCTACCAACTCTTTTTCCGAAGGTAACTGGCTTCAGCAGAGCGCAGATACCAAATACTGTCCTTCTAGTGTAGCCGTAGTTAGGCCACCACTTCAAGAACTCTGTAGCACCGCCTACATACCTCGCTCTGCTAATCCTGTTACCAGTGGCTGCTGCCAGTGGCGATAAGTCGTGTCTTACCGGGTTGGACTCAAGACGATAGTTACCGGATAAGGCGCAGCGGTCGGGCTGAACGGGGGGTTCGTGCACACAGCCCAGCTTGGAGCGAACGACCTACACCGAACTGAGATACCTACAGCGTGAGCTATGAGAAAGCGCCACGCTTCCCGAAGGGAGAAAGGCGGACAGGTATCCGGTAAGCGGCAGGGTCGGAACAGGAGAGCGCACGAGGGAGCTTCCAGGGGGAAACGCCTGGTATCTTTATAGTCCTGTCGGGTTTCGCCACCTCTGACTTGAGCGTCGATTTTTGTGATGCTCGTCAGGGGGGCGGAGCCTATGGAAAAACGCCAGCAACGCGGCCTTTTTACGGTTCCTGGCCTTTTGCTGGCCTTTTGCTCACATGTTCTTTCCTGCGTTATCCCCTGATTCTGTGGATAACCGTATTACCGCCTTTGAGTGAGCTGATACCGCTCGCCGCAGCCGAACGACCGAGCGCAGCGAGTCAGTGAGCGAGGAAGCGGAAGAGCGCCTGATGCGGTATTTTCTCCTTACGCATCTGTGCGGTATTTCACACCGCATATATGGTGCACTCTCAGTACAATCTGCTCTGATGCCGCATAGTTAAGCCAGTATACACTCCGCTATCGCTACGTGACTGGGTCATGGCTGCGCCCCGACACCCGCCAACACCCGCTGACGCGCCCTGACGGGCTTGTCTGCTCCCGGCATCCGCTTACAGACAAGCTGTGACCGTCTCCGGGAGCTGCATGTGTCAGAGGTTTTCACCGTCATCACCGAAACGCGCGAGGCAGCTGCGGTAAAGCTCATCAGCGTGGTCGTGAAGCGATTCACAGATGTCTGCCTGTTCATCCGCGTCCAGCTCGTTGAGTTTCTCCAGAAGCGTTAATGTCTGGCTTCTGATAAAGCGGGCCATGTTAAGGGCGGTTTTTTCCTGTTTGGTCACTGATGCCTCCGTGTAAGGGGGATTTCTGTTCATGGGGGTAATGATACCGATGAAACGAGAGAGGATGCTCACGATACGGGTTACTGATGATGAACATGCCCGGTTACTGGAACGTTGTGAGGGTAAACAACTGGCGGTATGGATGCGGCGGGACCAGAGAAAAATCACTCAGGGTCAATGCCAGCGCTTCGTTAATACAGATGTAGGTGTTCCACAGGGTAGCCAGCAGCATCCTGCGATGCAGATCCGGAACATAATGGTGCAGGGCGCTGACTTCCGCGTTTCCAGACTTTACGAAACACGGAAACCGAAGACCATTCATGTTGTTGCTCAGGTCGCAGACGTTTTGCAGCAGCAGTCGCTTCACGTTCGCTCGCGTATCGGTGATTCATTCTGCTAACCAGTAAGGCAACCCCGCCAGCCTAGCCGGGTCCTCAACGACAGGAGCACGATCATGCGCACCCGTGGGGCCGCCATGCCGGCGCCTGCCACCATACCCACGCCGAAACAAGCGCTCATGAGCCCGAAGTGGCGAGCCCGATCTTCCCCATCGGTGATGTCGGCGATATAGGCGCCAGCAACCGCACCTGTGGCGCCGGTGATGCCGGCCACGATGCGTCCGGCGTAGAGGATCGAGATCTCGATCCCGCGAAATTAATACGACTCACTATAGGGGAATTGTGAGCGGATAACAATTCCCTTCTAGCCATATTGGACTCGGACCTGTTTCACGTGGAACACTGAGCCTGGACTAGGTCTAGCATATTGGACTCGACTTTCACGTGGAACACTGAGCCTGGACTAGGTCTAGAACAGGTTCTTTTTTCTTTGTTTCACGTGGAACATTCTGATTAATGTACAGCTAGCCATATTGGACTCGGACCTGTTTCACGTGGAACACTGAGCCTGGACTAGGTCTAGAACAGGTTCTTTTTTCTTTGTTTCACGTGGAACATTCTGATTAATGTACAGCTAGAACAGGTTCTTTTTTCTTTGTTTCACGTGGAACATTCTGATTAATGTACAGCTAGAAATAATTTTGTTTAACTTTAAGAAGGAGATATACCATGTGTTTCACGTGGAACACATGGCTAAAGGCCTTGGAAAAGGGATTAATGCGTTATTTAATCAGGTAGATTTGTCTGAAGAGACAGTTGAAGAAATTAAAATTGCCGATTTACGCCCTAATCCTTATCAGCCAAGAAAACACTTTGATGACGAGGCATTAGCTGAACTAAAAGAATCTGTGCTGCAGCATGGCATTCTTCAGCCGCTTATCGTCAGAAAATCTTTAAAAGGCTATGATATTGTTGCGGGTGAACGGCGTTTTCGAGCGGCAAAGCTGGCAGGTTTAGATACAGTTCCGGCCATTGTCCGTGAATTATCAGAGGCGTTAATGAGGGAAATTGCTTTATTAGAAAACCTTCAGCGTGAAGATTTATCTCCGCTTGAAGAGGCTCAGGCATATGCCGAAACAAGCGCTCATGAGCCCGAAGTGGCGAGCCCGATCTTCCCCATCGGTGATGTCGGCGATATAGGCGCCAGCAACCGCACCTGTGGCGCCGGTGATGCCGGCCACGATGCGTCCGGCGTAGAGGATCGAGATCTCGATCCCGCGAAATTAATACGACTCACTATAGGGGAATTGTGAGCGGATAACAATTCCCTTCTAGCCATATTGGACTCGGACCTGTTTCACGTGGAACACTGAGCCTGGACTAGGTCTAGCATATTGGACTCGACTTTCACGTGGAACACTGAGCCTGGACTAGGTCTAGAACAGGTTCTTTTTTCTTTGTTTCACGTGGAACATTCTGATTAATGTACAGCTAGCCATATTGGACTCGGACCTGTTTCACGTGGAACACTGAGCCTGGACTAGGTCTAGAACAGGTTCTTTTTTCTTTGTTTCACGTGGAACATTCTGATTAATGTACAGCTAGAACAGGTTCTTTTTTCTTTGTTTCACGTGGAACATTCTGATTAATGTACAGCTAGAAATAATTTTGTTTAACTTTAAGAAGGAGATATACCATGTGTTTCACGTGGAACACATGGCTAAAGGCCTTGGAAAAGGGATTAATGCGCATATGACTCCCTTTTGAAACACTTAGATCTCACACAAGAGCAGCTTGCCAAACGTCTTGGGAAAAGCAGACCGCATATTGCGAATCATTTAAGACTGCTGACACTGCCAGAAAATATTCAACAGCTTATTGCCGAAGGCACGCTTTCTATGGGACATGGACGCACGCTTCTTGGCTTAAAAAACAAAAATAAGCTTGAACCGCTGGTACAAAAAGTGATTGCGGAGCAGCTCAATGTTCGCCAACTTGAGCAGCTGATTCAGCAGTTGAATCAGAATGTTCCACGTGAAACAAAGAAAAAAGAACCTGTGAAAGATGCGGTTCGCCATGCCGGCGCCTGCCACCATACCCACGCCGAAACAAGCGCTCATGAGCCCGAAGTGGCGAGCCCGATCTTCCCCATCGGTGATGTCGGCGATATAGGCGCCAGCAACCGCACCTGTGGCGCCGGTGATGCCGGCCACGATGCGTCCGGCGTAGAGGATCGAGATCTCGATCCCGCGAAATTAATACGACTCACTATAGGGGAATTGTGAGCGGATAACAATTCCCTTCTAGCCATATTGGACTCGGACCTGTTTCACGTGGAACACTGAGCCTGGACTAGGTCTAGCATATTGGACTCGACTTTCACGTGGAACACTGAGCCTGGACTAGGTCTAGAACAGGTTCTTTTTTCTTTGTTTCACGTGGAACATTCTGATTAATGTACAGCTAGCCATATTGGACTCGGACCTGTTTCACGTGGAACACTGAGCCTGGACTAGGTCTAGAACAGGTTCTTTTTTCTTTGTTTCACGTGGAACATTCTGATTAATGTACAGCTAGAACAGGTTCTTTTTTCTTTGTTTCACGTGGAACATTCTGATTAATGTACAGCTAGAAATAATTTTGTTTAACTTTAAGAAGGAGATATACCATGTGTTTCACGTGGAACACATGGCTAAAGGCCTTGGAAAAGGGATTAATGCGTTATTTAATCAGGTAGATTTGTCTGAAGAGACAGTTGAAGAAATTAAAATTGCCGATTTACGCCCTAATCCTTATCAGCCAAGAAAACACTTTGATGACGAGGCATTAGCTGAACTAAAAGAATCTGTGCTGCAGCATGGCATTCTTCAGCCGCTTATCGTCAGAAAATCTTTAAAAGGCTATGATATTGTTGCGGGTGAACGGCGTTTTCGAGCGGCAAAGCTGGCAGGTTTAGATACAGTTCCGGCCATTGTCCGTGAATTATCAGAGGCGTTAATGAGGGAAATTGCTTTATTAGAAAACCTTCAGCGTGAAGATTTATCTCCGCTTGAAGAGGCTCAGGCATATGCCGAAACAAGCGCTCATGAGCCCGAAGTGGCGAGCCCGATCTTCCCCATCGGTGATGTCGGCGATATAGGCGCCAGCAACCGCACCTGTGGCGCCGGTGATGCCGGCCACGATGCGTCCGGCGTAGAGGATCGAGATCTCGATCCCGCGAAATTAATACGACTCACTATAGGGGAATTGTGAGCGGATAACAATTCCCTTCTAGCCATATTGGACTCGGACCTGTTTCACGTGGAACACTGAGCCTGGACTAGGTCTAGCATATTGGACTCGACTTTCACGTGGAACACTGAGCCTGGACTAGGTCTAGAACAGGTTCTTTTTTCTTTGTTTCACGTGGAACATTCTGATTAATGTACAGCTAGCCATATTGGACTCGGACCTGTTTCACGTGGAACACTGAGCCTGGACTAGGTCTAGAACAGGTTCTTTTTTCTTTGTTTCACGTGGAACATTCTGATTAATGTACAGCTAGAACAGGTTCTTTTTTCTTTGTTTCACGTGGAACATTCTGATTAATGTACAGCTAGAAATAATTTTGTTTAACTTTAAGAAGGAGATATACCATGTGTTTCACGTGGAACACATGGCTAAAGGCCTTGGAAAAGGGATTAATGCGCATATGACTCCCTTTTGAAACACTTAGATCTCACACAAGAGCAGCTTGCCAAACGTCTTGGGAAAAGCAGACCGCATATTGCGAATCATTTAAGACTGCTGACACTGCCAGAAAATATTCAACAGCTTATTGCCGAAGGCACGCTTTCTATGGGACATGGACGCACGCTTCTTGGCTTAAAAAACAAAAATAAGCTTGAACCGCTGGTACAAAAAGTGATTGCGGAGCAGCTCAATGTTCGCCAACTTGAGCAGCTGATTCAGCAGTTGAATCAGAATGTTCCACGTGAAACAAAGAAAAAAGAACCTGTGAAAGATGCGGTTCGCCATGCCGGCGATAATGGCCTGCTTCTCGCCGAAACGTTTGGTGGCGGGACCAGTGACGAAGGCTTGAGCGAGGGCGTGCAAGATTCCGAATACCGCAAGCGACAGGCCGATCATCGTCGCGCTCCAGCGAAAGCGGTCCTCGCCGAAAATGACCCAGAGCGCTGCCGGCACCTGTCCTACGAGTTGCATGATAAAGAAGACAGTCATAAGTGCGGCGACGATAGTCATGCCCCGCGCCCACCGGAAGGAGCTGACTGGGTTGAAGGCTCTCAAGGGCATCGGTCGAGATCCCGGTGCCTAATGAGTGAGCTAACTTACATTAATTGCGTTGCGCTCACTGCCCGCTTTCCAGTCGGGAAACCTGTCGTGCCAGCTGCATTAATGAATCGGCCAACGCGCGGGGAGAGGCGGTTTGCGTATTGGGCGCCAGGGTGGTTTTTCTTTTCACCAGTGAGACGGGCAACAGCTGATTGCCCTTCACCGCCTGGCCCTGAGAGAGTTGCAGCAAGCGGTCCACGCTGGTTTGCCCCAGCAGGCGAAAATCCTGTTTGATGGTGGTTAACGGCGGGATATAACATGAGCTGTCTTCGGTATCGTCGTATCCCACTACCGAGATATCCGCACCAACGCGCAGCCCGGACTCGGTAATGGCGCGCATTGCGCCCAGCGCCATCTGATCGTTGGCAACCAGCATCGCAGTGGGAACGATGCCCTCATTCAGCATTTGCATGGTTTGTTGAAAACCGGACATGGCACTCCAGTCGCCTTCCCGTTCCGCTATCGGCTGAATTTGATTGCGAGTGAGATATTTATGCCAGCCAGCCAGACGCAGACGCGCCGAGACAGAACTTAATGGGCCCGCTAACAGCGCGATTTGCTGGTGACCCAATGCGACCAGATGCTCCACGCCCAGTCGCGTACCGTCTTCATGGGAGAAAATAATACTGTTGATGGGTGTCTGGTCAGAGACATCAAGAAATAACGCCGGAACATTAGTGCAGGCAGCTTCCACAGCAATGGCATCCTGGTCATCCAGCGGATAGTTAATGATCAGCCCACTGACGCGTTGCGCGAGAAGATTGTGCACCGCCGCTTTACAGGCTTCGACGCCGCTTCGTTCTACCATCGACACCACCACGCTGGCACCCAGTTGATCGGCGCGAGATTTAATCGCCGCGACAATTTGCGACGGCGCGTGCAGGGCCAGACTGGAGGTGGCAACGCCAATCAGCAACGACTGTTTGCCCGCCAGTTGTTGTGCCACGCGGTTGGGAATGTAATTCAGCTCCGCCATCGCCGCTTCCACTTTTTCCCGCGTTTTCGCAGAAACGTGGCTGGCCTGGTTCACCACGCGGGAAACGGTCTGATAAGAGACACCGGCATACTCTGCGACATCGTATAACGTTACTGGTTTCACATTCACCACCCTGAATTGACTCTCTTCCGGGCGCTATCATGCCATACCGCGAAAGGTTTTGCGCCATTCGATGGTGTCCGGGATCTCGACGCTCTCCCTTATGCGACTCCTGCATTAGGAAGCAGCCCAGTAGTAGGTTGAGGCCGTTGAGCACCGCCGCCGCAAGGAATGGTGCATGCAAGGAGATGGCGCCCAACAGTCCCCCGGCCACGGGGCCTGCCACCATACCCACGCCGAAACAAGCGCTCATGAGCCCGAAGTGGCGAGCCCGATCTTCCCCATCGGTGATGTCGGCGATATAGGCGCCAGCAACCGCACCTGTGGCGCCGGTGATGCCGGCCACGATGCGTCCGGCGTAGAGGATCGAGATCTCGATCCCGCGAAATTAATACGACTCACTATAGGGGAATTGTGAGCGGATAACAATTCCCTTCTAGCCATATTGGACTCGGACCTGTTTCACGTGGAACACTGAGCCTGGACTAGGTCTAGCATATTGGACTCGACTTTCACGTGGAACACTGAGCCTGGACTAGGTCTAGAACAGGTTCTTTTTTCTTTGTTTCACGTGGAACATTCTGATTAATGTACAGCTAGCCATATTGGACTCGGACCTGTTTCACGTGGAACACTGAGCCTGGACTAGGTCTAGAACAGGTTCTTTTTTCTTTGTTTCACGTGGAACATTCTGATTAATGTACAGCTAGAACAGGTTCTTTTTTCTTTGTTTCACGTGGAACATTCTGATTAATGTACAGCTAGAAATAATTTTGTTTAACTTTAAGAAGGAGATATACCATGTGTTTCACGTGGAACACATGGCTAAAGGCCTTGGAAAAGGGATTAATGCGTTATTTAATCAGGTAGATTTGTCTGAAGAGACAGTTGAAGAAATTAAAATTGCCGATTTACGCCCTAATCCTTATCAGCCAAGAAAACACTTTGATGACGAGGCATTAGCTGAACTAAAAGAATCTGTGCTGCAGCATGGCATTCTTCAGCCGCTTATCGTCAGAAAATCTTTAAAAGGCTATGATATTGTTGCGGGTGAACGGCGTTTTCGAGCGGCAAAGCTGGCAGGTTTAGATACAGTTCCGGCCATTGTCCGTGAATTATCAGAGGCGTTAATGAGGGAAATTGCTTTATTAGAAAACCTTCAGCGTGAAGATTTATCTCCGCTTGAAGAGGCTCAGGCATATGCCGAAACAAGCGCTCATGAGCCCGAAGTGGCGAGCCCGATCTTCCCCATCGGTGATGTCGGCGATATAGGCGCCAGCAACCGCACCTGTGGCGCCGGTGATGCCGGCCACGATGCGTCCGGCGTAGAGGATCGAGATCTCGATCCCGCGAAATTAATACGACTCACTATAGGGGAATTGTGAGCGGATAACAATTCCCTTCTAGCCATATTGGACTCGGACCTGTTTCACGTGGAACACTGAGCCTGGACTAGGTCTAGCATATTGGACTCGACTTTCACGTGGAACACTGAGCCTGGACTAGGTCTAGAACAGGTTCTTTTTTCTTTGTTTCACGTGGAACATTCTGATTAATGTACAGCTAGCCATATTGGACTCGGACCTGTTTCACGTGGAACACTGAGCCTGGACTAGGTCTAGAACAGGTTCTTTTTTCTTTGTTTCACGTGGAACATTCTGATTAATGTACAGCTAGAACAGGTTCTTTTTTCTTTGTTTCACGTGGAACATTCTGATTAATGTACAGCTAGAAATAATTTTGTTTAACTTTAAGAAGGAGATATACCATGTGTTTCACGTGGAACACATGGCTAAAGGCCTTGGAAAAGGGATTAATGCGCATATGACTCCCTTTTGAAACACTTAGATCTCACACAAGAGCAGCTTGCCAAACGTCTTGGGAAAAGCAGACCGCATATTGCGAATCATTTAAGACTGCTGACACTGCCAGAAAATATTCAACAGCTTATTGCCGAAGGCACGCTTTCTATGGGACATGGACGCACGCTTCTTGGCTTAAAAAACAAAAATAAGCTTGAACCGCTGGTACAAAAAGTGATTGCGGAGCAGCTCAATGTTCGCCAACTTGAGCAGCTGATTCAGCAGTTGAATCAGAATGTTCCACGTGAAACAAAGAAAAAAGAACCTGTGAAAGATGCGGTTCTAAAAGAACGGGAATCCTATCTCCAAAATTATTTTGGAACAACAGTTAATATTAAAAGACAGAAGAAAAAAGGCAAAATCGAAATTGAATTTTTCTCTAATGAAGACCTTGACCGGATTTTAGAGCTTTTGTCTGAACGAGAATCATAAGGATCCGAATTCATCCGAGAATTCCGATAGGAATTCCACGTTGAATTCTACGACGAATTCTCAACTGTGAGGAGGCCACGGTACTGGAGTCGTTTCTGGAAGAGCACGGGGGCTGGAAATCCTTTCTGTGGACGCCGCCTTATGAGTGGCGGCAGATAAAGGTGACCTGCGCAAAATGGTCGTCGCGGGTCAGTATGCTGCGTGTTGAGTTCAGCGCAGAGTTTGAACAGGTGGTGAACTGATGCAGGATATCCGGCAGGAAACACTGAATGAATGCACCCGTGCGGAGCAGTCGGCCAGCGTGGTGCTCTGGGAAATCGACCTGACAGAGGTCGGTGGAGAACGTTATTTTTTCTGTAATGAGCAGAACGAAAAAGGTGAGCCGGTCACCTGGCAGGGGCGACAGTATCAGCCGTATCCCATTCAGGGGAGCGGTTTTGAACTGAATGGCAAAGGCACCAGTACGCGCCCCACGCTGACGGTTTCTAACCTGTACGGTATGGTCACCGGGATGGCGGAAGATATGCAGAGTCTGGTCGGCGGAACGGTGGTCCGGCGTAAGGTTTACGCCCGTTTTCTGGATGCGGTGAACTTCGTCAACGGAAACAGTTACGCCGATCCGGAGCAGGAGGTGATCAGCCGCTGGCGCATTGAGCAGTGCAGCGAACTGAGCGCGGTGAGTGCCTCCTTTGTACTGTCCACGCCGACGGAAACGGATGGCGCTGTTTTTCCGGGACGTATCATGCTGGCCAACACCTGCACCTGGACCTATCGCGGTGACGAGTGCGGTTATAGCGGTCCGGCTGTCGCGGATGAATATGACCAGCCAACGTCCGATATCACGAAGGATAAATGCAGCAAATGCCTGAGCGGTTGTAAGTTCCGCAATAACGTCGGCAACTTTGGCGGCTTCCTTTCCATTAACAAACTTTCGCAGTAAATCCCATGACACAGACAGAATCAGCGATTCTGGCGCACGCCCGGCGATGTGCGCCAGCGGAGTCGTGCGGCTTCGTGGTAAGCACGCCGGAGGGGGAAAGATATTTCCCCTGCGTGAATATCTCCGGTGAGCCGGAGGCTATTTCCGTATGTCGCCGGAAGACTGGCTGCAGGCAGAAATGCAGGGTGAGATTGTGGCGCTGGTCCACAGCCACCCCGGTGGTCTGCCCTGGCTGAGTGAGGCCGACCGGCGGCTGCAGGTGCAGAGTGATTTGCCGTGGTGGCTGGTCTGCCGGGGGACGATTCATAAGTTCCGCTGTGTGCCGCATCTCACCGGGCGGCGCTTTGAGCACGGTGTGACGGACTGTTACACACTGTTCCGGGC

*C-trap 2x parS DNA (20147 bp)*

GGCCGCGGTGTGCTCCTTATTTATACATAACGAAAAACGCCTCGAGTGAAGCGTTATTGGTATGCGGTAAAACCGCACTCAGGCGGCCTTGATAGTCATATCATCTGAATCAAATATTCCTGATGTATCGATATCGGTAATTCTTATTCCTTCGCTACCATCCATTGGAGGCCATCCTTCCTGACCATTTCCATCATTCCAGTCGAACTCACACACAACACCATATGCATTTAAGTCGCTTGAAATTGCTATAAGCAGAGCATGTTGCGCCAGCATGATTAATACAGCATTTAATACAGAGCCGTGTTTATTGAGTCGGTATTCAGAGTCTGACCAGAAATTATTAATCTGGTGAAGTTTTTCCTCTGTCATTACGTCATGGTCGATTTCAATTTCTATTGATGCTTTCCAGTCGTAATCAATGATGTATTTTTTGATGTTTGACATCTGTTCATATCCTCACAGATAAAAAATCGCCCTCACACTGGAGGGCAAAGAAGATTTCCAATAATCAGAACAAGTCGGCTCCTGTTTAGTTACGAGCGACATTGCTCCGTGTATTCACTCGTTGGAATGAATACACAGTGCAGTGTTTATTCTGTTATTTATGCCAAAAATAAAGGCCACTATCAGGCAGCTTTGTTGTTCTGTTTACCAAGTTCTCTGGCAATCATTGCCGTCGTTCGTATTGCCCATTTATCGACATATTTCCCATCTTCCATTACAGGAAACATTTCTTCAGGCTTAACCATGCATTCCGATTGCAGCTTGCATCCATTGCATCGCTTGAATTGTCCACACCATTGATTTTTATCAATAGTCGTAGTCATACGGATAGTCCTGGTATTGTTCCATCACATCCTGAGGATGCTCTTCGAACTCTTCAAATTCTTCTTCCATATATCACCTTAAATAGTGGATTGCGGTAGTAAAGATTGTGCCTGTCTTTTAACCACATCAGGCTCGGTGGTTCTCGTGTACCCCTACAGCGAGAAATCGGATAAACTATTACAACCCCTACAGTTTGATGAGTATAGAAATGGATCCACTCGTTATTCTCGGACGAGTGTTCAGTAATGAACCTCTGGAGAGAACCATGTATATGATCGTTATCTGGGTTGGACTTCTGCTTTTAAGCCCAGATAACTGGCCTGAATATGTTAATGAGAGAATCGGTATTCCTCATGTGTGGCATGTTTTCGTCTTTGCTCTTGCATTTTCGCTAGCAATTAATGTGCATCGATTATCAGCTATTGCCAGCGCCAGATATAAGCGATTTAAGCTAAGAAAACGCATTAAGATGCAAAACGATAAAGTGCGATCAGTAATTCAAAACCTTACAGAAGAGCAATCTATGGTTTTGTGCGCAGCCCTTAATGAAGGCAGGAAGTATGTGGTTACATCAAAACAATTCCCATACATTAGTGAGTTGATTGAGCTTGGTGTGTTGAACAAAACTTTTTCCCGATGGAATGGAAAGCATATATTATTCCCTATTGAGGATATTTACTGGACTGAATTAGTTGCCAGCTATGATCCATATAATATTGAGATAAAGCCAAGGCCAATATCTAAGTAACTAGATAAGAGGAATCGATTTTCCCTTAATTTTCTGGCGTCCACTGCATGTTATGCCGCGTTCGCCAGGCTTGCTGTACCATGTGCGCTGATTCTTGCGCTCAATACGTTGCAGGTTGCTTTCAATCTGTTTGTGGTATTCAGCCAGCACTGTAAGGTCTATCGGATTTAGTGCGCTTTCTACTCGTGATTTCGGTTTGCGATTCAGCGAGAGAATAGGGCGGTTAACTGGTTTTGCGCTTACCCCAACCAACAGGGGATTTGCTGCTTTCCATTGAGCCTGTTTCTCTGCGCGACGTTCGCGGCGGCGTGTTTGTGCATCCATCTGGATTCTCCTGTCAGTTAGCTTTGGTGGTGTGTGGCAGTTGTAGTCCTGAACGAAAACCCCCCGCGATTGGCACATTGGCAGCTAATCCGGAATCGCACTTACGGCCAATGCTTCGTTTCGTATCACACACCCCAAAGCCTTCTGCTTTGAATGCTGCCCTTCTTCAGGGCTTAATTTTTAAGAGCGTCACCTTCATGGTGGTCAGTGCGTCCTGCTGATGTGCTCAGTATCACCGCCAGTGGTATTTATGTCAACACCGCCAGAGATAATTTATCACCGCAGATGGTTATCTGTATGTTTTTTATATGAATTTATTTTTTGCAGGGGGGCATTGTTTGGTAGGTGAGAGATCTGAATTGCTATGTTTAGTGAGTTGTATCTATTTATTTTTCAATAAATACAATTGGTTATGTGTTTTGGGGGCGATCGTGAGGCAAAGAAAACCCGGCGCTGAGGCCGGGTTATTCTTGTTCTCTGGTCAAATTATATAGTTGGAAAACAAGGATGCATATATGAATGAACGATGCAGAGGCAATGCCGATGGCGATAGTGGGTATCATGTAGCCGCTTATGCTGGAAAGAAGCAATAACCCGCAGAAAAACAAAGCTCCAAGCTCAACAAAACTAAGGGCATAGACAATAACTACCGATGTCATATACCCATACTCTCTAATCTTGGCCAGTCGGCGCGTTCTGCTTCCGATTAGAAACGTCAAGGCAGCAATCAGGATTGCAATCATGGTTCCTGCATATGATGACAATGTCGCCCCAAGACCATCTCTATGAGCTGAAAAAGAAACACCAGGAATGTAGTGGCGGAAAAGGAGATAGCAAATGCTTACGATAACGTAAGGAATTATTACTATGTAAACACCAGGCATGATTCTGTTCCGCATAATTACTCCTGATAATTAATCCTTAACTTTGCCCACCTGCCTTTTAAAACATTCCAGTATATCACTTTTCATTCTTGCGTAGCAATATGCCATCTCTTCAGCTATCTCAGCATTGGTGACCTTGTTCAGAGGCGCTGAGAGATGGCCTTTTTCTGATAGATAATGTTCTGTTAAAATATCTCCGGCCTCATCTTTTGCCCGCAGGCTAATGTCTGAAAATTGAGGTGACGGGTTAAAAATAATATCCTTGGCAACCTTTTTTATATCCCTTTTAAATTTTGGCTTAATGACTATATCCAATGAGTCAAAAAGCTCCCCTTCAATATCTGTTGCCCCTAAGACCTTTAATATATCGCCAAATACAGGTAGCTTGGCTTCTACCTTCACCGTTGTTCGGCCGATGAAATGCATATGCATAACATCGTCTTTGGTGGTTCCCCTCATCAGTGGCTCTATCTGAACGCGCTCTCCACTGCTTAATGACATTCCTTTCCCGATTAAAAAATCTGTCAGATCGGATGTGGTCGGCCCGAAAACAGTTCTGGCAAAACCAATGGTGTCGCCTTCAACAAACAAAAAAGATGGGAATCCCAATGATTCGTCATCTGCGAGGCTGTTCTTAATATCTTCAACTGAAGCTTTAGAGCGATTTATCTTCTGAACCAGACTCTTGTCATTTGTTTTGGTAAAGAGAAAAGTTTTTCCATCGATTTTATGAATATACAAATAATTGGAGCCAACCTGCAGGTGATGATTATCAGCCAGCAGAGAATTAAGGAAAACAGACAGGTTTATTGAGCGCTTATCTTTCCCTTTATTTTTGCTGCGGTAAGTCGCATAAAAACCATTCTTCATAATTCAATCCATTTACTATGTTATGTTCTGAGGGGAGTGAAAATTCCCCTAATTCGATGAAGATTCTTGCTCAATTGTTATCAGCTATGCGCCGACCAGAACACCTTGCCGATCAGCCAAACGTCTCTTCAGGCCACTGACTAGCGATAACTTTCCCCACAACGGAACAACTCTCATTGCATGGGATCATTGGGTACTGTGGGTTTAGTGGTTGTAAAAACACCTGACCGCTATCCCTGATCAGTTTCTTGAAGGTAAACTCATCACCCCCAAGTCTGGCTATGCAGAAATCACCTGGCTCAACAGCCTGCTCAGGGTCAACGAGAATTAACATTCCGTCAGGAAAGCTTGGCTTGGAGCCTGTTGGTGCGGTCATGGAATTACCTTCAACCTCAAGCCAGAATGCAGAATCACTGGCTTTTTTGGTTGTGCTTACCCATCTCTCCGCATCACCTTTGGTAAAGGTTCTAAGCTTAGGTGAGAACATCCCTGCCTGAACATGAGAAAAAACAGGGTACTCATACTCACTTCTAAGTGACGGCTGCATACTAACCGCTTCATACATCTCGTAGATTTCTCTGGCGATTGAAGGGCTAAATTCTTCAACGCTAACTTTGAGAATTTTTGTAAGCAATGCGGCGTTATAAGCATTTAATGCATTGATGCCATTAAATAAAGCACCAACGCCTGACTGCCCCATCCCCATCTTGTCTGCGACAGATTCCTGGGATAAGCCAAGTTCATTTTTCTTTTTTTCATAAATTGCTTTAAGGCGACGTGCGTCCTCAAGCTGCTCTTGTGTTAATGGTTTCTTTTTTGTGCTCATACGTTAAATCTATCACCGCAAGGGATAAATATCTAACACCGTGCGTGTTGACTATTTTACCTCTGGCGGTGATAATGGTTGCATGTACTAAGGAGGTTGTATGGAACAACGCATAACCCTGAAAGATTATGCAATGCGCTTTGGGCAAACCAAGACAGCTAAAGATCTCGGCGTATATCAAAGCGCGATCAACAAGGCCATTCATGCAGGCCGAAAGATTTTTTTAACTATAAACGCTGATGGAAGCGTTTATGCGGAAGAGGTAAAGCCCTTCCCGAGTAACAAAAAAACAACAGCATAAATAACCCCGCTCTTACACATTCCAGCCCTGAAAAAGGGCATCAAATTAAACCACACCTATGGTGTATGCATTTATTTGCATACATTCAATCAATTGTTATCTAAGGAAATACTTACATATGGTTCGTGCAAACAAACGCAACGAGGCTCTACGAATCGAGAGTGCGTTGCTTAACAAAATCGCAATGCTTGGAACTGAGAAGACAGCGGAAGCTGTGGGCGTTGATAAGTCGCGTCGACAAGCTTCCGGCCGCAGTCGAGCACCACCACCACCACCACTGAGATCCGGCTGCTAACAAAGCCCGAAAGGAAGCTGAGTTGGCTGCTGCCACCGCTGAGCAATAACTAGCATAACCCCTTGGGGCCTCTAAACGGGTCTTGAGGGGTTTTTTGCTGAAAGGAGGAACTATATCCGGATTGGCGAATGGGACGCGCCCTGTAGCGGCGCATTAAGCGCGGCGGGTGTGGTGGTTACGCGCAGCGTGACCGCTACACTTGCCAGCGCCCTAGCGCCCGCTCCTTTCGCTTTCTTCCCTTCCTTTCTCGCCACGTTCGCCGGCTTTCCCCGTCAAGCTCTAAATCGGGGGCTCCCTTTAGGGTTCCGATTTAGTGCTTTACGGCACCTCGACCCCAAAAAACTTGATTAGGGTGATGGTTCACGTAGTGGGCCATCGCCCTGATAGACGGTTTTTCGCCCTTTGACGTTGGAGTCCACGTTCTTTAATAGTGGACTCTTGTTCCAAACTGGAACAACACTCAACCCTATCTCGGTCTATTCTTTTGATTTATAAGGGATTTTGCCGATTTCGGCCTATTGGTTAAAAAATGAGCTGATTTAACAAAAATTTAACGCGAATTTTAACAAAATATTAACGTTTACAATTTCAGGTGGCACTTTTCGGGGAAATGTGCGCGGAACCCCTATTTGTTTATTTTTCTAAATACATTCAAATATGTATCCGCTCATGAATTAATTCTTAGAAAAACTCATCGAGCATCAAATGAAACTGCAATTTATTCATATCAGGATTATCAATACCATATTTTTGAAAAAGCCGTTTCTGTAATGAAGGAGAAAACTCACCGAGGCAGTTCCATAGGATGGCAAGATCCTGGTATCGGTCTGCGATTCCGACTCGTCCAACATCAATACAACCTATTAATTTCCCCTCGTCAAAAATAAGGTTATCAAGTGAGAAATCACCATGAGTGACGACTGAATCCGGTGAGAATGGCAAAAGTTTATGCATTTCTTTCCAGACTTGTTCAACAGGCCAGCCATTACGCTCGTCATCAAAATCACTCGCATCAACCAAACCGTTATTCATTCGTGATTGCGCCTGAGCGAGACGAAATACGCGATCGCTGTTAAAAGGACAATTACAAACAGGAATCGAATGCAACCGGCGCAGGAACACTGCCAGCGCATCAACAATATTTTCACCTGAATCAGGATATTCTTCTAATACCTGGAATGCTGTTTTCCCGGGGATCGCAGTGGTGAGTAACCATGCATCATCAGGAGTACGGATAAAATGCTTGATGGTCGGAAGAGGCATAAATTCCGTCAGCCAGTTTAGTCTGACCATCTCATCTGTAACATCATTGGCAACGCTACCTTTGCCATGTTTCAGAAACAACTCTGGCGCATCGGGCTTCCCATACAATCGATAGATTGTCGCACCTGATTGCCCGACATTATCGCGAGCCCATTTATACCCATATAAATCAGCATCCATGTTGGAATTTAATCGCGGCCTAGAGCAAGACGTTTCCCGTTGAATATGGCTCATAACACCCCTTGTATTACTGTTTATGTAAGCAGACAGTTTTATTGTTCATGACCAAAATCCCTTAACGTGAGTTTTCGTTCCACTGAGCGTCAGACCCCGTAGAAAAGATCAAAGGATCTTCTTGAGATCCTTTTTTTCTGCGCGTAATCTGCTGCTTGCAAACAAAAAAACCACCGCTACCAGCGGTGGTTTGTTTGCCGGATCAAGAGCTACCAACTCTTTTTCCGAAGGTAACTGGCTTCAGCAGAGCGCAGATACCAAATACTGTCCTTCTAGTGTAGCCGTAGTTAGGCCACCACTTCAAGAACTCTGTAGCACCGCCTACATACCTCGCTCTGCTAATCCTGTTACCAGTGGCTGCTGCCAGTGGCGATAAGTCGTGTCTTACCGGGTTGGACTCAAGACGATAGTTACCGGATAAGGCGCAGCGGTCGGGCTGAACGGGGGGTTCGTGCACACAGCCCAGCTTGGAGCGAACGACCTACACCGAACTGAGATACCTACAGCGTGAGCTATGAGAAAGCGCCACGCTTCCCGAAGGGAGAAAGGCGGACAGGTATCCGGTAAGCGGCAGGGTCGGAACAGGAGAGCGCACGAGGGAGCTTCCAGGGGGAAACGCCTGGTATCTTTATAGTCCTGTCGGGTTTCGCCACCTCTGACTTGAGCGTCGATTTTTGTGATGCTCGTCAGGGGGGCGGAGCCTATGGAAAAACGCCAGCAACGCGGCCTTTTTACGGTTCCTGGCCTTTTGCTGGCCTTTTGCTCACATGTTCTTTCCTGCGTTATCCCCTGATTCTGTGGATAACCGTATTACCGCCTTTGAGTGAGCTGATACCGCTCGCCGCAGCCGAACGACCGAGCGCAGCGAGTCAGTGAGCGAGGAAGCGGAAGAGCGCCTGATGCGGTATTTTCTCCTTACGCATCTGTGCGGTATTTCACACCGCATATATGGTGCACTCTCAGTACAATCTGCTCTGATGCCGCATAGTTAAGCCAGTATACACTCCGCTATCGCTACGTGACTGGGTCATGGCTGCGCCCCGACACCCGCCAACACCCGCTGACGCGCCCTGACGGGCTTGTCTGCTCCCGGCATCCGCTTACAGACAAGCTGTGACCGTCTCCGGGAGCTGCATGTGTCAGAGGTTTTCACCGTCATCACCGAAACGCGCGAGGCAGCTGCGGTAAAGCTCATCAGCGTGGTCGTGAAGCGATTCACAGATGTCTGCCTGTTCATCCGCGTCCAGCTCGTTGAGTTTCTCCAGAAGCGTTAATGTCTGGCTTCTGATAAAGCGGGCCATGTTAAGGGCGGTTTTTTCCTGTTTGGTCACTGATGCCTCCGTGTAAGGGGGATTTCTGTTCATGGGGGTAATGATACCGATGAAACGAGAGAGGATGCTCACGATACGGGTTACTGATGATGAACATGCCCGGTTACTGGAACGTTGTGAGGGTAAACAACTGGCGGTATGGATGCGGCGGGACCAGAGAAAAATCACTCAGGGTCAATGCCAGCGCTTCGTTAATACAGATGTAGGTGTTCCACAGGGTAGCCAGCAGCATCCTGCGATGCAGATCCGGAACATAATGGTGCAGGGCGCTGACTTCCGCGTTTCCAGACTTTACGAAACACGGAAACCGAAGACCATTCATGTTGTTGCTCAGGTCGCAGACGTTTTGCAGCAGCAGTCGCTTCACGTTCGCTCGCGTATCGGTGATTCATTCTGCTAACCAGTAAGGCAACCCCGCCAGCCTAGCCGGGTCCTCAACGACAGGAGCACGATCATGCGCACCCGTGGGGCCGCCATGCCGGCGATAATGGCCTGCTTCTCGCCGAAACGTTTGGTGGCGGGACCAGTGACGAAGGCTTGAGCGAGGGCGTGCAAGATTCCGAATACCGCAAGCGACAGGCCGATCATCGTCGCGCTCCAGCGAAAGCGGTCCTCGCCGAAAATGACCCAGAGCGCTGCCGGCACCTGTCCTACGAGTTGCATGATAAAGAAGACAGTCATAAGTGCGGCGACGATAGTCATGCCCCGCGCCCACCGGAAGGAGCTGACTGGGTTGAAGGCTCTCAAGGGCATCGGTCGAGATCCCGGTGCCTAATGAGTGAGCTAACTTACATTAATTGCGTTGCGCTCACTGCCCGCTTTCCAGTCGGGAAACCTGTCGTGCCAGCTGCATTAATGAATCGGCCAACGCGCGGGGAGAGGCGGTTTGCGTATTGGGCGCCAGGGTGGTTTTTCTTTTCACCAGTGAGACGGGCAACAGCTGATTGCCCTTCACCGCCTGGCCCTGAGAGAGTTGCAGCAAGCGGTCCACGCTGGTTTGCCCCAGCAGGCGAAAATCCTGTTTGATGGTGGTTAACGGCGGGATATAACATGAGCTGTCTTCGGTATCGTCGTATCCCACTACCGAGATATCCGCACCAACGCGCAGCCCGGACTCGGTAATGGCGCGCATTGCGCCCAGCGCCATCTGATCGTTGGCAACCAGCATCGCAGTGGGAACGATGCCCTCATTCAGCATTTGCATGGTTTGTTGAAAACCGGACATGGCACTCCAGTCGCCTTCCCGTTCCGCTATCGGCTGAATTTGATTGCGAGTGAGATATTTATGCCAGCCAGCCAGACGCAGACGCGCCGAGACAGAACTTAATGGGCCCGCTAACAGCGCGATTTGCTGGTGACCCAATGCGACCAGATGCTCCACGCCCAGTCGCGTACCGTCTTCATGGGAGAAAATAATACTGTTGATGGGTGTCTGGTCAGAGACATCAAGAAATAACGCCGGAACATTAGTGCAGGCAGCTTCCACAGCAATGGCATCCTGGTCATCCAGCGGATAGTTAATGATCAGCCCACTGACGCGTTGCGCGAGAAGATTGTGCACCGCCGCTTTACAGGCTTCGACGCCGCTTCGTTCTACCATCGACACCACCACGCTGGCACCCAGTTGATCGGCGCGAGATTTAATCGCCGCGACAATTTGCGACGGCGCGTGCAGGGCCAGACTGGAGGTGGCAACGCCAATCAGCAACGACTGTTTGCCCGCCAGTTGTTGTGCCACGCGGTTGGGAATGTAATTCAGCTCCGCCATCGCCGCTTCCACTTTTTCCCGCGTTTTCGCAGAAACGTGGCTGGCCTGGTTCACCACGCGGGAAACGGTCTGATAAGAGACACCGGCATACTCTGCGACATCGTATAACGTTACTGGTTTCACATTCACCACCCTGAATTGACTCTCTTCCGGGCGCTATCATGCCATACCGCGAAAGGTTTTGCGCCATTCGATGGTGTCCGGGATCTCGACGCTCTCCCTTATGCGACTCCTGCATTAGGAAGCAGCCCAGTAGTAGGTTGAGGCCGTTGAGCACCGCCGCCGCAAGGAATGGTGCATGCAAGGAGATGGCGCCCAACAGTCCCCCGGCCACGGGGCCTGCCACCATACCCACGCCGAAACAAGCGCTCATGAGCCCGAAGTGGCGAGCCCGATCTTCCCCATCGGTGATGTCGGCGATATAGGCGCCAGCAACCGCACCTGTGGCGCCGGTGATGCCGGCCACGATGCGTCCGGCGTAGAGGATCGAGATCTCGATCCCGCGAAATTAATACGACTCACTATAGGGGAATTGTGAGCGGATAACAATTCCCCTCTAGAAATAATTTTGTTTAACTTTAAGAAGGAGATATACCATGTGTTTCACGTGGAACACATGGCTAAAGGCCTTGGAAAAGGGATTAATGCGTTATTTAATCAGGTAGATTTGTCTGAAGAGACAGTTGAAGAAATTAAAATTGCCGATTTACGCCCTAATCCTTATCAGCCAAGAAAACACTTTGATGACGAGGCATTAGCTGAACTAAAAGAATCTGTGCTGCAGCATGGCATTCTTCAGCCGCTTATCGTCAGAAAATCTTTAAAAGGCTATGATATTGTTGCGGGTGAACGGCGTTTTCGAGCGGCAAAGCTGGCAGGTTTAGATACAGTTCCGGCCATTGTCCGTGAATTATCAGAGGCGTTAATGAGGGAAATTGCTTTATTAGAAAACCTTCAGCGTGAAGATTTATCTCCGCTTGAAGAGGCTCAGGCATATGACTCCCTTTTGAAACACTTAGATCTCACACAAGAGCAGCTTGCCAAACGTCTTGGGAAAAGCAGACCGCATATTGCGAATCATTTAAGACTGCTGACACTGCCAGAAAATATTCAACAGCTTATTGCCGAAGGCACGCTTTCTATGGGACATGGACGCACGCTTCTTGGCTTAAAAAACAAAAATAAGCTTGAACCGCTGGTACAAAAAGTGATTGCGGAGCAGCTCAATGTTCGCCAACTTGAGCAGCTGATTCAGCAGTTGAATCAGAATGTTCCACGTGAAACAAAGAAAAAAGAACCTGTGAAAGATGCGGTTCTAAAAGAACGGGAATCCTATCTCCAAAATTATTTTGGAACAACAGTTAATATTAAAAGACAGAAGAAAAAAGGCAAAATCGAAATTGAATTTTTCTCTAATGAAGACCTTGACCGGATTTTAGAGCTTTTGTCTGAACGAGAATCATAAGGATCCTCAACTGTGAGGAGGCTCACGGACGCGAAGAACAGGCACGCGTGCTGGCAGAAACCCCCGGTATGACCGTGAAAACGGCCCGCCGCATTCTGGCCGCAGCACCACAGAGTGCACAGGCGCGCAGTGACACTGCGCTGGATCGTCTGATGCAGGGGGCACCGGCACCGCTGGCTGCAGGTAACCCGGCATCTGATGCCGTTAACGATTTGCTGAACACACCAGTGTAAGGGATGTTTATGACGAGCAAAGAAACCTTTACCCATTACCAGCCGCAGGGCAACAGTGACCCGGCTCATACCGCAACCGCGCCCGGCGGATTGAGTGCGAAAGCGCCTGCAATGACCCCGCTGATGCTGGACACCTCCAGCCGTAAGCTGGTTGCGTGGGATGGCACCACCGACGGTGCTGCCGTTGGCATTCTTGCGGTTGCTGCTGACCAGACCAGCACCACGCTGACGTTCTACAAGTCCGGCACGTTCCGTTATGAGGATGTGCTCTGGCCGGAGGCTGCCAGCGACGAGACGAAAAAACGGACCGCGTTTGCCGGAACGGCAATCAGCATCGTTTAACTTTACCCTTCATCACTAAAGGCCGCCTGTGCGGCTTTTTTTACGGGATTTTTTTATGTCGATGTACACAACCGCCCAACTGCTGGCGGCAAATGAGCAGAAATTTAAGTTTGATCCGCTGTTTCTGCGTCTCTTTTTCCGTGAGAGCTATCCCTTCACCACGGAGAAAGTCTATCTCTCACAAATTCCGGGACTGGTAAACATGGCGCTGTACGTTTCGCCGATTGTTTCCGGTGAGGTTATCCGTTCCCGTGGCGGCTCCACCTCTGAATTTACGCCGGGATATGTCAAGCCGAAGCATGAAGTGAATCCGCAGATGACCCTGCGTCGCCTGCCGGATGAAGATCCGCAGAATCTGGCGGACCCGGCTTACCGCCGCCGTCGCATCATCATGCAGAACATGCGTGACGAAGAGCTGGCCATTGCTCAGGTCGAAGAGATGCAGGCAGTTTCTGCCGTGCTTAAGGGCAAATACACCATGACCGGTGAAGCCTTCGATCCGGTTGAGGTGGATATGGGCCGCAGTGAGGAGAATAACATCACGCAGTCCGGCGGCACGGAGTGGAGCAAGCGTGACAAGTCCACGTATGACCCGACCGACGATATCGAAGCCTACGCGCTGAACGCCAGCGGTGTGGTGAATATCATCGTGTTCGATCCGAAAGGCTGGGCGCTGTTCCGTTCCTTCAAAGCCGTCAAGGAGAAGCTGGATACCCGTCGTGGCTCTAATTCCGAGCTGGAGACAGCGGTGAAAGACCTGGGCAAAGCGGTGTCCTATAAGGGGATGTATGGCGATGTGGCCATCGTCGTGTATTCCGGACAGTACGTGGAAAACGGCGTCAAAAAGAACTTCCTGCCGGACAACACGATGGTGCTGGGGAACACTCAGGCACGCGGTCTGCGCACCTATGGCTGCATTCAGGATGCGGACGCACAGCGCGAAGGCATTAACGCCTCTGCCCGTTACCCGAAAAACTGGGTGACCACCGGCGATCCGGCGCGTGAGTTCACCATGATTCAGTCAGCACCGCTGATGCTGCTGGCTGACCCTGATGAGTTCGTGTCCGTACAACTGGCGTAATCATGGCCCTTCGGGGCCATTGTTTCTCTGTGGAGGAGTCCATGACGAAAGATGAACTGATTGCCCGTCTCCGCTCGCTGGGTGAACAACTGAACCGTGATGTCAGCCTGACGGGGACGAAAGAAGAACTGGCGCTCCGTGTGGCAGAGCTGAAAGAGGAGCTTGATGACACGGATGAAACTGCCGGTCAGGACACCCCTCTCAGCCGGGAAAATGTGCTGACCGGACATGAAAATGAGGTGGGATCAGCGCAGCCGGATACCGTGATTCTGGATACGTCTGAACTGGTCACGGTCGTGGCACTGGTGAAGCTGCATACTGATGCACTTCACGCCACGCGGGATGAACCTGTGGCATTTGTGCTGCCGGGAACGGCGTTTCGTGTCTCTGCCGGTGTGGCAGCCGAAATGACAGAGCGCGGCCTGGCCAGAATGCAATAACGGGAGGCGCTGTGGCTGATTTCGATAACCTGTTCGATGCTGCCATTGCCCGCGCCGATGAAACGATACGCGGGTACATGGGAACGTCAGCCACCATTACATCCGGTGAGCAGTCAGGTGCGGTGATACGTGGTGTTTTTGATGACCCTGAAAATATCAGCTATGCCGGACAGGGCGTGCGCGTTGAAGGCTCCAGCCCGTCCCTGTTTGTCCGGACTGATGAGGTGCGGCAGCTGCGGCGTGGAGACACGCTGACCATCGGTGAGGAAAATTTCTGGGTAGATCGGGTTTCGCCGGATGATGGCGGAAGTTGTCATCTCTGGCTTGGACGGGGCGTACCGCCTGCCGTTAACCGTCGCCGCTGAAAGGGGGATGTATGGCCATAAAAGGTCTTGAGCAGGCCGTTGAAAACCTCAGCCGTATCAGCAAAACGGCGGTGCCTGGTGCCGCCGCAATGGCCATTAACCGCGTTGCTTCATCCGCGATATCGCAGTCGGCGTCACAGGTTGCCCGTGAGACAAAGGTACGCCGGAAACTGGTAAAGGAAAGGGCCAGGCTGAAAAGGGCCACGGTCAAAAATCCGCAGGCCAGAATCAAAGTTAACCGGGGGGATTTGCCCGTAATCAAGCTGGGTAATGCGCGGGTTGTCCTTTCGCGCCGCAGGCGTCGTAAAAAGGGGCAGCGTTCATCCCTGAAAGGTGGCGGCAGCGTGCTTGTGGTGGGTAACCGTCGTATTCCCGGCGCGTTTATTCAGCAACTGAAAAATGGCCGGTGGCATGTCATGCAGCGTGTGGCTGGGAAAAACCGTTACCCCATTGATGTGGTGAAAATCCCGATGGCGGTGCCGCTGACCACGGCGTTTAAACAAAATATTGAGCGGATACGGCGTGAACGTCTTCCGAAAGAGCTGGGCTATGCGCTGCAGCATCAACTGAGGATGGTAATAAAGCGATGAAACATACTGAACTCCGTGCAGCCGTACTGGATGCACTGGAGAAGCATGACACCGGGGCGACGTTTTTTGATGGTCGCCCCGCTGTTTTTGATGAGGCGGATTTTCCGGCAGTTGCCGTTTATCTCACCGGCGCTGAATACACGGGCGAAGAGCTGGACAGCGATACCTGGCAGGCGGAGCTGCATATCGAAGTTTTCCTGCCTGCTCAGGTGCCGGATTCAGAGCTGGATGCGTGGATGGAGTCCCGGATTTATCCGGTGATGAGCGATATCCCGGCACTGTCAGATTTGATCACCAGTATGGTGGCCAGCGGCTATGACTACCGGCGCGACGATGATGCGGGCTTGTGGAGTTCAGCCGATCTGACTTATGTCATTACCTATGAAATGTGAGGACGCTATGCCTGTACCAAATCCTACAATGCCGGTGAAAGGTGCCGGGACCACCCTGTGGGTTTATAAGGGGAGCGGTGACCCTTACGCGAATCCGCTTTCAGACGTTGACTGGTCGCGTCTGGCAAAAGTTAAAGACCTGACGCCCGGCGAACTGACCGCTGAGTCCTATGACGACAGCTATCTCGATGATGAAGATGCAGACTGGACTGCGACCGGGCAGGGGCAGAAATCTGCCGGAGATACCAGCTTCACGCTGGCGTGGATGCCCGGAGAGCAGGGGCAGCAGGCGCTGCTGGCGTGGTTTAATGAAGGCGATACCCGTGCCTATAAAATCCGCTTCCCGAACGGCACGGTCGATGTGTTCCGTGGCTGGGTCAGCAGTATCGGTAAGGCGGTGACGGCGAAGGAAGTGATCACCCGCACGGTGAAAGTCACCAATGTGGGACGTCCGTCGATGGCAGAAGATCGCAGCACGGTAACAGCGGCAACCGGCATGACCGTGACGCCTGCCAGCACCTCGGTGGTGAAAGGGCAGAGCACCACGCTGACCGTGGCCTTCCAGCCGGAGGGCGTAACCGACAAGAGCTTTCGTGCGGTGTCTGCGGATAAAACAAAAGCCACCGTGTCGGTCAGTGGTATGACCATCACCGTGAACGGCGTTGCTGCAGGCAAGGTCAACATTCCGGTTGTATCCGGTAATGGTGAGTTTGCTGCGGTTGCAGAAATTACCGTCACCGCCAGTTAATCCGGAGAGTCAGCGATGTTCCTGAAAACCGAATCATTTGAACATAACGGTGTGACCGTCACGCTTTCTGAACTGTCAGCCCTGCAGCGCATTGAGCATCTCGCCCTGATGAAACGGCAGGCAGAACAGGCGGAGTCAGACAGCAACCGGAAGTTTACTGTGGAAGACGCCATCAGAACCGGCGCGTTTCTGGTGGCGATGTCCCTGTGGCATAACCATCCGCAGAAGACGCAGATGCCGTCCATGAATGAAGCCGTTAAACAGATTGAGCAGGAAGTGCTTACCACCTGGCCCACGGAGGCAATTTCTCATGCTGAAAACGTGGTGTACCGGCTGTCTGGTATGTATGAGTTTGTGGTGAATAATGCCCCTGAACAGACAGAGGACGCCGGGCCCGCAGAGCCTGTTTCTGCGGGAAAGTGTTCGACGGTGAGCTGAGTTTTGCCCTGAAACTGGCGCGTGAGATGGGGCGACCCGACTGGCGTGCCATGCTTGCCGGGATGTCATCCACGGAGTATGCCGACTGGCACCGCTTTTACAGTACCCATTATTTTCATGATGTTCTGCTGGATATGCACTTTTCCGGGCTGACGTACACCGTGCTCAGCCTGTTTTTCAGCGATCCGGATATGCATCCGCTGGATTTCAGTCTGCTGAACCGGCGCGAGGCTGACGAAGAGCCTGAAGATGATGTGCTGATGCAGAAAGCGGCAGGGCTTGCCGGAGGTGTCCGCTTTGGCCCGGACGGGAATGAAGTTATCCCCGCTTCCCCGGATGTGGCGGACATGACGGAGGATGACGTAATGCTGATGACAGTATCAGAAGGGATCGCAGGAGGAGTCCGGTATGGCTGAACCGGTAGGCGATCTGGTCGTTGATTTGAGTCTGGATGCGGCCAGATTTGACGAGCAGATGGCCAGAGTCAGGCGTCATTTTTCTGGTACGGAAAGTGATGCGAAAAAAACAGCGGCAGTCGTTGAACAGTCGCTGAGCCGACAGGCGCTGGCTGCACAGAAAGCGGGGATTTCCGTCGGGCAGTATAAAGCCGCCATGCGTATGCTGCCTGCACAGTTCACCGACGTGGCCACGCAGCTTGCAGGCGGGCAAAGTCCGTGGCTGATCCTGCTGCAACAGGGGGGGCAGGTGAAGGACTCCTTCGGCGGGATGATCCCCATGTTCAGGGGGCTTGCCGGTGCGATCACCCTGCCGATGGTGGGGGCCACCTCGCTGGCGGTGGCGACCGGTGCGCTGGCGTATGCCTGGTATCAGGGCAACTCAACCCTGTCCGATTTCAACAAAACGCTGGTCCTTTCCGGCAATCAGGCGGGACTGACGGCAGATCGTATGCTGGTCCTGTCCAGAGCCGGGCAGGCGGCAGGGCTGACGTTTAACCAGACCAGCGAGTCACTCAGCGCACTGGTTAAGGCGGGGGTAAGCGGTGAGGCTCAGATTGCGTCCATCAGCCAGAGTGTGGCGCGTTTCTCCTCTGCATCCGGCGTGGAGGTGGACAAGGTCGCTGAAGCCTTCGGGAAGCTGACCACAGACCCGACGTCGGGGCTGACGGCGATGGCTCGCCAGTTCCATAACGTGTCGGCGGAGCAGATTGCGTATGTTGCTCAGTTGCAGCGTTCCGGCGATGAAGCCGGGGCATTGCAGGCGGCGAACGAGGCCGCAACGAAAGGGTTTGATGACCAGACCCGCCGCCTGAAAGAGAACATGGGCACGCTGGAGACCTGGGCAGACAGGACTGCGCGGGCATTCAAATCCATGTGGGATGCGGTGCTGGATATTGGTCGTCCTGATACCGCGCAGGAGATGCTGATTAAGGCAGAGGCTGCGTATAAGAAAGCAGACGACATCTGGAATCTGCGCAAGGATGATTATTTTGTTAACGATGAAGCGCGGGCGCGTTACTGGGATGATCGTGAAAAGGCCCGTCTTGCGCTTGAAGCCGCCCGAAAGAAGGCTGAGCAGCAGACTCAACAGGACAAAAATGCGCAGCAGCAGAGCGATACCGAAGCGTCACGGCTGAAATATACCGAAGAGGCGCAGAAGGCTTACGAACGGCTGCAGACGCCGCTGGAGAAATATACCGCCCGTCAGGAAGAACTGAACAAGGCACTGAAAGACGGGAAAATCCTGCAGGCGGATTACAACACGCTGATGGCGGCGGCGAAAAAGGATTATGAAGCGACGCTGAAAAAGCCGAAACAGTCCAGCGTGAAGGTGTCTGCGGGCGATCGTCAGGAAGACAGTGCTCATGCTGCCCTGCTGACGCTTCAGGCAGAACTCCGGACGCTGGAGAAGCATGCCGGAGCAAATGAGAAAATCAGCCAGCAGCGCCGGGATTTGTGGAAGGCGGAGAGTCAGTTCGCGGTACTGGAGGAGGCGGCGCAACGTCGCCAGCTGTCTGCACAGGAGAAATCCCTGCTGGCGCATAAAGATGAGACGCTGGAGTACAAACGCCAGCTGGCTGCACTTGGCGACAAGGTTACGTATCAGGAGCGCCTGAACGCGCTGGCGCAGCAGGCGGATAAATTCGCACAGCAGCAACGGGCAAAACGGGCCGCCATTGATGCGAAAAGCCGGGGGCTGACTGACCGGCAGGCAGAACGGGAAGCCACGGAACAGCGCCTGAAGGAACAGTATGGCGATAATCCGCTGGCGCTGAATAACGTCATGTCAGAGCAGAAAAAGACCTGGGCGGCTGAAGACCAGCTTCGCGGGAACTGGATGGCAGGCCTGAAGTCCGGCTGGAGTGAGTGGGAAGAGAGCGCCACGGACAGTATGTCGCAGGTAAAAAGTGCAGCCACGCAGACCTTTGATGGTATTGCACAGAATATGGCGGCGATGCTGACCGGCAGTGAGCAGAACTGGCGCAGCTTCACCCGTTCCGTGCTGTCCATGATGACAGAAATTCTGCTTAAGCAGGCAATGGTGGGGATTGTCGGGAGTATCGGCAGCGCCATTGGCGGGGCTGTTGGTGGCGGCGCATCCGCGTCAGGCGGTACAGCCATTCAGGCCGCTGCGGCGAAATTCCATTTTGCAACCGGAGGATTTACGGGAACCGGCGGCAAATATGAGCCAGCGGGGATTGTTCACCGTGGTGAGTTTGTCTTCACGAAGGAGGCAACCAGCCGGATTGGCGTGGGGAATCTTTACCGGCTGATGCGCGGCTATGCCACCGGCGGTTATGTCGGTACACCGGGCAGCATGGCAGACAGCCGGTCGCAGGCGTCCGGGACGTTTGAGCAGAATAACCATGTGGTGATTAACAACGACGGCACGAACGGGCAGATAGGTCCGGCTGCTCTGAAGGCGGTGTATGACATGGCCCGCAAGGGTGCCCGTGATGAAATTCAGACACAGATGCGTGATGGTGGCCTGTTCTCCGGAGGTGGACGATGAAGACCTTCCGCTGGAAAGTGAAACCCGGTATGGATGTGGCTTCGGTCCCTTCTGTAAGAAAGGTGCGCTTTGGTGATGGCTATTCTCAGCGAGCGCCTGCCGGGCTGAATGCCAACCTGAAAACGTACAGCGTGACGCTTTCTGTCCCCCGTGAGGAGGCCACGGTACTGGAGTCGTTTCTGGAAGAGCACGGGGGCTGGAAATCCTTTCTGTGGACGCCGCCTTATGAGTGGCGGCAGATAAAGGTGACCTGCGCAAAATGGTCGTCGCGGGTCAGTATGCTGCGTGTTGAGTTCAGCGCAGAGTTTGAACAGGTGGTGAACTGATGCAGGATATCCGGCAGGAAACACTGAATGAATGCACCCGTGCGGAGCAGTCGGCCAGCGTGGTGCTCTGGGAAATCGACCTGACAGAGGTCGGTGGAGAACGTTATTTTTTCTGTAATGAGCAGAACGAAAAAGGTGAGCCGGTCACCTGGCAGGGGCGACAGTATCAGCCGTATCCCATTCAGGGGAGCGGTTTTGAACTGAATGGCAAAGGCACCAGTACGCGCCCCACGCTGACGGTTTCTAACCTGTACGGTATGGTCACCGGGATGGCGGAAGATATGCAGAGTCTGGTCGGCGGAACGGTGGTCCGGCGTAAGGTTTACGCCCGTTTTCTGGATGCGGTGAACTTCGTCAACGGAAACAGTTACGCCGATCCGGAGCAGGAGGTGATCAGCCGCTGGCGCATTGAGCAGTGCAGCGAACTGAGCGCGGTGAGTGCCTCCTTTGTACTGTCCACGCCGACGGAAACGGATGGCGCTGTTTTTCCGGGACGTATCATGCTGGCCAACACCTGCACCTGGACCTATCGCGGTGACGAGTGCGGTTATAGCGGTCCGGCTGTCGCGGATGAATATGACCAGCCAACGTCCGATATCACGAAGGATAAATGCAGCAAATGCCTGAGCGGTTGTAAGTTCCGCAATAACGTCGGCAACTTTGGCGGCTTCCTTTCCATTAACAAACTTTCGCAGTAAATCCCATGACACAGACAGAATCAGCGATTCTGGCGCACGCCCGGCGATGTGCGCCAGCGGAGTCGTGCGGCTTCGTGGTAAGCACGCCGGAGGGGGAAAGATATTTCCCCTGCGTGAATATCTCCGGTGAGCCGGAGGCTATTTCCGTATGTCGCCGGAAGACTGGCTGCAGGCAGAAATGCAGGGTGAGATTGTGGCGCTGGTCCACAGCCACCCCGGTGGTCTGCCCTGGCTGAGTGAGGCCGACCGGCGGCTGCAGGTGCAGAGTGATTTGCCGTGGTGGCTGGTCTGCCGGGGGACGATTCATAAGTTCCGCTGTGTGCCGCATCTCACCGGGCGGCGCTTTGAGCACGGTGTGACGGACTGTTACACACTGTTCCGGGC

*C-trap EcoRI 7x parS DNA (20579 bp)*

GGCCGCGGTGTGCTCCTTATTTATACATAACGAAAAACGCCTCGAGTGAAGCGTTATTGGTATGCGGTAAAACCGCACTCAGGCGGCCTTGATAGTCATATCATCTGAATCAAATATTCCTGATGTATCGATATCGGTAATTCTTATTCCTTCGCTACCATCCATTGGAGGCCATCCTTCCTGACCATTTCCATCATTCCAGTCGAACTCACACACAACACCATATGCATTTAAGTCGCTTGAAATTGCTATAAGCAGAGCATGTTGCGCCAGCATGATTAATACAGCATTTAATACAGAGCCGTGTTTATTGAGTCGGTATTCAGAGTCTGACCAGAAATTATTAATCTGGTGAAGTTTTTCCTCTGTCATTACGTCATGGTCGATTTCAATTTCTATTGATGCTTTCCAGTCGTAATCAATGATGTATTTTTTGATGTTTGACATCTGTTCATATCCTCACAGATAAAAAATCGCCCTCACACTGGAGGGCAAAGAAGATTTCCAATAATCAGAACAAGTCGGCTCCTGTTTAGTTACGAGCGACATTGCTCCGTGTATTCACTCGTTGGAATGAATACACAGTGCAGTGTTTATTCTGTTATTTATGCCAAAAATAAAGGCCACTATCAGGCAGCTTTGTTGTTCTGTTTACCAAGTTCTCTGGCAATCATTGCCGTCGTTCGTATTGCCCATTTATCGACATATTTCCCATCTTCCATTACAGGAAACATTTCTTCAGGCTTAACCATGCATTCCGATTGCAGCTTGCATCCATTGCATCGCTTGAATTGTCCACACCATTGATTTTTATCAATAGTCGTAGTCATACGGATAGTCCTGGTATTGTTCCATCACATCCTGAGGATGCTCTTCGAACTCTTCAAATTCTTCTTCCATATATCACCTTAAATAGTGGATTGCGGTAGTAAAGATTGTGCCTGTCTTTTAACCACATCAGGCTCGGTGGTTCTCGTGTACCCCTACAGCGAGAAATCGGATAAACTATTACAACCCCTACAGTTTGATGAGTATAGAAATGGATCCACTCGTTATTCTCGGACGAGTGTTCAGTAATGAACCTCTGGAGAGAACCATGTATATGATCGTTATCTGGGTTGGACTTCTGCTTTTAAGCCCAGATAACTGGCCTGAATATGTTAATGAGAGAATCGGTATTCCTCATGTGTGGCATGTTTTCGTCTTTGCTCTTGCATTTTCGCTAGCAATTAATGTGCATCGATTATCAGCTATTGCCAGCGCCAGATATAAGCGATTTAAGCTAAGAAAACGCATTAAGATGCAAAACGATAAAGTGCGATCAGTAATTCAAAACCTTACAGAAGAGCAATCTATGGTTTTGTGCGCAGCCCTTAATGAAGGCAGGAAGTATGTGGTTACATCAAAACAATTCCCATACATTAGTGAGTTGATTGAGCTTGGTGTGTTGAACAAAACTTTTTCCCGATGGAATGGAAAGCATATATTATTCCCTATTGAGGATATTTACTGGACTGAATTAGTTGCCAGCTATGATCCATATAATATTGAGATAAAGCCAAGGCCAATATCTAAGTAACTAGATAAGAGGAATCGATTTTCCCTTAATTTTCTGGCGTCCACTGCATGTTATGCCGCGTTCGCCAGGCTTGCTGTACCATGTGCGCTGATTCTTGCGCTCAATACGTTGCAGGTTGCTTTCAATCTGTTTGTGGTATTCAGCCAGCACTGTAAGGTCTATCGGATTTAGTGCGCTTTCTACTCGTGATTTCGGTTTGCGATTCAGCGAGAGAATAGGGCGGTTAACTGGTTTTGCGCTTACCCCAACCAACAGGGGATTTGCTGCTTTCCATTGAGCCTGTTTCTCTGCGCGACGTTCGCGGCGGCGTGTTTGTGCATCCATCTGGATTCTCCTGTCAGTTAGCTTTGGTGGTGTGTGGCAGTTGTAGTCCTGAACGAAAACCCCCCGCGATTGGCACATTGGCAGCTAATCCGGAATCGCACTTACGGCCAATGCTTCGTTTCGTATCACACACCCCAAAGCCTTCTGCTTTGAATGCTGCCCTTCTTCAGGGCTTAATTTTTAAGAGCGTCACCTTCATGGTGGTCAGTGCGTCCTGCTGATGTGCTCAGTATCACCGCCAGTGGTATTTATGTCAACACCGCCAGAGATAATTTATCACCGCAGATGGTTATCTGTATGTTTTTTATATGAATTTATTTTTTGCAGGGGGGCATTGTTTGGTAGGTGAGAGATCTGAATTGCTATGTTTAGTGAGTTGTATCTATTTATTTTTCAATAAATACAATTGGTTATGTGTTTTGGGGGCGATCGTGAGGCAAAGAAAACCCGGCGCTGAGGCCGGGTTATTCTTGTTCTCTGGTCAAATTATATAGTTGGAAAACAAGGATGCATATATGAATGAACGATGCAGAGGCAATGCCGATGGCGATAGTGGGTATCATGTAGCCGCTTATGCTGGAAAGAAGCAATAACCCGCAGAAAAACAAAGCTCCAAGCTCAACAAAACTAAGGGCATAGACAATAACTACCGATGTCATATACCCATACTCTCTAATCTTGGCCAGTCGGCGCGTTCTGCTTCCGATTAGAAACGTCAAGGCAGCAATCAGGATTGCAATCATGGTTCCTGCATATGATGACAATGTCGCCCCAAGACCATCTCTATGAGCTGAAAAAGAAACACCAGGAATGTAGTGGCGGAAAAGGAGATAGCAAATGCTTACGATAACGTAAGGAATTATTACTATGTAAACACCAGGCATGATTCTGTTCCGCATAATTACTCCTGATAATTAATCCTTAACTTTGCCCACCTGCCTTTTAAAACATTCCAGTATATCACTTTTCATTCTTGCGTAGCAATATGCCATCTCTTCAGCTATCTCAGCATTGGTGACCTTGTTCAGAGGCGCTGAGAGATGGCCTTTTTCTGATAGATAATGTTCTGTTAAAATATCTCCGGCCTCATCTTTTGCCCGCAGGCTAATGTCTGAAAATTGAGGTGACGGGTTAAAAATAATATCCTTGGCAACCTTTTTTATATCCCTTTTAAATTTTGGCTTAATGACTATATCCAATGAGTCAAAAAGCTCCCCTTCAATATCTGTTGCCCCTAAGACCTTTAATATATCGCCAAATACAGGTAGCTTGGCTTCTACCTTCACCGTTGTTCGGCCGATGAAATGCATATGCATAACATCGTCTTTGGTGGTTCCCCTCATCAGTGGCTCTATCTGAACGCGCTCTCCACTGCTTAATGACATTCCTTTCCCGATTAAAAAATCTGTCAGATCGGATGTGGTCGGCCCGAAAACAGTTCTGGCAAAACCAATGGTGTCGCCTTCAACAAACAAAAAAGATGGGAATCCCAATGATTCGTCATCTGCGAGGCTGTTCTTAATATCTTCAACTGAAGCTTTAGAGCGATTTATCTTCTGAACCAGACTCTTGTCATTTGTTTTGGTAAAGAGAAAAGTTTTTCCATCGATTTTATGAATATACAAATAATTGGAGCCAACCTGCAGGTGATGATTATCAGCCAGCAGAGAATTAAGGAAAACAGACAGGTTTATTGAGCGCTTATCTTTCCCTTTATTTTTGCTGCGGTAAGTCGCATAAAAACCATTCTTCATAATTCAATCCATTTACTATGTTATGTTCTGAGGGGAGTGAAAATTCCCCTAATTCGATGAAGATTCTTGCTCAATTGTTATCAGCTATGCGCCGACCAGAACACCTTGCCGATCAGCCAAACGTCTCTTCAGGCCACTGACTAGCGATAACTTTCCCCACAACGGAACAACTCTCATTGCATGGGATCATTGGGTACTGTGGGTTTAGTGGTTGTAAAAACACCTGACCGCTATCCCTGATCAGTTTCTTGAAGGTAAACTCATCACCCCCAAGTCTGGCTATGCAGAAATCACCTGGCTCAACAGCCTGCTCAGGGTCAACGAGAATTAACATTCCGTCAGGAAAGCTTGGCTTGGAGCCTGTTGGTGCGGTCATGGAATTACCTTCAACCTCAAGCCAGAATGCAGAATCACTGGCTTTTTTGGTTGTGCTTACCCATCTCTCCGCATCACCTTTGGTAAAGGTTCTAAGCTTAGGTGAGAACATCCCTGCCTGAACATGAGAAAAAACAGGGTACTCATACTCACTTCTAAGTGACGGCTGCATACTAACCGCTTCATACATCTCGTAGATTTCTCTGGCGATTGAAGGGCTAAATTCTTCAACGCTAACTTTGAGAATTTTTGTAAGCAATGCGGCGTTATAAGCATTTAATGCATTGATGCCATTAAATAAAGCACCAACGCCTGACTGCCCCATCCCCATCTTGTCTGCGACAGATTCCTGGGATAAGCCAAGTTCATTTTTCTTTTTTTCATAAATTGCTTTAAGGCGACGTGCGTCCTCAAGCTGCTCTTGTGTTAATGGTTTCTTTTTTGTGCTCATACGTTAAATCTATCACCGCAAGGGATAAATATCTAACACCGTGCGTGTTGACTATTTTACCTCTGGCGGTGATAATGGTTGCATGTACTAAGGAGGTTGTATGGAACAACGCATAACCCTGAAAGATTATGCAATGCGCTTTGGGCAAACCAAGACAGCTAAAGATCTCGGCGTATATCAAAGCGCGATCAACAAGGCCATTCATGCAGGCCGAAAGATTTTTTTAACTATAAACGCTGATGGAAGCGTTTATGCGGAAGAGGTAAAGCCCTTCCCGAGTAACAAAAAAACAACAGCATAAATAACCCCGCTCTTACACATTCCAGCCCTGAAAAAGGGCATCAAATTAAACCACACCTATGGTGTATGCATTTATTTGCATACATTCAATCAATTGTTATCTAAGGAAATACTTACATATGGTTCGTGCAAACAAACGCAACGAGGCTCTACGAATCGAGAGTGCGTTGCTTAACAAAATCGCAATGCTTGGAACTGAGAAGACAGCGGAAGCTGTGGGCGTTGATAAGTCGCGAATTCGTCGTAGAATTCAACGTGGAATTCCTATCGGAATTCTCGGATGAATTCGTCGACAAGCTTCCGGCCGCAGTCGAGCACCACCACCACCACCACTGAGATCCGGCTGCTAACAAAGCCCGAAAGGAAGCTGAGTTGGCTGCTGCCACCGCTGAGCAATAACTAGCATAACCCCTTGGGGCCTCTAAACGGGTCTTGAGGGGTTTTTTGCTGAAAGGAGGAACTATATCCGGATTGGCGAATGGGACGCGCCCTGTAGCGGCGCATTAAGCGCGGCGGGTGTGGTGGTTACGCGCAGCGTGACCGCTACACTTGCCAGCGCCCTAGCGCCCGCTCCTTTCGCTTTCTTCCCTTCCTTTCTCGCCACGTTCGCCGGCTTTCCCCGTCAAGCTCTAAATCGGGGGCTCCCTTTAGGGTTCCGATTTAGTGCTTTACGGCACCTCGACCCCAAAAAACTTGATTAGGGTGATGGTTCACGTAGTGGGCCATCGCCCTGATAGACGGTTTTTCGCCCTTTGACGTTGGAGTCCACGTTCTTTAATAGTGGACTCTTGTTCCAAACTGGAACAACACTCAACCCTATCTCGGTCTATTCTTTTGATTTATAAGGGATTTTGCCGATTTCGGCCTATTGGTTAAAAAATGAGCTGATTTAACAAAAATTTAACGCGAATTTTAACAAAATATTAACGCTTACAATTTAGGTGGCACTTTTCGGGGAAATGTGCGCGGAACCCCTATTTGTTTATTTTTCTAAATACATTCAAATATGTATCCGCTCATGAATTAATTCTTAGAAAAACTCATCGAGCATCAAATGAAACTGCAATTTATTCATATCAGGATTATCAATACCATATTTTTGAAAAAGCCGTTTCTGTAATGAAGGAGAAAACTCACCGAGGCAGTTCCATAGGATGGCAAGATCCTGGTATCGGTCTGCGATTCCGACTCGTCCAACATCAATACAACCTATTAATTTCCCCTCGTCAAAAATAAGGTTATCAAGTGAGAAATCACCATGAGTGACGACTGAATCCGGTGAGAATGGCAAAAGTTTATGCATTTCTTTCCAGACTTGTTCAACAGGCCAGCCATTACGCTCGTCATCAAAATCACTCGCATCAACCAAACCGTTATTCATTCGTGATTGCGCCTGAGCGAGACGAAATACGCGATCGCTGTTAAAAGGACAATTACAAACAGGAATCGAATGCAACCGGCGCAGGAACACTGCCAGCGCATCAACAATATTTTCACCTGAATCAGGATATTCTTCTAATACCTGGAATGCTGTTTTCCCGGGGATCGCAGTGGTGAGTAACCATGCATCATCAGGAGTACGGATAAAATGCTTGATGGTCGGAAGAGGCATAAATTCCGTCAGCCAGTTTAGTCTGACCATCTCATCTGTAACATCATTGGCAACGCTACCTTTGCCATGTTTCAGAAACAACTCTGGCGCATCGGGCTTCCCATACAATCGATAGATTGTCGCACCTGATTGCCCGACATTATCGCGAGCCCATTTATACCCATATAAATCAGCATCCATGTTGGAATTTAATCGCGGCCTAGAGCAAGACGTTTCCCGTTGAATATGGCTCATAACACCCCTTGTATTACTGTTTATGTAAGCAGACAGTTTTATTGTTCATGACCAAAATCCCTTAACGTGAGTTTTCGTTCCACTGAGCGTCAGACCCCGTAGAAAAGATCAAAGGATCTTCTTGAGATCCTTTTTTTCTGCGCGTAATCTGCTGCTTGCAAACAAAAAAACCACCGCTACCAGCGGTGGTTTGTTTGCCGGATCAAGAGCTACCAACTCTTTTTCCGAAGGTAACTGGCTTCAGCAGAGCGCAGATACCAAATACTGTCCTTCTAGTGTAGCCGTAGTTAGGCCACCACTTCAAGAACTCTGTAGCACCGCCTACATACCTCGCTCTGCTAATCCTGTTACCAGTGGCTGCTGCCAGTGGCGATAAGTCGTGTCTTACCGGGTTGGACTCAAGACGATAGTTACCGGATAAGGCGCAGCGGTCGGGCTGAACGGGGGGTTCGTGCACACAGCCCAGCTTGGAGCGAACGACCTACACCGAACTGAGATACCTACAGCGTGAGCTATGAGAAAGCGCCACGCTTCCCGAAGGGAGAAAGGCGGACAGGTATCCGGTAAGCGGCAGGGTCGGAACAGGAGAGCGCACGAGGGAGCTTCCAGGGGGAAACGCCTGGTATCTTTATAGTCCTGTCGGGTTTCGCCACCTCTGACTTGAGCGTCGATTTTTGTGATGCTCGTCAGGGGGGCGGAGCCTATGGAAAAACGCCAGCAACGCGGCCTTTTTACGGTTCCTGGCCTTTTGCTGGCCTTTTGCTCACATGTTCTTTCCTGCGTTATCCCCTGATTCTGTGGATAACCGTATTACCGCCTTTGAGTGAGCTGATACCGCTCGCCGCAGCCGAACGACCGAGCGCAGCGAGTCAGTGAGCGAGGAAGCGGAAGAGCGCCTGATGCGGTATTTTCTCCTTACGCATCTGTGCGGTATTTCACACCGCATATATGGTGCACTCTCAGTACAATCTGCTCTGATGCCGCATAGTTAAGCCAGTATACACTCCGCTATCGCTACGTGACTGGGTCATGGCTGCGCCCCGACACCCGCCAACACCCGCTGACGCGCCCTGACGGGCTTGTCTGCTCCCGGCATCCGCTTACAGACAAGCTGTGACCGTCTCCGGGAGCTGCATGTGTCAGAGGTTTTCACCGTCATCACCGAAACGCGCGAGGCAGCTGCGGTAAAGCTCATCAGCGTGGTCGTGAAGCGATTCACAGATGTCTGCCTGTTCATCCGCGTCCAGCTCGTTGAGTTTCTCCAGAAGCGTTAATGTCTGGCTTCTGATAAAGCGGGCCATGTTAAGGGCGGTTTTTTCCTGTTTGGTCACTGATGCCTCCGTGTAAGGGGGATTTCTGTTCATGGGGGTAATGATACCGATGAAACGAGAGAGGATGCTCACGATACGGGTTACTGATGATGAACATGCCCGGTTACTGGAACGTTGTGAGGGTAAACAACTGGCGGTATGGATGCGGCGGGACCAGAGAAAAATCACTCAGGGTCAATGCCAGCGCTTCGTTAATACAGATGTAGGTGTTCCACAGGGTAGCCAGCAGCATCCTGCGATGCAGATCCGGAACATAATGGTGCAGGGCGCTGACTTCCGCGTTTCCAGACTTTACGAAACACGGAAACCGAAGACCATTCATGTTGTTGCTCAGGTCGCAGACGTTTTGCAGCAGCAGTCGCTTCACGTTCGCTCGCGTATCGGTGATTCATTCTGCTAACCAGTAAGGCAACCCCGCCAGCCTAGCCGGGTCCTCAACGACAGGAGCACGATCATGCGCACCCGTGGGGCCGCCATGCCGGCGATAATGGCCTGCTTCTCGCCGAAACGTTTGGTGGCGGGACCAGTGACGAAGGCTTGAGCGAGGGCGTGCAAGATTCCGAATACCGCAAGCGACAGGCCGATCATCGTCGCGCTCCAGCGAAAGCGGTCCTCGCCGAAAATGACCCAGAGCGCTGCCGGCACCTGTCCTACGAGTTGCATGATAAAGAAGACAGTCATAAGTGCGGCGACGATAGTCATGCCCCGCGCCCACCGGAAGGAGCTGACTGGGTTGAAGGCTCTCAAGGGCATCGGTCGAGATCCCGGTGCCTAATGAGTGAGCTAACTTACATTAATTGCGTTGCGCTCACTGCCCGCTTTCCAGTCGGGAAACCTGTCGTGCCAGCTGCATTAATGAATCGGCCAACGCGCGGGGAGAGGCGGTTTGCGTATTGGGCGCCAGGGTGGTTTTTCTTTTCACCAGTGAGACGGGCAACAGCTGATTGCCCTTCACCGCCTGGCCCTGAGAGAGTTGCAGCAAGCGGTCCACGCTGGTTTGCCCCAGCAGGCGAAAATCCTGTTTGATGGTGGTTAACGGCGGGATATAACATGAGCTGTCTTCGGTATCGTCGTATCCCACTACCGAGATATCCGCACCAACGCGCAGCCCGGACTCGGTAATGGCGCGCATTGCGCCCAGCGCCATCTGATCGTTGGCAACCAGCATCGCAGTGGGAACGATGCCCTCATTCAGCATTTGCATGGTTTGTTGAAAACCGGACATGGCACTCCAGTCGCCTTCCCGTTCCGCTATCGGCTGAATTTGATTGCGAGTGAGATATTTATGCCAGCCAGCCAGACGCAGACGCGCCGAGACAGAACTTAATGGGCCCGCTAACAGCGCGATTTGCTGGTGACCCAATGCGACCAGATGCTCCACGCCCAGTCGCGTACCGTCTTCATGGGAGAAAATAATACTGTTGATGGGTGTCTGGTCAGAGACATCAAGAAATAACGCCGGAACATTAGTGCAGGCAGCTTCCACAGCAATGGCATCCTGGTCATCCAGCGGATAGTTAATGATCAGCCCACTGACGCGTTGCGCGAGAAGATTGTGCACCGCCGCTTTACAGGCTTCGACGCCGCTTCGTTCTACCATCGACACCACCACGCTGGCACCCAGTTGATCGGCGCGAGATTTAATCGCCGCGACAATTTGCGACGGCGCGTGCAGGGCCAGACTGGAGGTGGCAACGCCAATCAGCAACGACTGTTTGCCCGCCAGTTGTTGTGCCACGCGGTTGGGAATGTAATTCAGCTCCGCCATCGCCGCTTCCACTTTTTCCCGCGTTTTCGCAGAAACGTGGCTGGCCTGGTTCACCACGCGGGAAACGGTCTGATAAGAGACACCGGCATACTCTGCGACATCGTATAACGTTACTGGTTTCACATTCACCACCCTGAATTGACTCTCTTCCGGGCGCTATCATGCCATACCGCGAAAGGTTTTGCGCCATTCGATGGTGTCCGGGATCTCGACGCTCTCCCTTATGCGACTCCTGCATTAGGAAGCAGCCCAGTAGTAGGTTGAGGCCGTTGAGCACCGCCGCCGCAAGGAATGGTGCATGCAAGGAGATGGCGCCCAACAGTCCCCCGGCCACGGGGCCTGCCACCATACCCACGCCGAAACAAGCGCTCATGAGCCCGAAGTGGCGAGCCCGATCTTCCCCATCGGTGATGTCGGCGATATAGGCGCCAGCAACCGCACCTGTGGCGCCGGTGATGCCGGCCACGATGCGTCCGGCGTAGAGGATCGAGATCTCGATCCCGCGAAATTAATACGACTCACTATAGGGGAATTGTGAGCGGATAACAATTCCCTTCTAGCCATATTGGACTCGGACCTGTTTCACGTGGAACACTGAGCCTGGACTAGGTCTAGCATATTGGACTCGACTTTCACGTGGAACACTGAGCCTGGACTAGGTCTAGAACAGGTTCTTTTTTCTTTGTTTCACGTGGAACATTCTGATTAATGTACAGCTAGCCATATTGGACTCGGACCTGTTTCACGTGGAACACTGAGCCTGGACTAGGTCTAGAACAGGTTCTTTTTTCTTTGTTTCACGTGGAACATTCTGATTAATGTACAGCTAGAACAGGTTCTTTTTTCTTTGTTTCACGTGGAACATTCTGATTAATGTACAGCTAGAAATAATTTTGTTTAACTTTAAGAAGGAGATATACCATGTGTTTCACGTGGAACACATGGCTAAAGGCCTTGGAAAAGGGATTAATGCGTTATTTAATCAGGTAGATTTGTCTGAAGAGACAGTTGAAGAAATTAAAATTGCCGATTTACGCCCTAATCCTTATCAGCCAAGAAAACACTTTGATGACGAGGCATTAGCTGAACTAAAAGAATCTGTGCTGCAGCATGGCATTCTTCAGCCGCTTATCGTCAGAAAATCTTTAAAAGGCTATGATATTGTTGCGGGTGAACGGCGTTTTCGAGCGGCAAAGCTGGCAGGTTTAGATACAGTTCCGGCCATTGTCCGTGAATTATCAGAGGCGTTAATGAGGGAAATTGCTTTATTAGAAAACCTTCAGCGTGAAGATTTATCTCCGCTTGAAGAGGCTCAGGCATATGACTCCCTTTTGAAACACTTAGATCTCACACAAGAGCAGCTTGCCAAACGTCTTGGGAAAAGCAGACCGCATATTGCGAATCATTTAAGACTGCTGACACTGCCAGAAAATATTCAACAGCTTATTGCCGAAGGCACGCTTTCTATGGGACATGGACGCACGCTTCTTGGCTTAAAAAACAAAAATAAGCTTGAACCGCTGGTACAAAAAGTGATTGCGGAGCAGCTCAATGTTCGCCAACTTGAGCAGCTGATTCAGCAGTTGAATCAGAATGTTCCACGTGAAACAAAGAAAAAAGAACCTGTGAAAGATGCGGTTCTAAAAGAACGGGAATCCTATCTCCAAAATTATTTTGGAACAACAGTTAATATTAAAAGACAGAAGAAAAAAGGCAAAATCGAAATTGAATTTTTCTCTAATGAAGACCTTGACCGGATTTTAGAGCTTTTGTCTGAACGAGAATCATAAGGATCCGAATTCATCCGAGAATTCCGATAGGAATTCCACGTTGAATTCTACGACGAATTCTCAACTGTGAGGAGGCTCACGGACGCGAAGAACAGGCACGCGTGCTGGCAGAAACCCCCGGTATGACCGTGAAAACGGCCCGCCGCATTCTGGCCGCAGCACCACAGAGTGCACAGGCGCGCAGTGACACTGCGCTGGATCGTCTGATGCAGGGGGCACCGGCACCGCTGGCTGCAGGTAACCCGGCATCTGATGCCGTTAACGATTTGCTGAACACACCAGTGTAAGGGATGTTTATGACGAGCAAAGAAACCTTTACCCATTACCAGCCGCAGGGCAACAGTGACCCGGCTCATACCGCAACCGCGCCCGGCGGATTGAGTGCGAAAGCGCCTGCAATGACCCCGCTGATGCTGGACACCTCCAGCCGTAAGCTGGTTGCGTGGGATGGCACCACCGACGGTGCTGCCGTTGGCATTCTTGCGGTTGCTGCTGACCAGACCAGCACCACGCTGACGTTCTACAAGTCCGGCACGTTCCGTTATGAGGATGTGCTCTGGCCGGAGGCTGCCAGCGACGAGACGAAAAAACGGACCGCGTTTGCCGGAACGGCAATCAGCATCGTTTAACTTTACCCTTCATCACTAAAGGCCGCCTGTGCGGCTTTTTTTACGGGATTTTTTTATGTCGATGTACACAACCGCCCAACTGCTGGCGGCAAATGAGCAGAAATTTAAGTTTGATCCGCTGTTTCTGCGTCTCTTTTTCCGTGAGAGCTATCCCTTCACCACGGAGAAAGTCTATCTCTCACAAATTCCGGGACTGGTAAACATGGCGCTGTACGTTTCGCCGATTGTTTCCGGTGAGGTTATCCGTTCCCGTGGCGGCTCCACCTCTGAATTTACGCCGGGATATGTCAAGCCGAAGCATGAAGTGAATCCGCAGATGACCCTGCGTCGCCTGCCGGATGAAGATCCGCAGAATCTGGCGGACCCGGCTTACCGCCGCCGTCGCATCATCATGCAGAACATGCGTGACGAAGAGCTGGCCATTGCTCAGGTCGAAGAGATGCAGGCAGTTTCTGCCGTGCTTAAGGGCAAATACACCATGACCGGTGAAGCCTTCGATCCGGTTGAGGTGGATATGGGCCGCAGTGAGGAGAATAACATCACGCAGTCCGGCGGCACGGAGTGGAGCAAGCGTGACAAGTCCACGTATGACCCGACCGACGATATCGAAGCCTACGCGCTGAACGCCAGCGGTGTGGTGAATATCATCGTGTTCGATCCGAAAGGCTGGGCGCTGTTCCGTTCCTTCAAAGCCGTCAAGGAGAAGCTGGATACCCGTCGTGGCTCTAATTCCGAGCTGGAGACAGCGGTGAAAGACCTGGGCAAAGCGGTGTCCTATAAGGGGATGTATGGCGATGTGGCCATCGTCGTGTATTCCGGACAGTACGTGGAAAACGGCGTCAAAAAGAACTTCCTGCCGGACAACACGATGGTGCTGGGGAACACTCAGGCACGCGGTCTGCGCACCTATGGCTGCATTCAGGATGCGGACGCACAGCGCGAAGGCATTAACGCCTCTGCCCGTTACCCGAAAAACTGGGTGACCACCGGCGATCCGGCGCGTGAGTTCACCATGATTCAGTCAGCACCGCTGATGCTGCTGGCTGACCCTGATGAGTTCGTGTCCGTACAACTGGCGTAATCATGGCCCTTCGGGGCCATTGTTTCTCTGTGGAGGAGTCCATGACGAAAGATGAACTGATTGCCCGTCTCCGCTCGCTGGGTGAACAACTGAACCGTGATGTCAGCCTGACGGGGACGAAAGAAGAACTGGCGCTCCGTGTGGCAGAGCTGAAAGAGGAGCTTGATGACACGGATGAAACTGCCGGTCAGGACACCCCTCTCAGCCGGGAAAATGTGCTGACCGGACATGAAAATGAGGTGGGATCAGCGCAGCCGGATACCGTGATTCTGGATACGTCTGAACTGGTCACGGTCGTGGCACTGGTGAAGCTGCATACTGATGCACTTCACGCCACGCGGGATGAACCTGTGGCATTTGTGCTGCCGGGAACGGCGTTTCGTGTCTCTGCCGGTGTGGCAGCCGAAATGACAGAGCGCGGCCTGGCCAGAATGCAATAACGGGAGGCGCTGTGGCTGATTTCGATAACCTGTTCGATGCTGCCATTGCCCGCGCCGATGAAACGATACGCGGGTACATGGGAACGTCAGCCACCATTACATCCGGTGAGCAGTCAGGTGCGGTGATACGTGGTGTTTTTGATGACCCTGAAAATATCAGCTATGCCGGACAGGGCGTGCGCGTTGAAGGCTCCAGCCCGTCCCTGTTTGTCCGGACTGATGAGGTGCGGCAGCTGCGGCGTGGAGACACGCTGACCATCGGTGAGGAAAATTTCTGGGTAGATCGGGTTTCGCCGGATGATGGCGGAAGTTGTCATCTCTGGCTTGGACGGGGCGTACCGCCTGCCGTTAACCGTCGCCGCTGAAAGGGGGATGTATGGCCATAAAAGGTCTTGAGCAGGCCGTTGAAAACCTCAGCCGTATCAGCAAAACGGCGGTGCCTGGTGCCGCCGCAATGGCCATTAACCGCGTTGCTTCATCCGCGATATCGCAGTCGGCGTCACAGGTTGCCCGTGAGACAAAGGTACGCCGGAAACTGGTAAAGGAAAGGGCCAGGCTGAAAAGGGCCACGGTCAAAAATCCGCAGGCCAGAATCAAAGTTAACCGGGGGGATTTGCCCGTAATCAAGCTGGGTAATGCGCGGGTTGTCCTTTCGCGCCGCAGGCGTCGTAAAAAGGGGCAGCGTTCATCCCTGAAAGGTGGCGGCAGCGTGCTTGTGGTGGGTAACCGTCGTATTCCCGGCGCGTTTATTCAGCAACTGAAAAATGGCCGGTGGCATGTCATGCAGCGTGTGGCTGGGAAAAACCGTTACCCCATTGATGTGGTGAAAATCCCGATGGCGGTGCCGCTGACCACGGCGTTTAAACAAAATATTGAGCGGATACGGCGTGAACGTCTTCCGAAAGAGCTGGGCTATGCGCTGCAGCATCAACTGAGGATGGTAATAAAGCGATGAAACATACTGAACTCCGTGCAGCCGTACTGGATGCACTGGAGAAGCATGACACCGGGGCGACGTTTTTTGATGGTCGCCCCGCTGTTTTTGATGAGGCGGATTTTCCGGCAGTTGCCGTTTATCTCACCGGCGCTGAATACACGGGCGAAGAGCTGGACAGCGATACCTGGCAGGCGGAGCTGCATATCGAAGTTTTCCTGCCTGCTCAGGTGCCGGATTCAGAGCTGGATGCGTGGATGGAGTCCCGGATTTATCCGGTGATGAGCGATATCCCGGCACTGTCAGATTTGATCACCAGTATGGTGGCCAGCGGCTATGACTACCGGCGCGACGATGATGCGGGCTTGTGGAGTTCAGCCGATCTGACTTATGTCATTACCTATGAAATGTGAGGACGCTATGCCTGTACCAAATCCTACAATGCCGGTGAAAGGTGCCGGGACCACCCTGTGGGTTTATAAGGGGAGCGGTGACCCTTACGCGAATCCGCTTTCAGACGTTGACTGGTCGCGTCTGGCAAAAGTTAAAGACCTGACGCCCGGCGAACTGACCGCTGAGTCCTATGACGACAGCTATCTCGATGATGAAGATGCAGACTGGACTGCGACCGGGCAGGGGCAGAAATCTGCCGGAGATACCAGCTTCACGCTGGCGTGGATGCCCGGAGAGCAGGGGCAGCAGGCGCTGCTGGCGTGGTTTAATGAAGGCGATACCCGTGCCTATAAAATCCGCTTCCCGAACGGCACGGTCGATGTGTTCCGTGGCTGGGTCAGCAGTATCGGTAAGGCGGTGACGGCGAAGGAAGTGATCACCCGCACGGTGAAAGTCACCAATGTGGGACGTCCGTCGATGGCAGAAGATCGCAGCACGGTAACAGCGGCAACCGGCATGACCGTGACGCCTGCCAGCACCTCGGTGGTGAAAGGGCAGAGCACCACGCTGACCGTGGCCTTCCAGCCGGAGGGCGTAACCGACAAGAGCTTTCGTGCGGTGTCTGCGGATAAAACAAAAGCCACCGTGTCGGTCAGTGGTATGACCATCACCGTGAACGGCGTTGCTGCAGGCAAGGTCAACATTCCGGTTGTATCCGGTAATGGTGAGTTTGCTGCGGTTGCAGAAATTACCGTCACCGCCAGTTAATCCGGAGAGTCAGCGATGTTCCTGAAAACCGAATCATTTGAACATAACGGTGTGACCGTCACGCTTTCTGAACTGTCAGCCCTGCAGCGCATTGAGCATCTCGCCCTGATGAAACGGCAGGCAGAACAGGCGGAGTCAGACAGCAACCGGAAGTTTACTGTGGAAGACGCCATCAGAACCGGCGCGTTTCTGGTGGCGATGTCCCTGTGGCATAACCATCCGCAGAAGACGCAGATGCCGTCCATGAATGAAGCCGTTAAACAGATTGAGCAGGAAGTGCTTACCACCTGGCCCACGGAGGCAATTTCTCATGCTGAAAACGTGGTGTACCGGCTGTCTGGTATGTATGAGTTTGTGGTGAATAATGCCCCTGAACAGACAGAGGACGCCGGGCCCGCAGAGCCTGTTTCTGCGGGAAAGTGTTCGACGGTGAGCTGAGTTTTGCCCTGAAACTGGCGCGTGAGATGGGGCGACCCGACTGGCGTGCCATGCTTGCCGGGATGTCATCCACGGAGTATGCCGACTGGCACCGCTTTTACAGTACCCATTATTTTCATGATGTTCTGCTGGATATGCACTTTTCCGGGCTGACGTACACCGTGCTCAGCCTGTTTTTCAGCGATCCGGATATGCATCCGCTGGATTTCAGTCTGCTGAACCGGCGCGAGGCTGACGAAGAGCCTGAAGATGATGTGCTGATGCAGAAAGCGGCAGGGCTTGCCGGAGGTGTCCGCTTTGGCCCGGACGGGAATGAAGTTATCCCCGCTTCCCCGGATGTGGCGGACATGACGGAGGATGACGTAATGCTGATGACAGTATCAGAAGGGATCGCAGGAGGAGTCCGGTATGGCTGAACCGGTAGGCGATCTGGTCGTTGATTTGAGTCTGGATGCGGCCAGATTTGACGAGCAGATGGCCAGAGTCAGGCGTCATTTTTCTGGTACGGAAAGTGATGCGAAAAAAACAGCGGCAGTCGTTGAACAGTCGCTGAGCCGACAGGCGCTGGCTGCACAGAAAGCGGGGATTTCCGTCGGGCAGTATAAAGCCGCCATGCGTATGCTGCCTGCACAGTTCACCGACGTGGCCACGCAGCTTGCAGGCGGGCAAAGTCCGTGGCTGATCCTGCTGCAACAGGGGGGGCAGGTGAAGGACTCCTTCGGCGGGATGATCCCCATGTTCAGGGGGCTTGCCGGTGCGATCACCCTGCCGATGGTGGGGGCCACCTCGCTGGCGGTGGCGACCGGTGCGCTGGCGTATGCCTGGTATCAGGGCAACTCAACCCTGTCCGATTTCAACAAAACGCTGGTCCTTTCCGGCAATCAGGCGGGACTGACGGCAGATCGTATGCTGGTCCTGTCCAGAGCCGGGCAGGCGGCAGGGCTGACGTTTAACCAGACCAGCGAGTCACTCAGCGCACTGGTTAAGGCGGGGGTAAGCGGTGAGGCTCAGATTGCGTCCATCAGCCAGAGTGTGGCGCGTTTCTCCTCTGCATCCGGCGTGGAGGTGGACAAGGTCGCTGAAGCCTTCGGGAAGCTGACCACAGACCCGACGTCGGGGCTGACGGCGATGGCTCGCCAGTTCCATAACGTGTCGGCGGAGCAGATTGCGTATGTTGCTCAGTTGCAGCGTTCCGGCGATGAAGCCGGGGCATTGCAGGCGGCGAACGAGGCCGCAACGAAAGGGTTTGATGACCAGACCCGCCGCCTGAAAGAGAACATGGGCACGCTGGAGACCTGGGCAGACAGGACTGCGCGGGCATTCAAATCCATGTGGGATGCGGTGCTGGATATTGGTCGTCCTGATACCGCGCAGGAGATGCTGATTAAGGCAGAGGCTGCGTATAAGAAAGCAGACGACATCTGGAATCTGCGCAAGGATGATTATTTTGTTAACGATGAAGCGCGGGCGCGTTACTGGGATGATCGTGAAAAGGCCCGTCTTGCGCTTGAAGCCGCCCGAAAGAAGGCTGAGCAGCAGACTCAACAGGACAAAAATGCGCAGCAGCAGAGCGATACCGAAGCGTCACGGCTGAAATATACCGAAGAGGCGCAGAAGGCTTACGAACGGCTGCAGACGCCGCTGGAGAAATATACCGCCCGTCAGGAAGAACTGAACAAGGCACTGAAAGACGGGAAAATCCTGCAGGCGGATTACAACACGCTGATGGCGGCGGCGAAAAAGGATTATGAAGCGACGCTGAAAAAGCCGAAACAGTCCAGCGTGAAGGTGTCTGCGGGCGATCGTCAGGAAGACAGTGCTCATGCTGCCCTGCTGACGCTTCAGGCAGAACTCCGGACGCTGGAGAAGCATGCCGGAGCAAATGAGAAAATCAGCCAGCAGCGCCGGGATTTGTGGAAGGCGGAGAGTCAGTTCGCGGTACTGGAGGAGGCGGCGCAACGTCGCCAGCTGTCTGCACAGGAGAAATCCCTGCTGGCGCATAAAGATGAGACGCTGGAGTACAAACGCCAGCTGGCTGCACTTGGCGACAAGGTTACGTATCAGGAGCGCCTGAACGCGCTGGCGCAGCAGGCGGATAAATTCGCACAGCAGCAACGGGCAAAACGGGCCGCCATTGATGCGAAAAGCCGGGGGCTGACTGACCGGCAGGCAGAACGGGAAGCCACGGAACAGCGCCTGAAGGAACAGTATGGCGATAATCCGCTGGCGCTGAATAACGTCATGTCAGAGCAGAAAAAGACCTGGGCGGCTGAAGACCAGCTTCGCGGGAACTGGATGGCAGGCCTGAAGTCCGGCTGGAGTGAGTGGGAAGAGAGCGCCACGGACAGTATGTCGCAGGTAAAAAGTGCAGCCACGCAGACCTTTGATGGTATTGCACAGAATATGGCGGCGATGCTGACCGGCAGTGAGCAGAACTGGCGCAGCTTCACCCGTTCCGTGCTGTCCATGATGACAGAAATTCTGCTTAAGCAGGCAATGGTGGGGATTGTCGGGAGTATCGGCAGCGCCATTGGCGGGGCTGTTGGTGGCGGCGCATCCGCGTCAGGCGGTACAGCCATTCAGGCCGCTGCGGCGAAATTCCATTTTGCAACCGGAGGATTTACGGGAACCGGCGGCAAATATGAGCCAGCGGGGATTGTTCACCGTGGTGAGTTTGTCTTCACGAAGGAGGCAACCAGCCGGATTGGCGTGGGGAATCTTTACCGGCTGATGCGCGGCTATGCCACCGGCGGTTATGTCGGTACACCGGGCAGCATGGCAGACAGCCGGTCGCAGGCGTCCGGGACGTTTGAGCAGAATAACCATGTGGTGATTAACAACGACGGCACGAACGGGCAGATAGGTCCGGCTGCTCTGAAGGCGGTGTATGACATGGCCCGCAAGGGTGCCCGTGATGAAATTCAGACACAGATGCGTGATGGTGGCCTGTTCTCCGGAGGTGGACGATGAAGACCTTCCGCTGGAAAGTGAAACCCGGTATGGATGTGGCTTCGGTCCCTTCTGTAAGAAAGGTGCGCTTTGGTGATGGCTATTCTCAGCGAGCGCCTGCCGGGCTGAATGCCAACCTGAAAACGTACAGCGTGACGCTTTCTGTCCCCCGTGAGGAGGCCACGGTACTGGAGTCGTTTCTGGAAGAGCACGGGGGCTGGAAATCCTTTCTGTGGACGCCGCCTTATGAGTGGCGGCAGATAAAGGTGACCTGCGCAAAATGGTCGTCGCGGGTCAGTATGCTGCGTGTTGAGTTCAGCGCAGAGTTTGAACAGGTGGTGAACTGATGCAGGATATCCGGCAGGAAACACTGAATGAATGCACCCGTGCGGAGCAGTCGGCCAGCGTGGTGCTCTGGGAAATCGACCTGACAGAGGTCGGTGGAGAACGTTATTTTTTCTGTAATGAGCAGAACGAAAAAGGTGAGCCGGTCACCTGGCAGGGGCGACAGTATCAGCCGTATCCCATTCAGGGGAGCGGTTTTGAACTGAATGGCAAAGGCACCAGTACGCGCCCCACGCTGACGGTTTCTAACCTGTACGGTATGGTCACCGGGATGGCGGAAGATATGCAGAGTCTGGTCGGCGGAACGGTGGTCCGGCGTAAGGTTTACGCCCGTTTTCTGGATGCGGTGAACTTCGTCAACGGAAACAGTTACGCCGATCCGGAGCAGGAGGTGATCAGCCGCTGGCGCATTGAGCAGTGCAGCGAACTGAGCGCGGTGAGTGCCTCCTTTGTACTGTCCACGCCGACGGAAACGGATGGCGCTGTTTTTCCGGGACGTATCATGCTGGCCAACACCTGCACCTGGACCTATCGCGGTGACGAGTGCGGTTATAGCGGTCCGGCTGTCGCGGATGAATATGACCAGCCAACGTCCGATATCACGAAGGATAAATGCAGCAAATGCCTGAGCGGTTGTAAGTTCCGCAATAACGTCGGCAACTTTGGCGGCTTCCTTTCCATTAACAAACTTTCGCAGTAAATCCCATGACACAGACAGAATCAGCGATTCTGGCGCACGCCCGGCGATGTGCGCCAGCGGAGTCGTGCGGCTTCGTGGTAAGCACGCCGGAGGGGGAAAGATATTTCCCCTGCGTGAATATCTCCGGTGAGCCGGAGGCTATTTCCGTATGTCGCCGGAAGACTGGCTGCAGGCAGAAATGCAGGGTGAGATTGTGGCGCTGGTCCACAGCCACCCCGGTGGTCTGCCCTGGCTGAGTGAGGCCGACCGGCGGCTGCAGGTGCAGAGTGATTTGCCGTGGTGGCTGGTCTGCCGGGGGACGATTCATAAGTTCCGCTGTGTGCCGCATCTCACCGGGCGGCGCTTTGAGCACGGTGTGACGGACTGTTACACACTGTTCCGGGC

*TPM ‘Scrambled’ parS DNA (1717 bp)*

GGGGATCCTCGAGCCCGGGGCGGCCGCGCCTGCCACCATACCCACGCCGAAACAAGCGCTCATGAGCCCGAAGTGGCGAGCCCGATCTTCCCCATCGGTGATGTCGGCGATATAGGCGCCAGCAACCGCACCTGTGGCGCCGGTGATGCCGGCCACGATGCGTCCGGCGTAGAGGATCGAGATCTCGATCCCGCGAAATTAATACGACTCACTATAGGGGAATTGTGAGCGGATAACAATTCCCCTCTAGAAATAATTTTGTTTAACTTTAAGAAGGAGATATACCATGGCTAAAGGCCTTGGAAAAGGGATTAATGCGTTATTTAATCAGGTAGATTTGTCTGAAGAGACAGTTGAAGAAATTAAAATTGCCGATTTACGCCCTAATCCTTATCAGCCAAGAAAACACTTTGATGACGAGGCATTAGCTGAACTAAAAGAATCTGTGCTGCAGCATGGCATTCTTCAGCCGCTTATCGTCAGAAAATCTTTAAAAGGCTATGATATTGTTGCGGGTGAACGGCGTTTTCGAGCGGCAAAGCTGGCAGGTTTAGATACAGTTCCGGCCATTGTCCGTGAATTATCAGAGGCGTTAATGAGGGAAATTGCTTTATTAGAAAACCTTCAGCGTGAAGATTTATCTCCGCTTGAAGAGGCTCAGGCATATGACTCCCTTTTGAAACACTTAGATCTCACACAAGAGCAGCTTGCCAAACGTCTTGGGAAAAGCAGACCGCATATTGCGAATCATTTAAGACTGCTGACACTGCCAGAAAATATTCAACAGCTTATTGCCGAAGGCACGCTTTCTATGGGACATGGACGCACGCTTCTTGGCTTAAAAAACAAAAATAAGCTTGAACCGCTGGTACAAAAAGTGATTGCGGAGCAGCTCAATGTTCGCCAACTTGAGCAGCTGATTCAGCAGTTGAATCAGAACGTGCCCAGGGAGACAAAGAAAAAAGAACCTGTGAAAGATGCGGTTCTAAAAGAACGGGAATCCTATCTCCAAAATTATTTTGGAACAACAGTTAATATTAAAAGACAGAAGAAAAAAGGCAAAATCGAAATTGAATTTTTCTCTAATGAAGACCTTGACCGGATTTTAGAGCTTTTGTCTGAACGAGAATCATAAGGATCCGAATTCGAGCTCCGTCGACAAGCTTCCGGCCGCAGTCGAGCACCACCACCACCACCACTGAGATCCGGCTGCTAACAAAGCCCGAAAGGAAGCTGAGTTGGCTGCTGCCACCGCTGAGCAATAACTAGCATAACCCCTTGGGGCCTCTAAACGGGTCTTGAGGGGTTTTTTGCTGAAAGGAGGAACTATATCCGGATTGGCGAATGGGACGCGCCCTGTAGCGGCGCATTAAGCGCGGCGGGTGTGGTGGTTACGCGCAGCGTGACCGCTACACTTGCCAGCGCCCTAGCGCCCGCTCCTTTCGCTTTCTTCCCTTCCTTTCTCGCCACGTTCGCCGGCTTTCCCCGTCAAGCTCTAAATCGGGGGCTCCCTTTAGGGTTCCGATTTAGTGCTTTACGGCACCTCGACCCCAAAAAACTTGATTAGGGTGATGGTTCACGTAGTGGGCCATCGCCCTGATAGACGGTTTTTCGCCCTTTGACGTTGGAGTCCACGTTCTTTAATAGTGGACTCTTGTTCCAAACTGGAACAACACTCAACCCTATCTCGGTCTCGAGCCCGGGCCATGGGATCCCC

*TPM 1x parS DNA (1717 bp)*

GGGGATCCTCGAGCCCGGGGCGGCCGCGCCTGCCACCATACCCACGCCGAAACAAGCGCTCATGAGCCCGAAGTGGCGAGCCCGATCTTCCCCATCGGTGATGTCGGCGATATAGGCGCCAGCAACCGCACCTGTGGCGCCGGTGATGCCGGCCACGATGCGTCCGGCGTAGAGGATCGAGATCTCGATCCCGCGAAATTAATACGACTCACTATAGGGGAATTGTGAGCGGATAACAATTCCCCTCTAGAAATAATTTTGTTTAACTTTAAGAAGGAGATATACCATGGCTAAAGGCCTTGGAAAAGGGATTAATGCGTTATTTAATCAGGTAGATTTGTCTGAAGAGACAGTTGAAGAAATTAAAATTGCCGATTTACGCCCTAATCCTTATCAGCCAAGAAAACACTTTGATGACGAGGCATTAGCTGAACTAAAAGAATCTGTGCTGCAGCATGGCATTCTTCAGCCGCTTATCGTCAGAAAATCTTTAAAAGGCTATGATATTGTTGCGGGTGAACGGCGTTTTCGAGCGGCAAAGCTGGCAGGTTTAGATACAGTTCCGGCCATTGTCCGTGAATTATCAGAGGCGTTAATGAGGGAAATTGCTTTATTAGAAAACCTTCAGCGTGAAGATTTATCTCCGCTTGAAGAGGCTCAGGCATATGACTCCCTTTTGAAACACTTAGATCTCACACAAGAGCAGCTTGCCAAACGTCTTGGGAAAAGCAGACCGCATATTGCGAATCATTTAAGACTGCTGACACTGCCAGAAAATATTCAACAGCTTATTGCCGAAGGCACGCTTTCTATGGGACATGGACGCACGCTTCTTGGCTTAAAAAACAAAAATAAGCTTGAACCGCTGGTACAAAAAGTGATTGCGGAGCAGCTCAATGTTCGCCAACTTGAGCAGCTGATTCAGCAGTTGAATCAGAATGTTCCACGTGAAACAAAGAAAAAAGAACCTGTGAAAGATGCGGTTCTAAAAGAACGGGAATCCTATCTCCAAAATTATTTTGGAACAACAGTTAATATTAAAAGACAGAAGAAAAAAGGCAAAATCGAAATTGAATTTTTCTCTAATGAAGACCTTGACCGGATTTTAGAGCTTTTGTCTGAACGAGAATCATAAGGATCCGAATTCGAGCTCCGTCGACAAGCTTCCGGCCGCAGTCGAGCACCACCACCACCACCACTGAGATCCGGCTGCTAACAAAGCCCGAAAGGAAGCTGAGTTGGCTGCTGCCACCGCTGAGCAATAACTAGCATAACCCCTTGGGGCCTCTAAACGGGTCTTGAGGGGTTTTTTGCTGAAAGGAGGAACTATATCCGGATTGGCGAATGGGACGCGCCCTGTAGCGGCGCATTAAGCGCGGCGGGTGTGGTGGTTACGCGCAGCGTGACCGCTACACTTGCCAGCGCCCTAGCGCCCGCTCCTTTCGCTTTCTTCCCTTCCTTTCTCGCCACGTTCGCCGGCTTTCCCCGTCAAGCTCTAAATCGGGGGCTCCCTTTAGGGTTCCGATTTAGTGCTTTACGGCACCTCGACCCCAAAAAACTTGATTAGGGTGATGGTTCACGTAGTGGGCCATCGCCCTGATAGACGGTTTTTCGCCCTTTGACGTTGGAGTCCACGTTCTTTAATAGTGGACTCTTGTTCCAAACTGGAACAACACTCAACCCTATCTCGGTCTCGAGCCCGGGCCATGGGATCCCC

*AFM No parS DNA (2961bp)*

CTAAATTGTAAGCGTTAATATTTTGTTAAAATTCGCGTTAAATTTTTGTTAAATCAGCTCATTTTTTAACCAATAGGCCGAAATCGGCAAAATCCCTTATAAATCAAAAGAATAGACCGAGATAGGGTTGAGTGTTGTTCCAGTTTGGAACAAGAGTCCACTATTAAAGAACGTGGACTCCAACGTCAAAGGGCGAAAAACCGTCTATCAGGGCGATGGCCCACTACGTGAACCATCACCCTAATCAAGTTTTTTGGGGTCGAGGTGCCGTAAAGCACTAAATCGGAACCCTAAAGGGAGCCCCCGATTTAGAGCTTGACGGGGAAAGCCGGCGAACGTGGCGAGAAAGGAAGGGAAGAAAGCGAAAGGAGCGGGCGCTAGGGCGCTGGCAAGTGTAGCGGTCACGCTGCGCGTAACCACCACACCCGCCGCGCTTAATGCGCCGCTACAGGGCGCGTCCCATTCGCCATTCAGGCTGCGCAACTGTTGGGAAGGGCGATCGGTGCGGGCCTCTTCGCTATTACGCCAGCTGGCGAAAGGGGGATGTGCTGCAAGGCGATTAAGTTGGGTAACGCCAGGGTTTTCCCAGTCACGACGTTGTAAAACGACGGCCAGTGAGCGCGCGTAATACGACTCACTATAGGGCGAATTGGGTACCGGGCCCCCCCTCGAGGTCGACGGTATCGATAAGCTTGATATCGAATTCCTGCAGCCCGGGGGATCCACTAGTTCTAGAGCGGCCGCCACCGCGGTGGAGCTCCAGCTTTTGTTCCCTTTAGTGAGGGTTAATTGCGCGCTTGGCGTAATCATGGTCATAGCTGTTTCCTGTGTGAAATTGTTATCCGCTCACAATTCCACACAACATACGAGCCGGAAGCATAAAGTGTAAAGCCTGGGGTGCCTAATGAGTGAGCTAACTCACATTAATTGCGTTGCGCTCACTGCCCGCTTTCCAGTCGGGAAACCTGTCGTGCCAGCTGCATTAATGAATCGGCCAACGCGCGGGGAGAGGCGGTTTGCGTATTGGGCGCTCTTCCGCTTCCTCGCTCACTGACTCGCTGCGCTCGGTCGTTCGGCTGCGGCGAGCGGTATCAGCTCACTCAAAGGCGGTAATACGGTTATCCACAGAATCAGGGGATAACGCAGGAAAGAACATGTGAGCAAAAGGCCAGCAAAAGGCCAGGAACCGTAAAAAGGCCGCGTTGCTGGCGTTTTTCCATAGGCTCCGCCCCCCTGACGAGCATCACAAAAATCGACGCTCAAGTCAGAGGTGGCGAAACCCGACAGGACTATAAAGATACCAGGCGTTTCCCCCTGGAAGCTCCCTCGTGCGCTCTCCTGTTCCGACCCTGCCGCTTACCGGATACCTGTCCGCCTTTCTCCCTTCGGGAAGCGTGGCGCTTTCTCATAGCTCACGCTGTAGGTATCTCAGTTCGGTGTAGGTCGTTCGCTCCAAGCTGGGCTGTGTGCACGAACCCCCCGTTCAGCCCGACCGCTGCGCCTTATCCGGTAACTATCGTCTTGAGTCCAACCCGGTAAGACACGACTTATCGCCACTGGCAGCAGCCACTGGTAACAGGATTAGCAGAGCGAGGTATGTAGGCGGTGCTACAGAGTTCTTGAAGTGGTGGCCTAACTACGGCTACACTAGAAGGACAGTATTTGGTATCTGCGCTCTGCTGAAGCCAGTTACCTTCGGAAAAAGAGTTGGTAGCTCTTGATCCGGCAAACAAACCACCGCTGGTAGCGGTGGTTTTTTTGTTTGCAAGCAGCAGATTACGCGCAGAAAAAAAGGATCTCAAGAAGATCCTTTGATCTTTTCTACGGGGTCTGACGCTCAGTGGAACGAAAACTCACGTTAAGGGATTTTGGTCATGAGATTATCAAAAAGGATCTTCACCTAGATCCTTTTAAATTAAAAATGAAGTTTTAAATCAATCTAAAGTATATATGAGTAAACTTGGTCTGACAGTTACCAATGCTTAATCAGTGAGGCACCTATCTCAGCGATCTGTCTATTTCGTTCATCCATAGTTGCCTGACTCCCCGTCGTGTAGATAACTACGATACGGGAGGGCTTACCATCTGGCCCCAGTGCTGCAATGATACCGCGAGACCCACGCTCACCGGCTCCAGATTTATCAGCAATAAACCAGCCAGCCGGAAGGGCCGAGCGCAGAAGTGGTCCTGCAACTTTATCCGCCTCCATCCAGTCTATTAATTGTTGCCGGGAAGCTAGAGTAAGTAGTTCGCCAGTTAATAGTTTGCGCAACGTTGTTGCCATTGCTACAGGCATCGTGGTGTCACGCTCGTCGTTTGGTATGGCTTCATTCAGCTCCGGTTCCCAACGATCAAGGCGAGTTACATGATCCCCCATGTTGTGCAAAAAAGCGGTTAGCTCCTTCGGTCCTCCGATCGTTGTCAGAAGTAAGTTGGCCGCAGTGTTATCACTCATGGTTATGGCAGCACTGCATAATTCTCTTACTGTCATGCCATCCGTAAGATGCTTTTCTGTGACTGGTGAGTACTCAACCAAGTCATTCTGAGAATAGTGTATGCGGCGACCGAGTTGCTCTTGCCCGGCGTCAATACGGGATAATACCGCGCCACATAGCAGAACTTTAAAAGTGCTCATCATTGGAAAACGTTCTTCGGGGCGAAAACTCTCAAGGATCTTACCGCTGTTGAGATCCAGTTCGATGTAACCCACTCGTGCACCCAACTGATCTTCAGCATCTTTTACTTTCACCAGCGTTTCTGGGTGAGCAAAAACAGGAAGGCAAAATGCCGCAAAAAAGGGAATAAGGGCGACACGGAAATGTTGAATACTCATACTCTTCCTTTTTCAATATTATTGAAGCATTTATCAGGGTTATTGTCTCATGAGCGGATACATATTTGAATGTATTTAGAAAAATAAACAAATAGGGGTTCCGCGCACATTTCCCCGAAAAGTGCCAC

*AFM 1x parS DNA (3557 bp)*

CTAAATTGTAAGCGTTAATATTTTGTTAAAATTCGCGTTAAATTTTTGTTAAATCAGCTCATTTTTTAACCAATAGGCCGAAATCGGCAAAATCCCTTACTCGACTGCGGCCGGAAGCTTGTCGACGGAGCTCGAATTCGGATCCTTATGATTCTCGTTCAGACAAAAGCTCTAAAATCCGGTCAAGGTCTTCATTAGAGAAAAATTCAATTTCGATTTTGCCTTTTTTCTTCTGTCTTTTAATATTAACTGTTGTTCCAAAATAATTTTGGAGATAGGATTCCCGTTCTTTTAGAACCGCATCTTTCACAGGTTCTTTTTTCTTTGTTTCACGTGGAACATTCTGATTCAACTGCTGAATCAGCTGCTCAAGTTGGCGAACATTGAGCTGCTCCGCAATCACTTTTTGTACCAGCGGTTCAAGCTTATTTTTGTTTTTTAAGCCAAGAAGCGTGCGTCCATGTCCCATAGAAAGCGTGCCTTCGGCAATAAGCTGTTGAATATTTTCTGGCAGTGTCAGCAGTCTTAAATGATTCGCAATATGCGGTCTGCTTTTCCCAAGACGTTTGGCAAGCTGCTCTTGTGTGAGATCTAAGTGTTTCAAAAGGGAGTCATATGCCTGAGCCTCTTCAAGCGGAGATAAATCTTCACGCTGAAGGTTTTCTAATAAAGCAATTTCCCTCATTAACGCCTCTGATAATTCACGGACAATGGCCGGAACTGTATCTAAACCTGCCAGCTTTGCCGCTCGAAAACGCCGTTCACCCGCAACAATATCATAGCCTTTTAAAGATTTTCTGACGATAAGCGGCTGAAGAATGCCATGCTGCAGCACAGATTCTTTTAGTTCAGCTAATGCCTCGTCATCAAAGTGTTTTCTTGGCTGATAAGGATTAGGGCGTAAATCGGCAATTTTAATTTCTTCAACTGTCTCTTCAGACAAATCTACCTGATTAAATAACGCATTAATCCCTTTTCCAAGGCCTTTAGCCATGGTATATCTCCTTCTTAAAGTTAAACAAAATTATTTCTAGAaGGGAATTGTTATCCGCTCACAATTCCCCTATAGTGAGTCGTATTAATTTCGCGGGATCGAGATCTCGATCCTCTACGCCGGACGCATCGTGGCCGGCATCACCGGCGCCACAGGTGCGGTTGCTGGCGCCTATATCGCCGACATCACCGATGGGGAAGATCGGGCTCGCCACTTCGGGCTCATGAGCGCTTGTTTCGGCGTGGGTATGGTGGCAGGCGCGGCCCCCCCTCGAGGTCGACGGTATCGATAAGCTTGATATCGAATTCCTGCAGCCCGGGGGATCCACTAGTTCTAGAGCGGCCGCCACCGCGGTGGAGCTCCAGCTTTTGTTCCCTTTAGTGAGGGTTAATTGCGCGCTTGGCGTAATCATGGTCATAGCTGTTTCCTGTGTGAAATTGTTATCCGCTCACAATTCCACACAACATACGAGCCGGAAGCATAAAGTGTAAAGCCTGGGGTGCCTAATGAGTGAGCTAACTCACATTAATTGCGTTGCGCTCACTGCCCGCTTTCCAGTCGGGAAACCTGTCGTGCCAGCTGCATTAATGAATCGGCCAACGCGCGGGGAGAGGCGGTTTGCGTATTGGGCGCTCTTCCGCTTCCTCGCTCACTGACTCGCTGCGCTCGGTCGTTCGGCTGCGGCGAGCGGTATCAGCTCACTCAAAGGCGGTAATACGGTTATCCACAGAATCAGGGGATAACGCAGGAAAGAACATGTGAGCAAAAGGCCAGCAAAAGGCCAGGAACCGTAAAAAGGCCGCGTTGCTGGCGTTTTTCCATAGGCTCCGCCCCCCTGACGAGCATCACAAAAATCGACGCTCAAGTCAGAGGTGGCGAAACCCGACAGGACTATAAAGATACCAGGCGTTTCCCCCTGGAAGCTCCCTCGTGCGCTCTCCTGTTCCGACCCTGCCGCTTACCGGATACCTGTCCGCCTTTCTCCCTTCGGGAAGCGTGGCGCTTTCTCATAGCTCACGCTGTAGGTATCTCAGTTCGGTGTAGGTCGTTCGCTCCAAGCTGGGCTGTGTGCACGAACCCCCCGTTCAGCCCGACCGCTGCGCCTTATCCGGTAACTATCGTCTTGAGTCCAACCCGGTAAGACACGACTTATCGCCACTGGCAGCAGCCACTGGTAACAGGATTAGCAGAGCGAGGTATGTAGGCGGTGCTACAGAGTTCTTGAAGTGGTGGCCTAACTACGGCTACACTAGAAGGACAGTATTTGGTATCTGCGCTCTGCTGAAGCCAGTTACCTTCGGAAAAAGAGTTGGTAGCTCTTGATCCGGCAAACAAACCACCGCTGGTAGCGGTGGTTTTTTTGTTTGCAAGCAGCAGATTACGCGCAGAAAAAAAGGATCTCAAGAAGATCCTTTGATCTTTTCTACGGGGTCTGACGCTCAGTGGAACGAAAACTCACGTTAAGGGATTTTGGTCATGAGATTATCAAAAAGGATCTTCACCTAGATCCTTTTAAATTAAAAATGAAGTTTTAAATCAATCTAAAGTATATATGAGTAAACTTGGTCTGACAGTTACCAATGCTTAATCAGTGAGGCACCTATCTCAGCGATCTGTCTATTTCGTTCATCCATAGTTGCCTGACTCCCCGTCGTGTAGATAACTACGATACGGGAGGGCTTACCATCTGGCCCCAGTGCTGCAATGATACCGCGAGACCCACGCTCACCGGCTCCAGATTTATCAGCAATAAACCAGCCAGCCGGAAGGGCCGAGCGCAGAAGTGGTCCTGCAACTTTATCCGCCTCCATCCAGTCTATTAATTGTTGCCGGGAAGCTAGAGTAAGTAGTTCGCCAGTTAATAGTTTGCGCAACGTTGTTGCCATTGCTACAGGCATCGTGGTGTCACGCTCGTCGTTTGGTATGGCTTCATTCAGCTCCGGTTCCCAACGATCAAGGCGAGTTACATGATCCCCCATGTTGTGCAAAAAAGCGGTTAGCTCCTTCGGTCCTCCGATCGTTGTCAGAAGTAAGTTGGCCGCAGTGTTATCACTCATGGTTATGGCAGCACTGCATAATTCTCTTACTGTCATGCCATCCGTAAGATGCTTTTCTGTGACTGGTGAGTACTCAACCAAGTCATTCTGAGAATAGTGTATGCGGCGACCGAGTTGCTCTTGCCCGGCGTCAATACGGGATAATACCGCGCCACATAGCAGAACTTTAAAAGTGCTCATCATTGGAAAACGTTCTTCGGGGCGAAAACTCTCAAGGATCTTACCGCTGTTGAGATCCAGTTCGATGTAACCCACTCGTGCACCCAACTGATCTTCAGCATCTTTTACTTTCACCAGCGTTTCTGGGTGAGCAAAAACAGGAAGGCAAAATGCCGCAAAAAAGGGAATAAGGGCGACACGGAAATGTTGAATACTCATACTCTTCCTTTTTCAATATTATTGAAGCATTTATCAGGGTTATTGTCTCATGAGCGGATACATATTTGAATGTATTTAGAAAAATAAACAAATAGGGGTTCCGCGCACATTTCCCCGAAAAGTGCCAC

**Supplementary References**

Taylor, J.A., Pastrana, C.L., Butterer, A., Pernstich, C., Gwynn, E.J., Sobott, F., Moreno-Herrero, F., and Dillingham, M.S. (2015). Specific and non-specific interactions of ParB with DNA: Implications for chromosome segregation. Nucleic Acids Res. *43*, 719–731.
