# Supplementary material for: CTP promotes efficient ParB-dependent DNA condensation by facilitating one-dimensional diffusion from parS
Source: eLife. 2021 Jul 12;10:e67554. doi: 10.7554/eLife.67554 (PMC8299390; doi:10.7554/eLife.67554)
Supplement: Supplementary file 2. [file elife-67554-supp2.docx]

**Supplementary File 2. DNA oligonucleotides used in this work**

| **Fragment** | **Oligonucleotide** | **Sequence** |
| --- | --- | --- |
| Annealed oligonucleotides with 2 *parS* sites | 30.P-up 2ParS XbaI-BsrGI | [Pho]CTAGCTGTACATTAATCAGAATGTTCCACGTGAAACAAAGAAAAAAGAACCTGTTCTAGACCTAGTCCAGGCTCAGTGTTCCACGTGAAACAGGTCCGAGTCCAATATGG |
|  | 31.P-down 2ParS XbaI-BsrGI | [Pho]CTAGCCATATTGGACTCGGACCTGTTTCACGTGGAACACTGAGCCTGGACTAGGTCTAGAACAGGTTCTTTTTTCTTTGTTTCACGTGGAACATTCTGATTAATGTACAG |
| 6 *parS* PCR fragment | 32.F pET28 PCR NdeI | GCGTAAGTCATATGCCGAAACAAGCGCTCATG |
|  | 33.R pET28 PCR NdeI | GCGTAAGTCATATGCGCATTAATCCCTTTTCC |
| 13 *parS* PCR fragment | 50.F pET28 37 SphI-BglI | GCGTAAGTGCATGCGCCATGCCGGCGCCTGCCACCATACCCAC |
|  | 51.R pET28 BglI-SphI | GCGTAAGTGCATGCGCCGGCATGGCGAACCGCATCTTTCACAGG |
| Lambda C1 fragment | 20Lambda_F_NotI | GCGTAAGTGCGGCCGCGGTGTGCTCCTTATTTATACATAACG |
|  | 21Lambda_R_SalI | GCGTAAGTGTCGACGCGACTTATCAACGCCCACAG |
| Lambda C2 fragment | 22Lambda_F_BamHI | CGGTTGCGGCAGAAAACAGCCGC |
|  | 23Lambda_R_NotI | GCGTAAGTGCGGCCGCCCGGAACAGTGTGTAACAGTC |
| Lambda C1-EcoRI fragment | 20Lambda_F_NotI | GCGTAAGTGCGGCCGCGGTGTGCTCCTTATTTATACATAACG |
|  | 176.Lambda_R_5Eco Sal | TAAGTGTCGACGAATTCATCCGAGAATTCCGATAGGAATTCCACGTTGAATTCTACGACGAATTCGCGACTTATCAACGCCCACAGC |
| Lambda C2-EcoRI fragment | 177.Lambda_F_5Eco BamHI | TAAGTGGATCCGAATTCATCCGAGAATTCCGATAGGAATTCCACGTTGAATTCTACGACGAATTCTCAACTGTGAGGAGGCTCACGG |
|  | 23Lambda_R_NotI | GCGTAAGTGCGGCCGCCCGGAACAGTGTGTAACAGTC |
| Central part of MT substrates | FMH_F_NotI_pET28-SpoOJ | GCGTAAGTGCGGCCGCCTGCATGTGTCAGAGGTTTTCAC |
|  | FMH_R_XhoI_pET28-SpoOJ | GCGTAAGTCTCGAGCAGATTGTACTGAGAGTGCACCA |
| Central part of MT EcoRI 7x *parS* DNA | 197.F-7parS-5EcoRI XhoI | GCGTAAGTCTCGAGCAGCGCCATCTGATCGTTG |
|  | 198.R-7parS-5EcoRI NotI | GGCGTAAGTGCGGCCGCTACCGATACTGCTGACCCAGC |
| MT handles | FMH_F2_NotI | GCGTAAGTGCGGCCGCGACTCACTATAGGGAGACCGGC |
|  | JOE_R1 | AGTAAGCGCCGTCAGACCAG |
| 1x *parS* PCR fragment and ‘scrambled’ PCR fragment for TPM DNA substrates | 73.R-TPM control XhoI | GCGTAAGTCTCGAGACCGAGATAGGGTTGAGTG |
|  | 74.F-TPM OK NotI | GCGTAAGTGCGGCCGCGCCTGCCACCATACCCAC |
| Bio tailed oligonucleotides | 27-P-XhoI-A | [pho]tcgagCCCGGGCCATGGGATCCCC |
|  | 26-XhoI-B | GGGGATCCCATGGCCCGGGc |
| Dig tailed oligonucleotides | 24P-NotI-A | [pho]ggccgcCCCGGGCTCGAGGATCCCC |
|  | 25-NotI-B | GGGGATCCTCGAGCCCGGGgc |
| AFM 1x *parS* PCR fragment | 74.F-TPM OK NotI | GCGTAAGTGCGGCCGCGCCTGCCACCATACCCAC |
|  | 46.R post SalI pET | CTCGACTGCGGCCGGAAG |
| *Scrambled parS* for NTP hydrolysis assay | parS-Scrambled-1 | CAGCAGTTGAATCAGAACGTGCCCAGGGAGACAAAGAAAAAAGAACCTGT |
|  | parS-Scrambled-2 | ACAGGTTCTTTTTTCTTTGTCTCCCTGGGCACGTTCTGATTCAACTGCTG |
| *1x parS* for NTP hydrolysis assay | parS-1 | CAGCAGTTGAATCAGAATGTTCCACGTGAAACAAAGAAAAAAGAACCTGT |
|  | parS-2 | ACAGGTTCTTTTTTCTTTGTTTCACGTGGAACATTCTGATTCAACTGCTG |
| *2x parS* for NTP hydrolysis assay | 28.2ParS XbaI up | CTAGCCTCGAGTTAATCAGAATGTTCCACGTGAAACAAAGAAAAAAGAACCTGTTCTAGACCTAGTCCAGGCTCAGTGTTCCACGTGAAACAGGTCCGAGTCCAATATGG |
|  | 29.2Pars XbaI down | CTAGCCATATTGGACTCGGACCTGTTTCACGTGGAACACTGAGCCTGGACTAGGTCTAGAACAGGTTCTTTTTTCTTTGTTTCACGTGGAACATTCTGATTAACTCGAGG |
